# Competition among co-infecting potyviral chimeras with adaptive, single-nucleotide differences in *HCPro*: Properties, and influence of environment factors on outcomes

**DOI:** 10.1371/journal.ppat.1014529

**Published:** 2026-08-18

**Authors:** Mongia Makki, María Fernández-Avilés, Francisco Tenllado, Tomás Canto

**Affiliations:** Department of Biotechnology, Margarita Salas Center for Biological Research (CIB), Spanish National Research Council, CSIC, Madrid, Spain; China Agricultural University, CHINA

## Abstract

We previously showed that two natural, single-amino acid mutations in the HCPro of a potyviral chimera, separately increased virulence by enhancing either its silencing-suppression or proteolytic functions. To investigate how beneficial variants generated during infection compete with non-mutant forms, or among each other, we co-expressed them in pairs, in *Nicotiana benthamiana*. We assessed how variants co-exist and influence each other in the cell, and whether altered abiotic (elevated temperature) or biotic (a silencing-impaired plant) environmental factors, affected local and systemic infection outcomes. In local tissues, our results are consistent with a model in which variants replicate and accumulate independently in co-infected cells: observed variant titers and ratios correlated with those that could be deduced from single infections. Trans-acting effects of one variant over the other were not observed. By contrast, environment parameters influenced not only local titers but also ratios, likely because of their differential effects on functions affected by the specific mutation of each variant. Additional mutations in *HCPro* appeared separately in systemic infections in specific plants, and induced either new substitutions in the protein, or in a putative small reverse reading frame. Thus, further adaptation could proceed through separate paths via the incorporation of distinct, novel mutations in the *HCPro* sequence in different plants.

## Author summary

Our work studies how potyviral chimera variants that differ in one, or at most two, single-nucleotide mutations that had been acquired naturally, interact with either non-mutant forms, or among each other, during co-infection of an experimental host. These mutations occur in the cistron encoding the viral protein HCPro, and each affects different aspects of its functions. We assessed how variant pairs influence each other within the plant cell during local co-infection, in the absence of cell-to-cell movement, with regard to titers and co-infection ratios. We also assessed the systemic outcomes of those co-infections, at the individual plant level. We propose a model that explains within-cell interactions, and also show that two environment parameters (one abiotic, one biotic) can affect both, local and systemic co-infection outcomes.

## Introduction

Most plant viruses organize in compact genomes that express proteins that are often multifunctional. This is not only for a matter of economy, but also because viruses face size constrains to their intercellular and systemic dispersion within plants, as well as among plants. This organization allows them to complete their infection cycles in compatible plants, and remain in the environment. However, viruses must also adapt to changes in that environment, be it the infection of new hosts, or of the same host under altered abiotic or biotic conditions, or harboring resistance traits [1]. In absence of mixed infections, adaptation is achieved by the incorporation of randomly-generated mutations that are beneficial to the new conditions. The latter may be difficulted by their compact, multifunctional organization, since mutations could be deleterious to one or another function. Some viral proteins appear to have hypervariable regions that could provide more receptive to adaptive mutations. This seems to be the case for the *Potyviral* VPg and, to a lesser degree, for the non-structural protein HCPro [2,3].

In any event, as the biological frame that facilitates virus replication and translation is provided largely by the plant, new mutant molecules, even deleterious ones, could remain in the viral population for a time; the beneficial ones, co-exist and out-compete the other forms to eventually prevail. How the latter is achieved has much to do with how viruses replicate in infected cells, disperse to other cells, and infect them.

Single nucleotide mutants generated by random mutation could be viewed in a way as extreme cases of mixed infection amongst almost identical viruses. However, a main difference with natural mixed infections between different viruses is that while in the former, mutant and nor mutant forms co-exist in the same replication complexes that created them, in the latter that is not the case. Mixed infections are important for virus evolution/adaptation through recombination events that require the physical switching between different genome templates during replication and thus, their co-localization in replication complexes, i.e., natural and lab-generated recombinant *Potyviruses* [4,5], respectively.

It is known that in nature the nearer two viruses are, the less likely that they may co-infect a plant. This is because prior infection by one virus excludes infection by a very similar virus, although it might not occur in simultaneous co-infection. This phenomenon has been the basis of the strategy of cross-protection of crops, in which plants are purposely infected with a mild strain or isolate of a virus, in order to prevent the same plants from becoming infected by a similar but more pathogenic strain [6]. The molecular basis of exclusion is not yet fully understood, although it involves the first virus to reach the cell interfering one way or another with early replication of any secondary infection by a similar virus [7–9]. This could depend on viral determinants. In this regard, the first N-terminal amino acids of HCPro could be involved in superinfection exclusion among *Potyvirus yituberosi* (potato virus Y, PVY) strains [10]. In any event, regardless of the mechanism, the infection exclusion phenomenon could provide a means for newly-generated beneficial mutants to prevail in the viral population.

On the other hand, mixed infections can also provide competitive benefits to co-infecting pairs, which help them persist [11,12]. In some instances, synergism leads to increased virulence, defined here as increased viral titers and/or infection symptoms, of one or both of the co-infecting viruses: one such example are potexvirus/potyvirus mixed infections, in which the silencing suppressor function of the potyviral non-structural protein HCPro enhances in trans the levels of a co-infecting potexvirus, relative to single infections [13,14]. Examples in the literature that relate synergism in mixed infections with trans-acting functional effects of a specific protein from one virus on the other virus are however, limited.

Therefore, during co-infection, exclusion would lead to the prevalence of one virus, but synergic effects could help both viruses co-exist. Co-infection outcomes will derive from their opposite effects, being influenced by host and environment.

*Potyvirus* genomes are made of a single, messenger-type poly-cistron RNA, of circa 10 kilobases, that can be directly translated by plant ribosomes into a single large polyprotein. The latter is processed co/post-translationally, to generate most final protein products. Some additional small proteins are generated through ribosome slippage on the RNA and, at least in one species, *Potyvirus rapae* (turnip mosaic virus), functional peptides are being expressed from small reverse open reading frames (rORFs) found in the negative RNA replication intermediate [15], although this strategy may not be extensive to other species, such as *P. plumpoxi* (plum pox virus, PPV; [16]). *Potyvirus* replication/translation and its movement among cells seem to be both tightly integrated, involving vesicular confinements that associate to different subcellular structures in the cytoplasm during these processes [17–20].

The second protein starting from the N-terminus of the polyprotein precursor is HCPro. From early studies it is known that HCPro is a soluble cytoplasmic homodimer (for review, see [21]) that in *Nicotiana benthamiana* epidermal cells also accumulates in dot-like inclusions anchored to the cytoplasm cortical endoplasmic reticulum, as well as in larger amorphous inclusions, and under certain conditions the microtubule cytoskeleton [22]. Among other functions, HCPro has a serine protease activity that separates it post-translationally from the remaining polyprotein downstream. It also suppresses silencing, and this function is itself multi-layered, since it achieves it through at least two separate molecular paths: by associating to small RNAs (sRNAs) [23], preferably of viral sequence (vsRNAs) and specific size (21–22 nt), and 5´-end adenines; [24,25], as well as by interfering with the methylation of sRNAs [25–27]. HCPro also mediates the non-persistent transmission of infection between plants through insect vectors by means that require its molecular interaction with factors from both, vector and infected cells in the donor plant [28]. Transmission efficiency correlates with viral titers in the donor plant [29,30]. Thus, through its functions, HCPro allows the systemic dispersal of infection inside a compatible host in titers are sufficient for the horizontal dispersal of infection by vectors, and also intervenes mechanistically in events related to the transmission process.

We had previously shown that a chimeric potyvirus in which the *P1-HCPro* bi-cistron of PVY replaced that of PPV in a cloned PPV-GFP construct was infectious in *N. benthamiana* plants, but its titers were a fraction of those of the parental virus [25]. By performing a series of mechanical passages, we found that this chimeric clonal virus could adapt further to its host, increasing titers and symptoms. Adaptation correlated with the separate acquisition in different plants of single amino acid substitutions in HCPro that affected either its silencing suppression function, or its proteolytic cleavage rate [31,32]. Using reverse genetics, we demonstrated that they were responsible for the increased virulence of the variants, which incorporated them, and when both were present in the same variant their effects were additive [32]. The word variant in this work refers to clonal chimeras that differ among themselves in one, or at most two, nucleotides in their *HCPro* cistron (as well as in their entire genomes), which result in amino acid differences. All of these mutations were acquired during the course of natural infections, and were incorporated by reverse genetics into the cloned virus.

Here, we investigated how nearly isogenic co-infecting clonal chimera variants carrying single beneficial mutations in the multifunctional protein HCPro, coexist and compete locally and systemically with either the non-mutant variant or among themselves, as well as adapt further. Hence, we co-expressed them in pairs in wild-type *N. benthamiana plants* (WT) at 23 °C (room temperature, RT), and studied both local infection and systemic outcomes. In the case of variant pairs each harboring a separate beneficial mutation, co-infection was studied also under two altered environment parameters: an abiotic one, high temperature, and a biotic one, using *dicer-like* (*Dcl2* and *4*)-deficient plants. Our results indicate that in local co-infections variants do not influence each other in the cell. However, environment parameters affect not only local variant titers but also their ratios, likely because of their differential effects on functions affected by their specific mutations. In systemic tissues, the chimera with higher fitness in the co-infecting pair prevailed, but further adaptation could proceed along separate paths, by the incorporation of distinct, novel mutations in the *HCPro* sequence in different plants.

## Results

### Co-infection titers in local tissues

**Fig 1:**
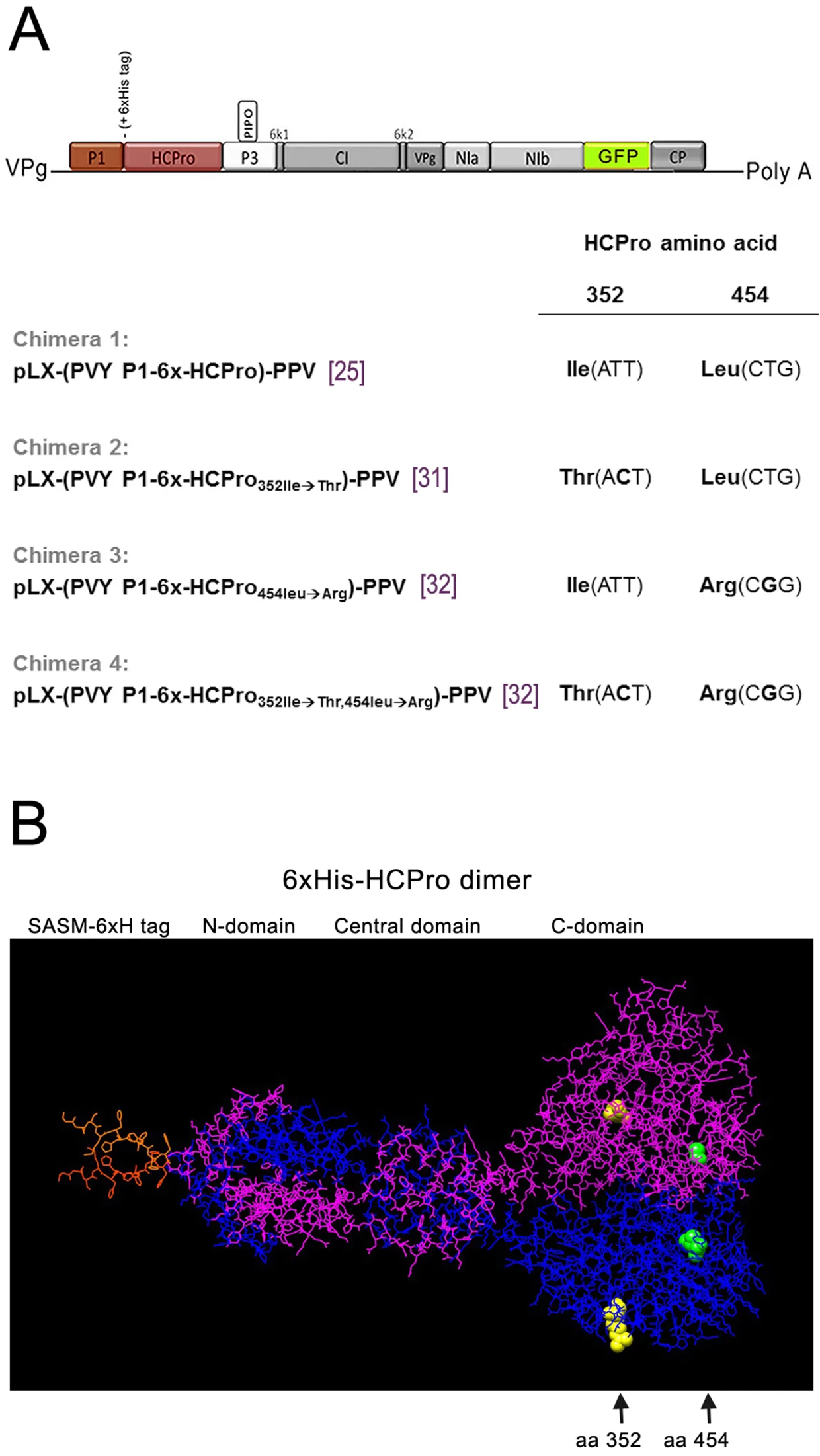
Characteristics of four potyvirus chimeras used in this study [32]. (A) Schematic representation of the viral genome and encoded products. Their genomes have a PVY *P1-HCPro* bi-cistron (indicated in red) in an otherwise PPV backbone (shown in gray) that also has an additional *GFP* cistron (shown in green). The four clonal chimeras are listed below, and they differ only at amino acids 352 an/or 454 of HCPro, as specified to the right. (B) The predicted conformation of the wild-type (WT) histidine-tagged HCPro homodimer (AlphaFold 3, edited with UCSF Chimera 1.19). It shows three domains: N-terminal, Central, and C-terminal. Amino acids 352Ile and 454Arg (highlighted in yellow and green, respectively) are located in the latter domain.

We had previously compared single infections by four clonal potyvirus chimera variants that contained either none (chimera 1, the non-mutant variant), one of two natural beneficial mutations in HCPro, at either amino acids 352 Ile (A**T**T) →Thr (A**C**T, chimera 2), 454 Leu (C**T**G) →Arg (C**G**G, chimera 3), or the two combined (chimera 4). One of the mutations (352Thr) increased its silencing suppression function, while the other one (454Arg) enhanced its proteolytic cleavage rate ([32]; Fig 1A). The two sites fall within the C-terminal domain of HCPro (Fig 1B).

**Fig 2:**
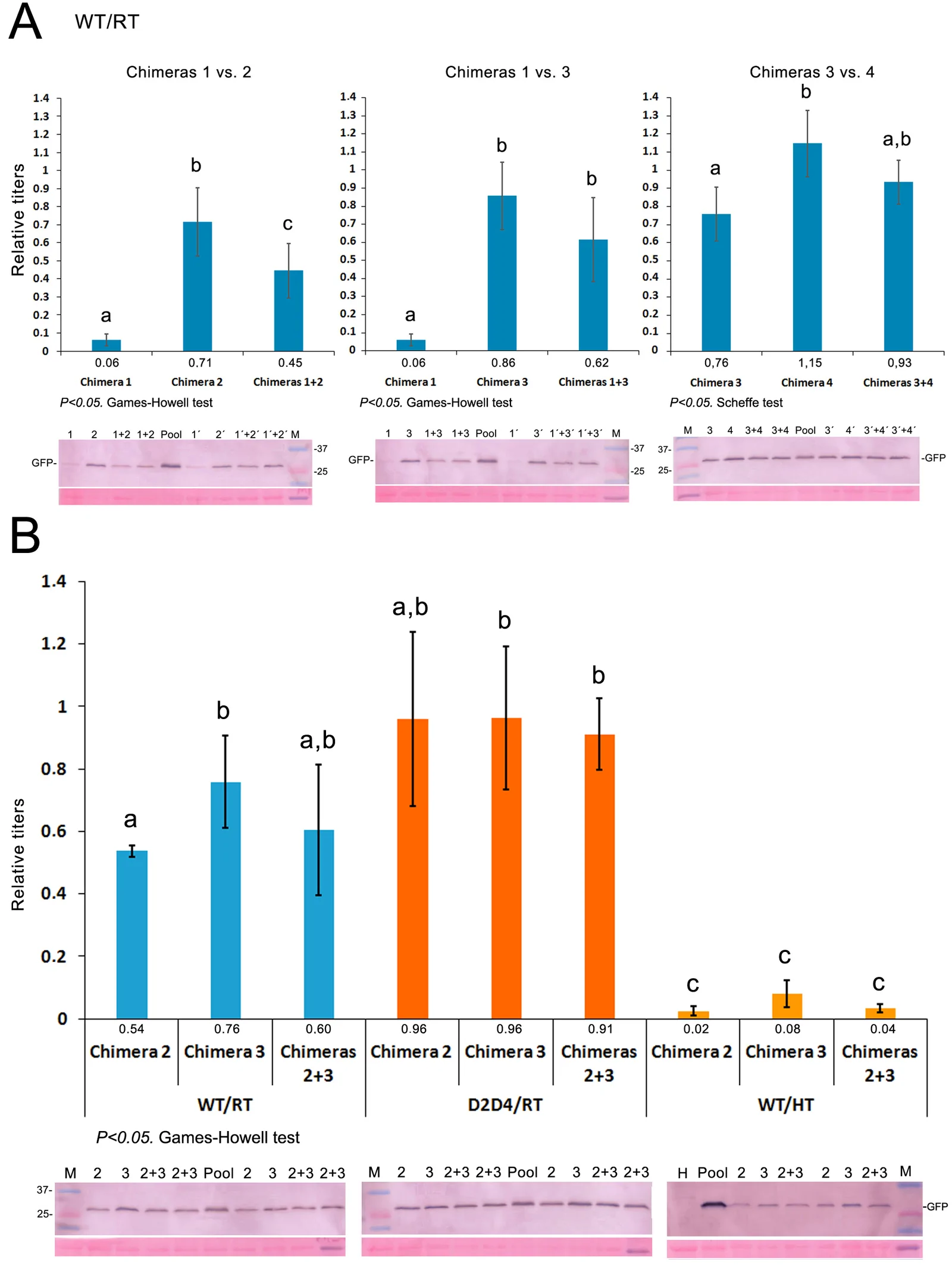
Assessment of chimera titers in local single infections and in co-infections. (A) Relative viral titers in single infections and in co-infections of chimera pairs 1 and 2 (chart to the left), 1 and 3 (central chart), and 3 and 4 (chart to the right), in wild-type plants (WT) kept at 23 °C (room temperature, RT) were determined by western blot detection and quantitation of GFP in total protein extracts from agroinfiltrated tissues at 5 days post-infiltration. Values in the three charts were relativized to the same pool sample, that was given the arbitrary value of 1. (B) Relative local titers of chimeras 2 and 3 in single infections and in co-infections, under three environments: WT/RT, wild-type plants (WT) at 23 °C (room temperature, RT); WT/HT conditions, in which an abiotic parameter, temperature has been increased to 29 °C (high temperature, HT); D2D4/RT conditions, in which a biotic parameter has been altered (use of silencing-compromised, *Dcl* 2,4-silenced plants instead of WT plants). Titers were assessed by western blot detection and quantitation of GFP in total protein extracts from agroinfiltrated tissues at 5 days post-infiltration. Chart values were relativized to a pool sample that was given the arbitrary value of 1. Below each chart in (A) and (B) appear western blot examples for each case, as well as the Ponceau S-stained rubisco bands below, for controls of loading. Charts show combined data from two independents experiments and bars indicate the standard deviation. The number of replicates (n) for each chart is shown in the last page of the S1 Raw Images file. Statistical tests and their significance at *p < 0.05* are indicated below the chart.

We now assessed, first, how pairs of chimera variants that differ only in one single nucleotide in their whole genomes (and amino acid in HCPro) would co-exist and influence each other in local co-infections, in the absence of cell-to-cell movement: variants 1 + 2, 1 + 3, 3 + 4. To do that we co-expressed them by agroinfiltration at equal optical densities (OD) at 600 nm of 0.3 in leaf tissues under WT/RT environment conditions, and measured virus titers in infiltrated patches at 5 days post-infiltration (dpi), relative to single infections. Our data confirm that in single infections mutations 352Ile → Thr and 454Leu → Arg were each beneficial (Fig 2A, single infection of chimera 2 vs. chimera 1, and of chimera 3 vs. chimera 1, left and central charts, respectively), and also that their effects were additive when both were present in the same variant (Fig 2A, single infection of chimera 4 vs. chimeras 3, or 2, chart to the right). By contrast, in co-infections, no evidence of synergism in titers was observed (Fig 2A).

We then assessed local co-infection of chimeras 2 + 3, which each harbor a different beneficial mutation, to see how they would co-exist and compete with each other, and whether any synergistic effect would be observed. In addition to standard conditions (WT/RT), relative local titers also were determined under two additional environment conditions: an abiotic one, elevated temperature (WT plants at 29 °C; WT/HT), and a biotic one, *Dcl2-* and *Dcl4*-deficient plants at standard temperature (D2D4/RT), to assess their effect on titers. In D2D4/RT conditions, titers of the two chimeras became comparable (Fig 2B). Elevated temperature (WT/HT conditions) reduced titers significantly, relative to WT/RT (Fig 2B). Overall titers in co-infection of chimeras 2 + 3 appeared to be intermediate of those in single infections under the three environments, and no evidence of synergism on titers was observed either (Fig 2B).

### Two separate approaches to determine and validate chimera variant co-infection ratios

The genomes of the chimera variants differ only in one, or at most two nucleotides, situated in the 3´-third of the *HCPro* cistron: Therefore, to assess variant ratios in co-infections we had to sequence those specific sites. We followed two separate approaches to obtain and validate our data, which started both from DNAse-treated total RNAs extracted from infected tissues: **a)** RT-PCR amplification of the 3´ region of *HCPro*, and either cloning the PCR product and sequencing several (≥10) individual clones, or direct sequencing of the PCR fragments, and quantitative electropherogram analysis of the polymorphic nucleotide sites; **b)** synthesis of double-stranded (ds) cDNAs of the 3´ region of *HCPro* from the total RNAs, and their sequencing with nanopore technology.

We assessed the feasibility of determining variant ratios by RT-PCR amplification (method **a**) from total plant RNAs of an *HCPro* fragment of circa 500 nt that contained the triplets encoding amino acids 352 and 454. The fragment was cloned, and multiple clones were individually sequenced. In the case of co-infections of chimeras 2 + 3, since they differed in two nucleotides it was possible to assess not only their ratios but also whether there were any homologous recombinants, thus creating chimeras 1 or 4. In co-infections of chimeras 1 + 2, 1 + 3, or 3 + 4 that was not possible to assess, as they differed in only one nucleotide. We found an unlikely proportion of reads in chimeras 2 + 3 co-infections that corresponded to homologous recombinants, (S1A Fig). However, when we amplified fragments from total RNAs extracted from single chimera 2 or chimera 3 infections combined, 25% of the clones also contained homologous recombinants that had been generated through template-switching by Phusion polymerase, even under optimized RT-PCR conditions (S1B Fig). Similar results were obtained when the chimera 2- and chimera 3-encoding plasmids were used as templates for PCR amplification of the *HCPro* fragment without reverse transcription, with 21% of the clones containing homologous recombinants. This is in accordance to previously described observations [33], and indicates that most if not all the homologous recombinants detected were artifactual. However, we found that discarding the homologous recombinant clones did not alter relative variant proportions among non-recombinant clones, indicating that the effect of the Phusion template-switching was neutral in this regard (S1C Fig, third vs. second bars). Furthermore, when we compared variant ratios obtained from Sanger sequencing of individual clones, including the Phusion-generated recombinant ones, with those deducted from the direct sequencing of PCR fragments and electropherogram polymorphism analysis (QSV software, [34]) both were similar (S1C Fig, second vs. first bars). Therefore, relative ratios in co-infections of chimeras 2 + 3 could be assessed by the direct sequencing of RT-PCR *HCPro* fragments. The data were contrasted with those obtained by nanopore sequencing ds cDNAs (method **b**) of *HCPro* obtained without a PCR amplification step. Method **a** could not inform on the presence of natural homologous recombinants, but method **b** could.

### Determination of variant ratios in local co-infections

**Fig 3:**
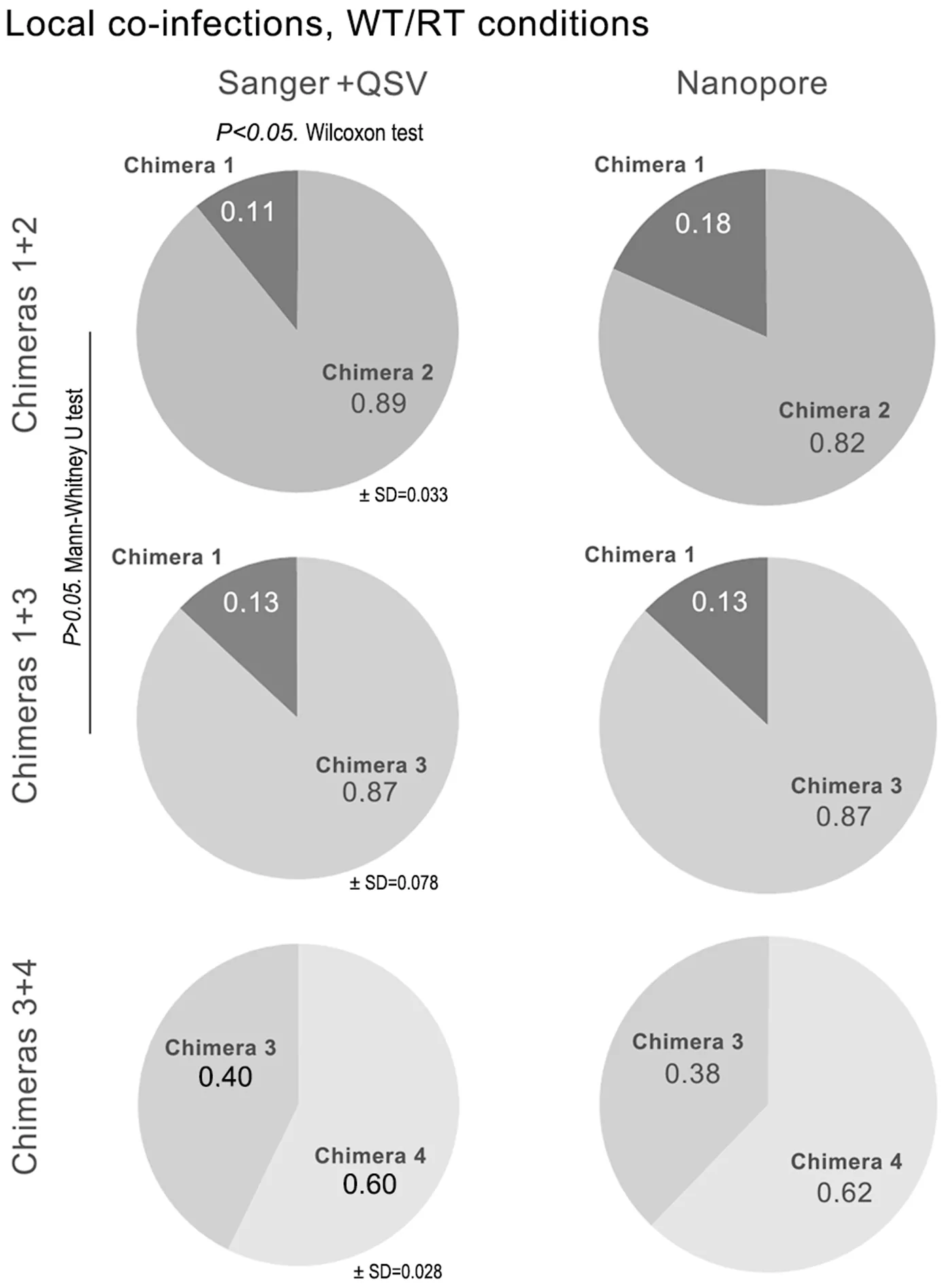
Ratios in local co-infections of chimera variants that differ in one nucleotide. Pie charts show co-infection ratios of chimeras 1 + 2; 1 + 3; and 3 + 4 in infiltrated tissues at 5 days post infiltration, in wild-type *Nicotiana benthamiana* plants, at 23 °C (WT/RT conditions). The genomes of the chimera pairs differ only in one nucleotide, which translates in a single amino acid difference in HCPro (see Fig 1). Ratios were determined either by electropherogram analysis of polymorphic nucleotide sites in Sanger-sequenced RT-PCR *HCPro* fragments (panels to the left), or by nanopore sequencing of double-stranded *HCPro* cDNAs (panels to the right). Data for chimeras 1 + 2 and 1 + 3 were each obtained from five independent biological replicates (agroinfiltrated discs) n = 5. Data for chimeras 3 + 4 represent the combined results of two independent experiments, yielding a total of n = 11. Nanopore sequencing data were generated from pooled ds cDNA samples prepared from the same biological replicates used for Sanger sequencing. Statistical significance of the proportion of the two coinfecting variants within discs was assessed using paired two-tailed Wilcoxon signed-rank test. Differences in the proportion of chimera 1 in 1 + 2 nd chimera 1 in 1 + 3 co-infections were evaluated using unpaired two tailed Mann-Whitney U test.

Relative ratios in the local co-infections shown in Fig 2 of variants with single nucleotide differences [chimeras 1 + 2 (amino acid 352, Ile vs. Thr); 1 + 3 (amino acid 454, Leu vs. Arg); 3 + 4 (amino acid 352, Ile vs. Thr, in a backbone of amino acid 454 Arg) were determined under standard WT/RT conditions, using both, Sanger and nanopore sequencing (Fig 3, left and right pie charts, respectively). Relative proportions obtained with the two techniques were broadly comparable. In the three co-infections, differences between the co-infecting chimeras were significant (*P < 0.05.* Wilcoxon test), with those chimeras with lower titers in single infections being the ones with lower proportions in co-infections (Figs 2A vs 3). The proportion of chimera 1 in co-infections with either chimera 2 or with chimera 3 (Fig 3, upper and middle pie charts to the left, respectively) was statistically similar (*P > 0.05.* Mann-Whitney U test).

**Fig 4:**
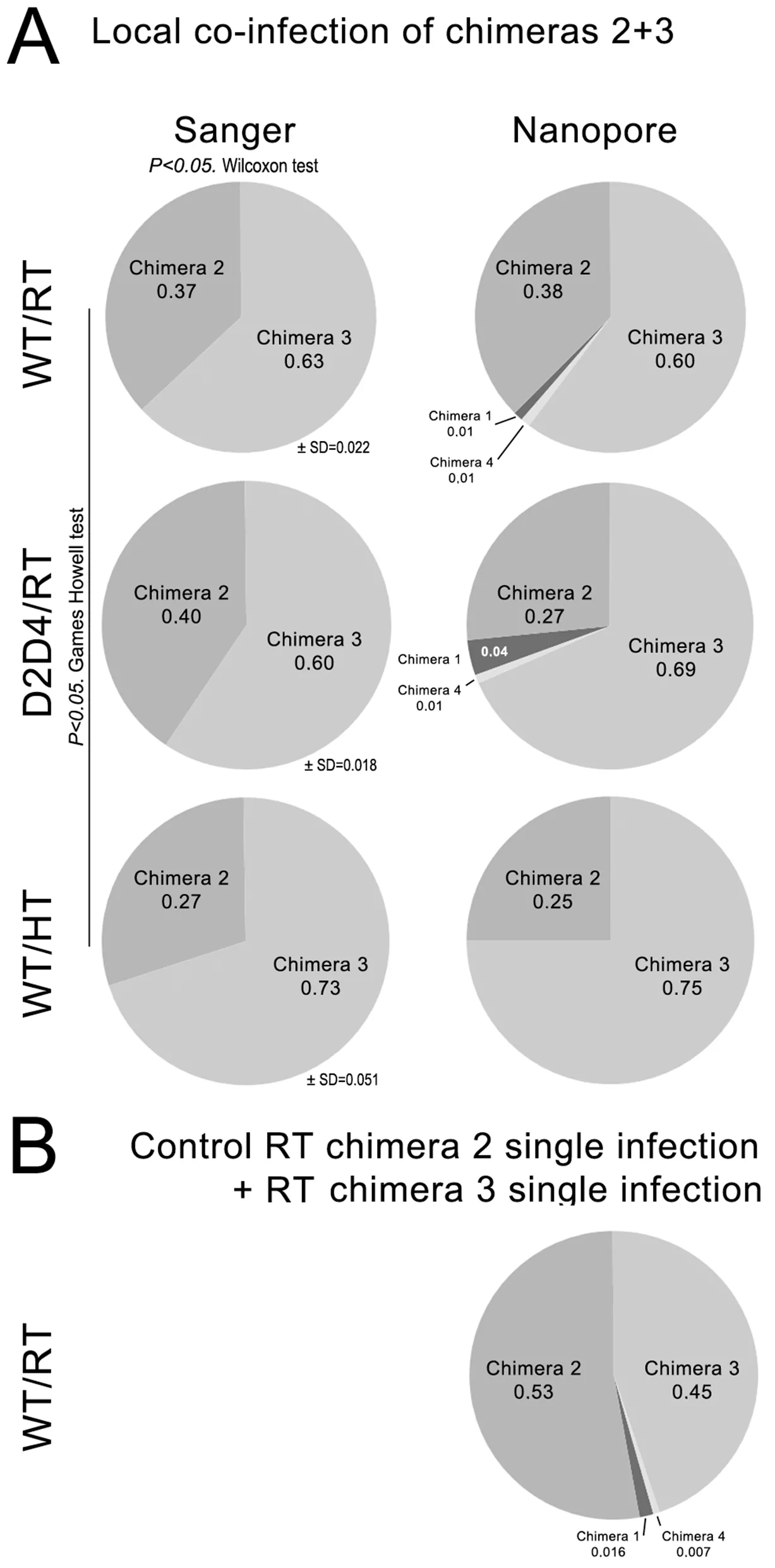
Ratios in local mixed infections of two chimera variants that carry each a different beneficial mutation in *HCPro.* (A) Pie charts show co-infection ratios of chimeras 2 + 3 in infiltrated tissues at 5 days post infiltration, kept under three environments: WT/RT, wild-type *Nicotiana benthamiana* plants kept at 23 °C (room temperature); WT/HT, in which temperature was increased to 29 °C 24 hours after infiltration (high temperature, HT); D2D4/RT, in which silencing-compromised *N. benthamiana* were used instead of WT plants, kept at RT. Ratios were determined either by electropherogram analysis of polymorphic nucleotide sites in Sanger-sequences of RT-PCR *HCPro* fragments (panels to the left), or by nanopore sequencing of double-stranded *HCPro* cDNAs (panels to the right). Sanger sequencing data represent the combined results of two independent experiments, comprising a total of biological replicates (n) of 10 for WT/RT; 9 for D2D4/RT; and 9 for WT/HT. (B) Pie chart of a nanopore sequencing control from ds cDNAs generated from two combined single-stranded cDNAs obtained by reverse transcription (RT) from either single chimera 2 or single chimera 3 infections, under WT/RT conditions. Nanopore sequencing results (pie charts to the right) were generated from pooled ds cDNA samples prepared from the same biological replicates used for Sanger sequencing. Statistical significance of the proportion of the two coinfecting variants within discs was assessed using paired two-tailed Wilcoxon signed-rank test. Differences in the proportion of chimera 3 or 2 among the three experimental conditions (WT/RT, D2D4/RT, and WT/HT) were evaluated using one-way analysis of variance ANOVA for independent groups, followed by unpaired two-tailed Games–Howell post hoc tests to account for unequal variances among groups.

In the same way, we determined relative ratios in local co-infections of chimeras 2 + 3 under WT/RT, D2D4/RT, and WT/HT conditions. Data from Sanger analysis of the triplet encoding amino acid 352 Thr (A**C**T) vs. Ile (A**T**T), encoded by chimeras 2 and 3, respectively, are shown in Fig 4A (pie charts to the left). They showed a significant local prevalence of chimera 3 over chimera 2. Ratios obtained from nanopore sequencing vs. Sanger sequencing were very similar in WT/RT and WT/HT conditions (Fig 4A, pie charts to the right vs. charts to the left).

Interestingly, environmental conditions (D2D4/RT and WT/HT) significantly affected the relative proportions of chimeras 2 and 3 comparing with WT/RT, high temperature resulted in an increased prevalence of chimera 3 and a decrease of ratio of chimera 2 (Fig 4A, lower vs. upper pie charts on the left; *P < 0.05*. Games Howell test). Similarly, the relative proportions of chimeras 2 and 3 differed significantly between D2D4 and WT plants (Fig 4A, middle vs. upper pie charts on the left; *P < 0.05*. Games–Howell test). In D2D4/RT conditions, ratios obtained from the two methods were more different than in the other two conditions, but both showed nevertheless a prevalence of chimera 3 over chimera 2 (Fig 4A, central pie charts).

The analysis of nanopore Q20 reads found some homologous recombinants in local co-infections of chimeras 2 and 3 (which would generate chimeras 1 or 4). In WT/RT and WT/HT they were less than 1% of reads. In D2D4/RT conditions, 4% of the reads were of chimera 1 (352 Ile and 454 Leu) (Fig 4A). However, control analysis of ds cDNAs obtained from single-stranded (ss) cDNAs obtained by reverse transcription from total RNAs of single chimera 2 or chimera 3 infections also showed the presence of homologous recombinant reads, at frequencies of 1.6% for chimera 1 and 0.67% for chimera 4 (Fig 4B). Thus, we arbitrarily set a minimum threshold of 3%, as possible indication of actual presence of homologous recombinants in local co-infections.

In WT/RT conditions, the chimera 3/chimera 2 ratio (0.63/0.37 Sanger; pie chart to the left in Fig 4A) indicates that chimera 3 accumulates ~ 1.7x times more than chimera 2, and has thus, a higher fitness in local tissues.

Indeed, when analyzing differences in fitness between co-infecting chimeras 2 + 3, following [35]:


$$\begin{array}{c} n(ORBt\text{5}/ORAt\text{5})-ln(ORBt\text{0}/ORAt\text{0}) \end{array}$$ (1)

$$\begin{array}{cc} & Fitness\;difference\;=\;logarithm\;(observed\;ratio\;of\;variant\;B/observed\;ratio\;of\;variant\;A,\;both\;at\;time\;\\ & \text{5}\;dpi)\;-\;logarithm\;(observed\;ratios\;of\;variant\;B/observed\;ratio\;of\;variant\;A,\;both\;being\;\\ & equal\;at\;their\;start\;from\;T-DNA\;expression\;after\;infiltration). \end{array}$$

Chimera 3 showed higher fitness than chimera 2 under the three environments assessed, in particular under elevated temperature (Table 1).

**Table 1:** Fitness difference between chimera variants 2 and 3 in local co-infections at 5 days post-inoculation (dpi), under three different environments, estimated from their observed Sanger sequencing ratios (shown in Fig 4).

Co-infecting chimeras 2 + 3	WT/RT	D2D4/RT	HT/RT
***ln(ORBt5/ORAt5)−ln(ORBt0/ORAt0)* (Equation 1)** [35]	Ln(0.63/0.37)-ln(0.5/0.5) = 0.53	Ln(0.60/0.40)-ln(0.5/0.5) = 0.40	Ln(0.73/0.27)-ln(0.5/0.5) = 0.99

ORAt5: observed ratio of variant A (chimera 2) at 5 dpi.

ORBt5: observed ratio of variant B (chimera 3) at 5 dpi.

ORAt0 = ORBt0: initial ratios of variants A and B at the start of T-DNA expression (both equal, 0.5 each).

### Visualization of local co-infections

**Fig 5:**
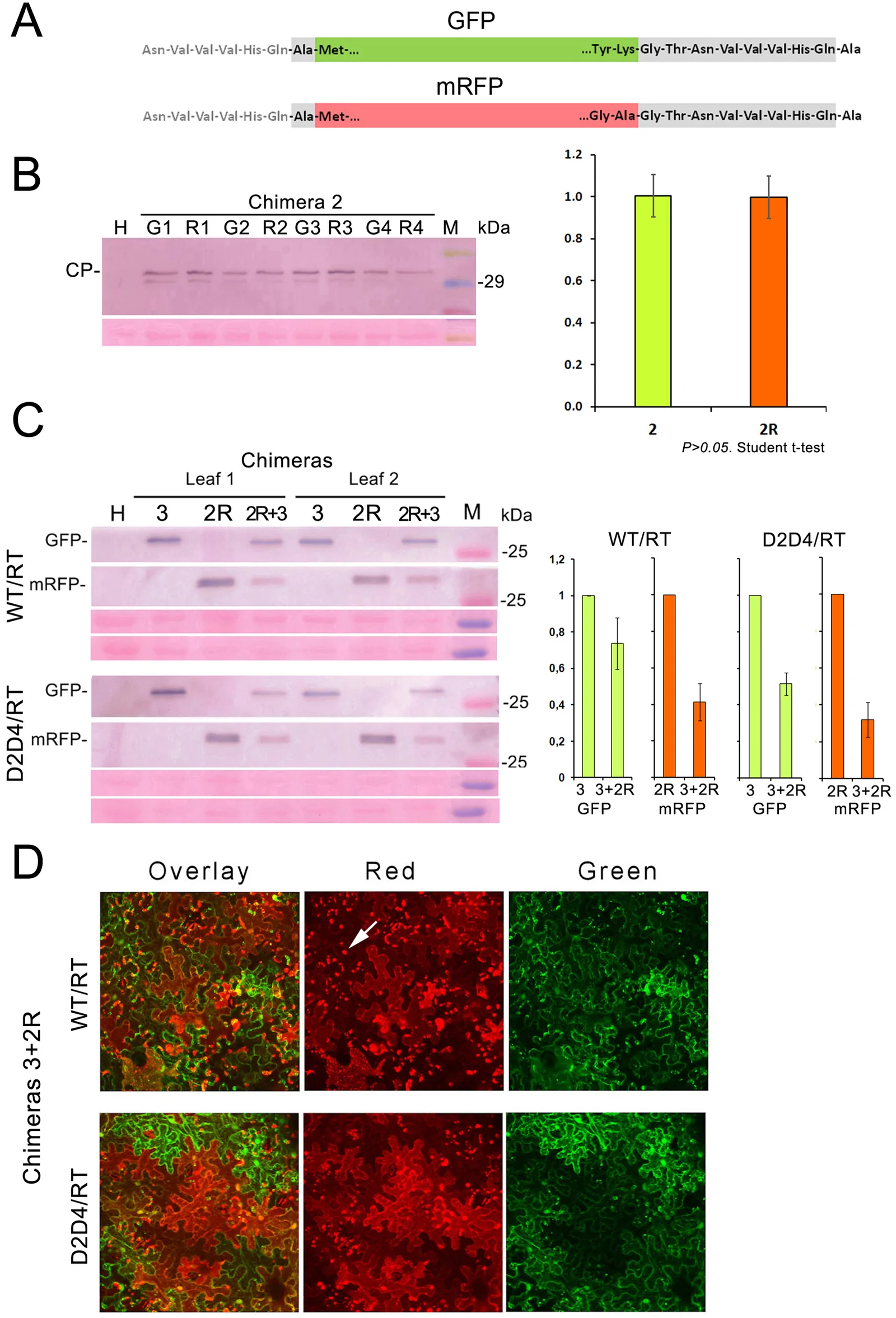
Visualization *in vivo* of co-infecting chimeras 2 and 3 in *Nicotiana benthamiana* epidermal cells. To visualize co-infection of the two chimeras, the *GFP* cistron in chimera 2 was replaced with an *mRFP* one. (A) Schematic representation of the *GFP* cistron present in the original pLX-PPV construct [40] and in chimera 2 [31], and of the *mRFP* cistron that replaced it, creating chimera 2R. The encoded mRFP has the same extra amino acids fused to its termini than the GFP has (highlighted in grey). (B) Western blot detection and quantification of the local titers of chimeras 2 vs. 2R at 5 days post infiltration (dpi) in WT/RT show that their titers were similar. G1-R1, G2-R2, G3-R3, and G4-R4 pairs lanes correspond to agrotaches expressing either chimera 2 (green) or 2R (red) in opposite sides of the same leaf. (C) Detection in local tissues of single or mixed infections of chimeras 2R and 3, at 5 dpi, in either WT/RT or D2D4/RT conditions: GFP and mRFP markers encoded by the variants were detected by western blot, and the blots detecting each marker are shown. Ponceau S-stained controls (rubisco band) appear below. GFP and mRFP levels were quantified relative to their average values in single infections (arbitrary value of 1) in the charts to the right. Drops in the levels of GFP in single infection vs. co-infection are significantly lower than the drops in the levels of mRFP in single vs. co-infection. *P < 0.05*, Student t-test. (D) GFP and mRFP fluorescence were visualized in epidermal cell fields simultaneously *in vivo* using confocal microscopy (green and red panels, respectively), and combined to create the overlay panels to the left. The arrow in the upper red panel illustrates an mRFP inclusion.

To visualize *in vivo* the local co-infection of chimeras 2 and 3, we substituted an *mRFP* cistron for the *GFP* cistron in chimera 2, creating chimera 2R. Both markers are expressed with the same extra amino acids fused at their N- and C-termini (Fig 5A). In WT/RT conditions, the local titers of chimera 2 and of chimera 2R were similar (Fig 5B). Therefore, we co-infected chimera 2R and chimera 3 in WT/RT and D2D4/RT conditions (viral levels in WT/HT conditions were too low for efficient observation of fluorescence).

Both, GFP and mRFP were detected serologically in total protein extracts from co-infected tissues. Considering that chimeras 2 and 2R accumulated to similar levels (Fig 5B), the relative drops of GFP and mRFP in single infections vs. co-infections (Fig 5C, western blots) were consistent with the variant ratios observed in co-infections of chimeras 2 and 3 shown in Fig 4A.

GFP- and mRFP-derived fluorescence were both observed in most epidermal cells of agroinfiltrated tissues (Fig 5D, confocal panels to the left). Most of the GFP distributed uniformly throughout the cell cytoplasm and nucleus (bar the nucleolus), and mRFP followed the same distribution, but accumulated more in inclusions located towards the cell periphery (Fig 5D, arrow) and their abundance varied among cells. However, in any epidermal cell field, it was found that overlay colors displayed a green-yellow-orange-red range, depending on the individual epidermal cell.

### Assessment of variant prevalence in systemic tissues, after local co-infection

**Fig 6:**
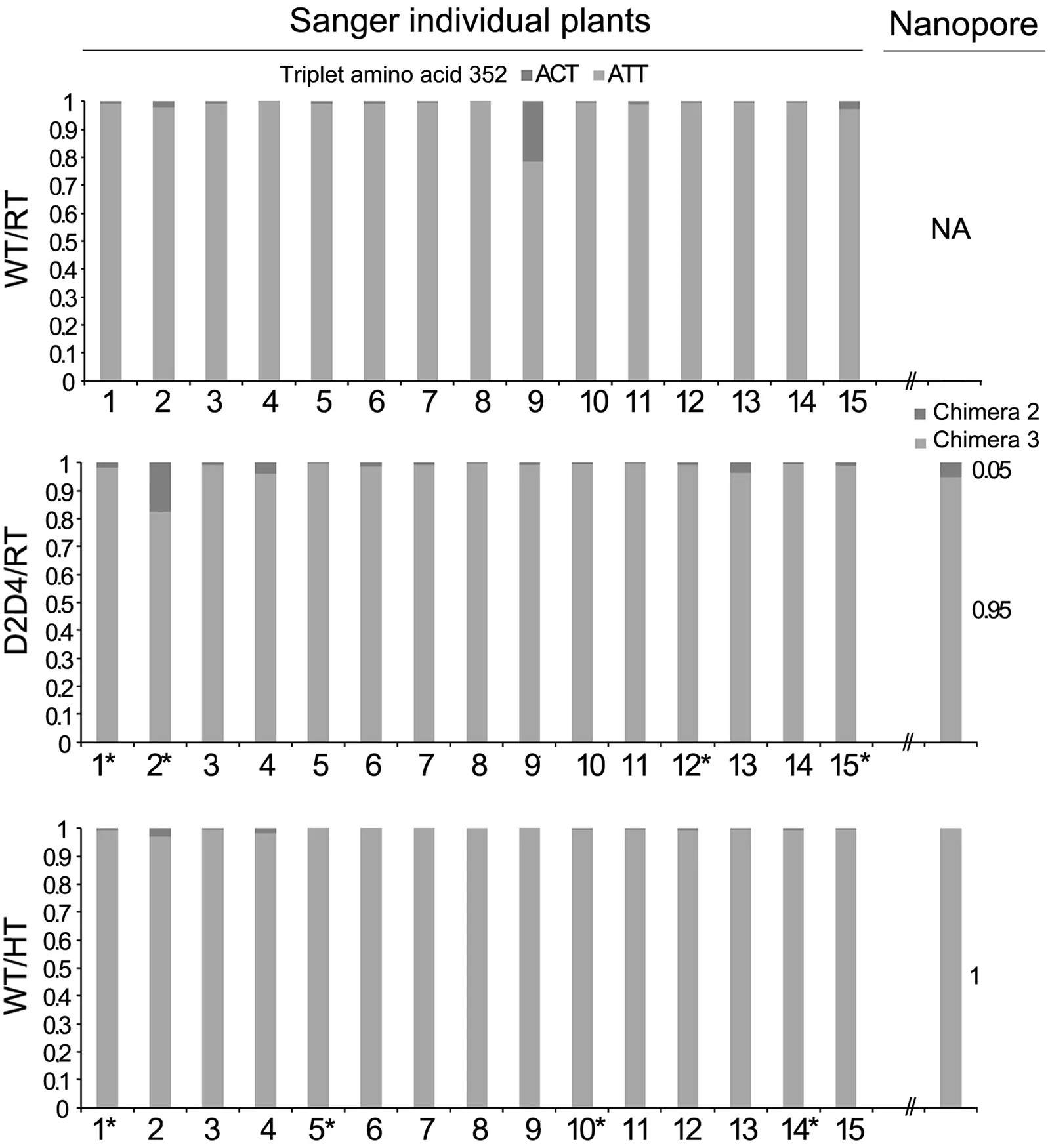
Variant prevalence in the systemic tissues of individual plants co-inoculated with two chimeric variants that each carry a different beneficial mutation in the *HCPro* cistron. Charts show co-infection ratios in systemic tissues, 29 days after they had been co-infiltrated locally with chimeras 2 + 3. Plants were kept under three environments: WT/RT, wild-type *Nicotiana benthamiana* plants kept at 23 °C (room temperature); WT/HT, in which temperature was increased to 29 °C, 24 hours after infiltration (high temperature, HT); D2D4/RT, in which silencing-compromised *N. benthamiana* were used instead of WT plants, kept at RT. Prevalence was determined by electropherogram analysis of polymorphic nucleotide sites in Sanger-sequences of RT-PCR *HCPro* fragments from 15 individual plants (bars to the left of the chart). Nanopore sequencing of double-stranded *HCPro* cDNAs (bar to the right), obtained from total RNAs combined from 4 of the 15 plants, which are indicated with an asterisk is also shown in the panels to the left. NA, not available.

We then assessed viral populations in systemic tissues of 45 individual plants, 29 days after their co-infiltration in local patches with chimeras 2 + 3, under either WT/RT, D2D4/RT, or WT/HT environments (15 plants/case). Using Sanger analysis of triplets encoding amino acids 352 and 454, we found that in all the 45 plants, chimera 3 prevailed overwhelmingly in the three environments, in particular, at high temperature (Fig 6). Only in two plants out of the 45 were amino acid 352 (A**C**T Thr/ A**T**T Ile) ratios ~0.2/0.8 (Fig 6, plant 9 in WT/RT, and 2 in D2D4/RT conditions).

Nanopore reads were obtained from combined total RNA pools of four of the 15 plants that were analyzed by Sanger under D2D4/RT conditions, or WT/HT conditions (Fig 6, plants indicated with asterisk). Under D2D4/RT conditions, 5% of the reads corresponded to chimera 2 and 95% to chimera 3. Under WT/HT conditions, all nanopore reads were exclusively of chimera 3 (Fig 6, last bars to the right of each chart).

Viral populations were also analyzed in systemic tissues of 16 individual plants, 29 days after co-infiltration with chimeras 3 and 4 under either WT/RT or D2D4/RT conditions (8 plants/case). In all 16 plants both variants were present in systemic tissues, but chimera 4 consistently predominated over chimera 3 in both environment conditions. Only one D2D4 plant (Plant 3) displayed a higher proportion of chimera 3. On average chimera 3/chimera 4 ratios were ~0.2/0.8, and no significant difference in chimera ratios was observed between WT and D2D4 plants (S2 Fig).

### Detection of new mutations in the *HCPro* cistron of variants infecting systemic tissues, and their properties

**Fig 7:**
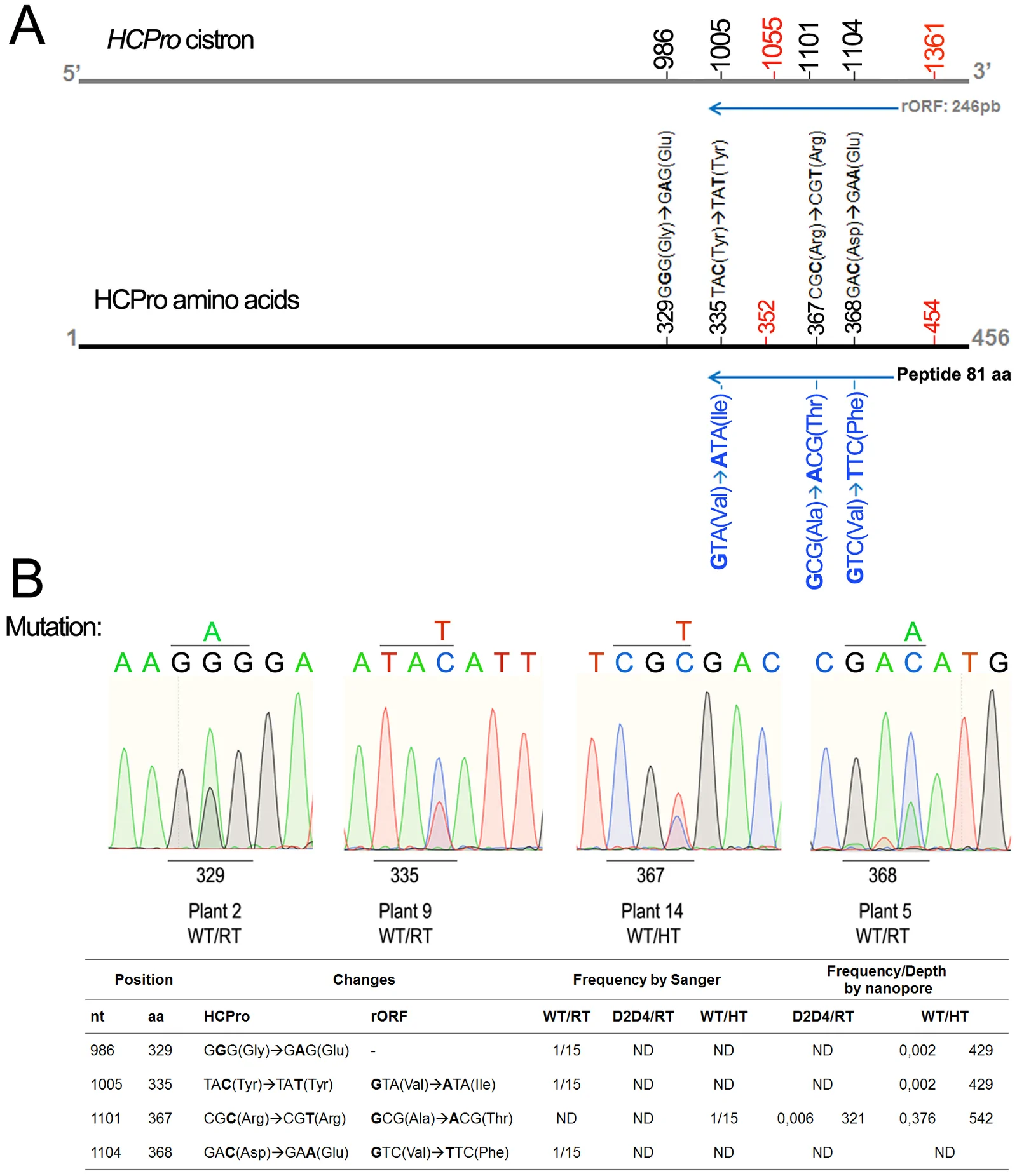
Novel HCPro mutations identified in systemic tissues of individual plants. (A) Schematic representation of four new mutations that were detected by Sanger sequencing of RT-PCR fragments of the *HCPro* citron 3´-region, encoding ~40% of HCPro (amino acids 269–456), amplified from systemic tissues of 45 individual plants, 29 days after co-infiltration with chimeras 2 + 3, and kept under three different environment conditions (15 plants/condition, indicated in Fig 6). For each mutation, the nucleotide triplet position is indicated, and mutated nucleotide sites are highlighted bold. Their effects on amino acids encoded by the *HCPro* cistron and by a putative reverse reading frame (rORF) are indicated. (B) Electropherograms from the polymorphic sites for each of the four mutations amplified from individual plant which the four mutations were detected. Sanger and nanopore sequencing data, the latter from a 4-plant pool (indicated with an * in Fig 6) are summarized below. ND, not detected.

Sanger analysis of the 3´-third of the *HCPro* cistron in viral populations infecting each of the 45 plants presented in Fig 6 showed that in some particular plants, four new mutations had separately incorporated in the prevalent chimera 3 variant, becoming two of the mutations relatively abundant, and the other two, prevalent in the corresponding plants. In one plant, a mutation in the triplet encoding HCPro amino acid 329, which led to non-conservative substitution GGG (Gly)→G**A**G (Glu), became prevalent in the viral population infecting it (Fig 7, WT/RT plant 2). In another plant, a conservative substitution occurred at amino acid 368 GAC (Asp)→G**AA** (Glu) (Fig 7, WT/RT plant 5). The other two mutations were silent in the *HCPro* cistron, but both would have induced amino acid substitutions in a putative peptide encoded by a putative small reading frame in the complementary RNA, if it was ever expressed: one was in the triplet encoding amino acid 335 Tyr, which would cause a Val → Ile substitution in the putative reverse peptide (Fig 7, WT/RT plant 9). The other one, in the triplet encoding amino acid 367 Arg, which causes an Ala → Thr substitution in the putative reverse peptide, became prevalent in one plant kept at high temperature (Fig 7A, WT/HT plant 14), and could be a candidate temperature-associated mutation requiring functional validation. By contrast, no new mutations were detected by Sanger sequencing of the systemic co-infection of chimeras 3 and 4 shown in (S2 Fig).

Nanopore data from the 4-plant RNA pools were consistent with the Sanger data (Fig 7B). Nanopore analysis detected additional low frequency (3–6% of reads) mutations, neither abundant nor prevalent: six and ten in D2D4/RT and WT/HT 4-plant pools, respectively (S1 Table). As comparison, nanopore reads in the local single infection of chimeras 2 and 3 combined control at WT/RT at 5 dpi, presented in Fig 3B showed five such low-frequency mutations (S1 Table).

**Fig 8:**
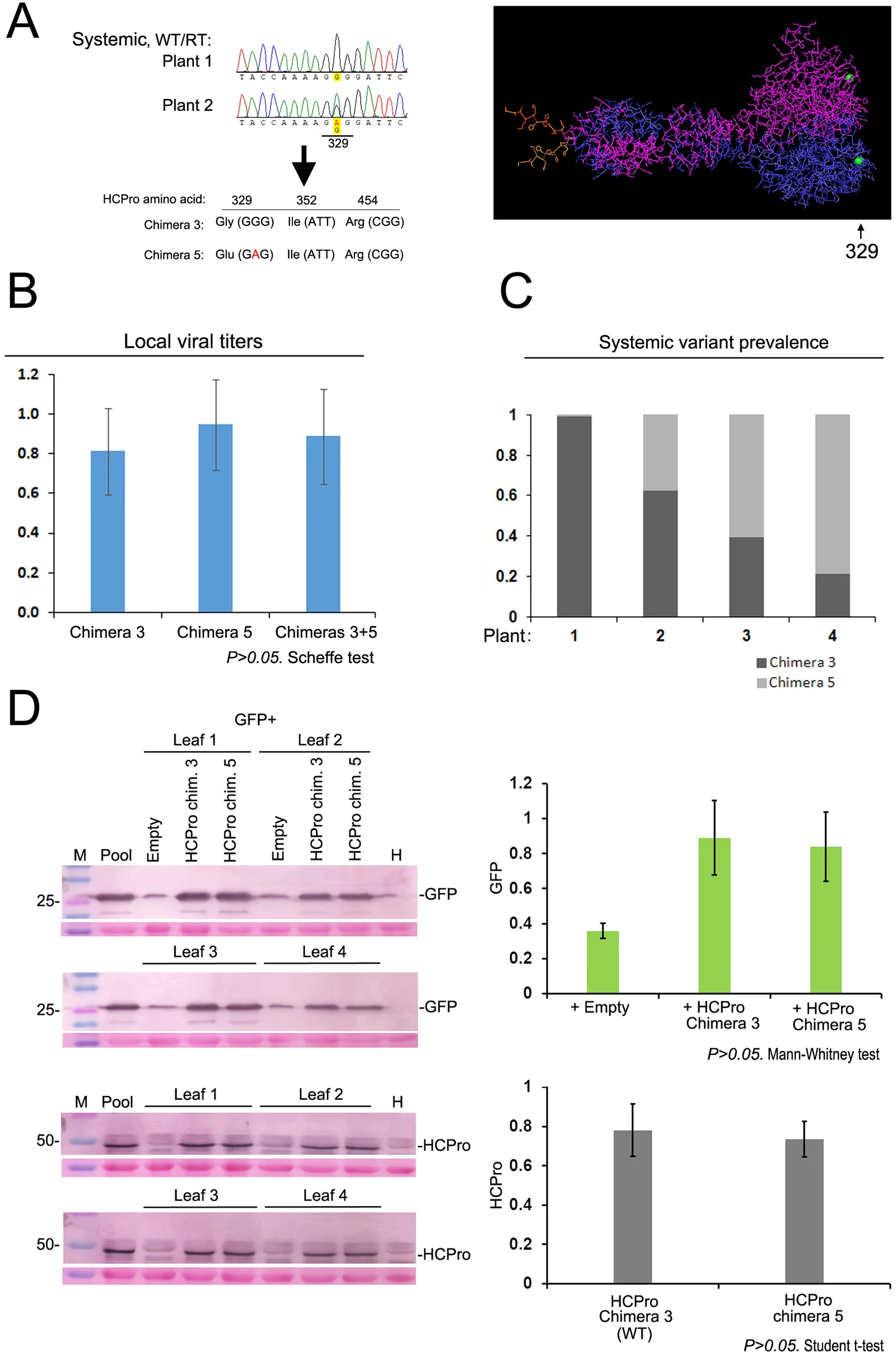
Characterization of the effect of a novel naturally-acquired substitution at HCPro amino acid 329. (A) In the systemic tissues of one plant (Fig 7B, WT/RT plant 2) a novel natural non-conservative substitution at HCPro position 329 had become prevalent (Gly, GGG vs. Glu, GAG, electropherogram), in a chimera 3 backbone. This mutation was incorporated into chimera 3 by reverse genetics, creating chimera 5. The position of this amino acid in HCPro is indicated in the panel to the right. (B) Relative titers of chimeras 3 and of chimera 5, in local single or mixed infections at 5 dpi, were found to be comparable. The chart shows the combined data from three independents experiments. The number of replicates (n) is: chimera 3, 10; chimera 5, 10, chimeras 3 + 5, 12. (C) 14 dpi after initial local infections with chimeras 3 + 5, the proportion of each chimera variant in systemic tissues varied with each individual plant. (D) The silencing suppression effects of the HCPro encoded by chimera 3 or by chimera 5 on a *GFP* reporter when assessed in agropatch assays were comparable (GFP western blots and chart to the right). Transient levels of both HCPro variants were also similar (HCPro western blots and chart to the right). Statistical tests and their significance at *p < 0.05* are indicated below the chart.

To assess the effect on the virus of the non-conservative, prevalent mutation at amino acid 329 Gly → Glu found in the HCPro of chimera 3 infecting WT/RT plant 2 (Fig 7), we introduced it into the chimera 3 clone, creating chimera 5 (Fig 8A), and also into a binary vector expressing the *P1-6x-HCPro* bi-cistron, to analyze its properties in WT/RT conditions. Relative to chimera 3 titers, the mutation in chimera 5 did not alter local infection titers (Fig 8B). From local chimera 3 + 5 co-infections, the proportion of each variant in systemic tissues at 14 dpi depended on the individual plant (Fig 8C), and suggests that neither variant had a definite advantage to prevail over the other. We found that in virus-free, silencing suppression agropatch assays, both HCPro variants suppressed the silencing of a reporter comparably, and the two accumulated transiently to similar levels, suggesting that mutation 329 did not affect the silencing suppressor function, and/or the suppressor stability/maturation (Fig 8D). We could thus not show any effect, either positive or negative, of mutation 329 on prevalence or on function, with our available functional assays.

## Discussion

We investigated how several clonal chimera variants that in their entire genomes only have either one or two naturally-acquired nucleotide differences in their *HCPro*, with different effects on virulence (chimeras 1, 2, 3, 4; [32]), would co-exist, influence each other, and prevail one over the other. To do that, we co-expressed them equally in pairs under our tested local agroinfiltration conditions, and studied first their local titers in co-infection vs. single infection, in the absence of viral movement. We found that co-infection titers showed no evidence of synergism, either from a variant with a beneficial mutation, relative to the variant lacking it (1 + 2; 1 + 3; 3 + 4), or between chimeras 2 and 3, which each harbor each a different beneficial mutation that affects different HCPro functions (Fig 2). The latter happened in spite of their benefits being additive in the same virus (chimera 4). One of the mutations, at amino acid 454, enhances HCPro autoproteolytic cleavage [32], and it was unsurprising that it did not help the other variant in trans, since the protease activity is cis-acting [36,37]. However, the beneficial mutation at amino acid 352, which enhances suppression of the silencing of a transient, cytoplasmic *GFP* mRNA in agropatch assays, did not help the other variant either in trans, despite HCPro being cytoplasmic. This suggests that subcellular constrains limit its antiviral silencing activity. In this regard, because of the preference by HCPro to bind vsRNAs over those of plant sequence, or those derived from transient T-DNA transcripts (even though it suppresses the silencing of T-DNA-expressed reporters in agropatch assays), it had already been suggested that, with regard to antiviral silencing suppression, HCPro targeting and effects have subcellular constrains [25]. Alternatively, the effects in trans of each variant HCPro could neutralize each other. However, the proportion of chimera 1 in co-infections with either chimera 2 or with chimera 3 (Fig 3, upper and middle pie charts to the left, respectively) was statistically similar (*P > 0.05.* Mann-Whitney U test), suggesting that any trans effect of mutation at position 352 (Ile → Thr) would be similar to that at position 454 (Leu → Arg), which we know the latter operates only in cis.

We determined variant ratios in local co-infections by two separate means (Figs 3 and 4). In all cases, observed ratios were consistent with variants not affecting the accumulation of each other in the co-infected cell.

If under our equal agroinfiltration densities (in our experiments, OD 600 of 0.3 for each type of bacteria, either in single- or in co-infiltrations) we assume that variants do not influence each other in co-infected cells, then local variant ratios in co-infections could theoretically be deducted from the observed titers of each variant in single local infections, shown in Fig 2A. In this case:


$$\begin{array}{c} ERA\;=\;OTA/(OTA+OTB) \end{array}$$ (2)

$$\begin{array}{cc} & Expected\;ratio\;of\;variant\;A\;=\;observed\;titer\;from\;western\;of\;variant\;A\;in\;single\;infection/\;\\ & (observed\;titer\;from\;western\;of\;variant\;A\;+\;observed\;titer\;from\;western\;of\;variant\;\\ & B,\;both\;in\;single\;infections) \end{array}$$

The ratios deduced from this formula are shown in Table 2, and are consistent with the ratios observed in all cases, with the exception of co-infection of chimeras 2 + 3 under D2D4/RT conditions. Under this latter condition, the chimera 2/3 ratios expected from their titers in single infections, shown in Fig 2B, would be similar (highlighted in red, Table 2), but actual observed chimera 2 ratios were significantly lower than those of chimera 3 (Fig 4A). This may be related to the observation that HCPro with 352 Thr accumulates comparatively less than HCPro 352 Ile in this type of plant, whereas in WT plants both accumulate the same, as reported by [32]. One major difference between the two plant backgrounds may lie in the populations of 21- and 22-nt sRNAs generated in response to viral infection or to transcripts derived from agroinfiltrated T-DNA [38]. PVY HCPro is known to bind 21–22 nt sRNAs, through mechanisms that remain unclear, as part of its silencing suppression activity [24]. This sensitivity of HCPro to the sRNA environment suggests that interactions with sRNAs are important during infection or may contribute to stabilization of the protein. Consequently, the effect of the transgenic background on mixed infection may result from the balance between a positive effect associated with reduced antiviral silencing defenses and a negative effect caused by the decreased accumulation of specific suppressor variants. However, this does not exclude the possibility that in transgenic plants, the lack of interference between variants infecting the plant may be affected.

**Table 2:** Observed vs. expected local co-infection ratios at 5 days post-inoculation, assuming that variants do not influence each other in co-infected cells.

	WT/RT				D2D4/RT	WT/HT
Co-infecting chimeras:	1 + 2	1 + 3	3 + 4	2 + 3	2 + 3	2 + 3
Observed ratios Sanger (Figs 3 and 4A)	0.11/0.89	0.13/0.87	0.40/0.60	0.37/0.63	0.40/0.60	0.27/0.73
Observed ratios Nanopore (Figs 3 and 4A)	0.18/0.82	0.13/0.87	0.38/0.62	0.38/0.60	0.27/0.69	0.25/0.75
Expected ratios *ERA = OTA/(OTA + OTB)* (Equation 2) ERA: expected ratio of variant A in co-infection A + B OTA: observed titer of variant A in single infection OTB: observed titer of variant B in single infection						
Expected ratios from observed titers (western blot)	0.08/0.92	0.07/0.93	0.40/0.60	0.41/0.59	0.50/0.50	0.23/0.77

If under equal agroinfiltration densities we assume that variants do not influence each other in co-infected cells, then the co-infection titers measured are compatible with the variant ratios observed by sequencing, and the titers obtained in single infections:


$$\begin{array}{c} ET(A+B)\;=\;(ORA\;\times \;OTA)\;+\;(ORB\;\times \;OTB) \end{array}$$ (3)

$$\begin{array}{cc} & Expected\;titers\;from\;variant\;A+B\;co-infection\;=\;(observed\;ratio\;of\;A\;in\;double\;infection\;from\;sequencing\;\\ & \times \;observed\;titer\;of\;A\;in\;single\;infection\;from\;western)\;+\;(observed\;ratio\;of\;B\;in\;double\;infection\;from\;sequencing\;\\ & \times \;observed\;titer\;of\;B\;in\;single\;infection\;from\;western) \end{array}$$

Where the observed titer was determined from the single infection titers measured by western blot (Fig 2A) and the observed ratio was the relative proportion of each variant in co-infected agropatches obtained by sequencing (Figs 3 and 4A). And indeed, the observed co-infection titers shown in Fig 2B correlated well with those deduced with this formula from the observed ratios in co-infections shown in Figs 3 and 4A, and the observed titers of each variant in single infections shown in Figs 2 and 3, and they are indicated in Table 3. In co-infections that included chimera 1, expected titers were slightly higher than the observed ones (Table 3), but this could be because of the relative under-detection of chimera 1 titers in western blots, as they are comparatively very low, relative to the other variants, nearer the lower limit of detection of the antibody, thus possibly affecting the linearity of their relative detection.

**Table 3:** Observed vs. expected local co-infection titers at 5 days post-inoculation, assuming that variants do not influence each other in co-infected cells.

	WT/RT				D2D4/RT	WT/HT
Co-infecting chimeras:	1 + 2	1 + 3	3 + 4	2 + 3	2 + 3	2 + 3
Observed co-infection titers (Fig 2)	0.45	0.62	0.93	0.60	0.91	0.04
Expected co-infection titers: *ETA + B= (ORA × OTA) + (ORB × OTB)* (Equation 3) ETA + B: expected titer in mixed infection of A + B ORA: observed ratio of variant A in mixed infection of A + B OTA: observed titer of variant A in single infection ORB: observed ratio of B in mixed infection of A + B OTB: observed titer of variant B in single infection						
Expected co-infection titers from observed Sanger ratios	0.64	0.75	0.99	0.67	0.96	0.07
Expected co-infection titers from observed Nanopore ratios	0.60	0.75	1.00	0.66	0.92	0.07

We visualized local co-infection of chimeras 2R + 3 that express mRFP and GFP, respectively under WT/RT conditions (Fig 5). Although we observed variability among epidermal cells on GFP- or mRFP-derived fluorescence, it must be taken into consideration that while GFP appeared mostly soluble, mRFP tended to accumulate numerous cytoplasmic inclusions of different number and sizes, depending on the cell. Because excitation by confocal lasers of the fluorophore in soluble proteins, and consequent emission of light would be more efficient than for proteins clustered in inclusions, the visual assessment of relative protein content in individual cells may be biased by the different number of mRFP inclusions that each cell has.

On the other hand, the western blot data in Fig 5C assess GFP and mRFP levels in total proteins extracted from the infiltrated leaf in single infections by chimera 3 vs. co-infections by chimeras 3 + 2R. And likewise, of mRFP in single infections by chimera 2R vs. co-infection by chimeras 3 + 2R. In co-infections, both GFP and mRFP levels decreased relative to single infections, the decrease was less marked in the case of GFP (chimera 3), and more so in the case of mRFP (chimera 2R). Since chimeras 2 and 2R accumulate equally (Fig 5B), this is consistent with what would be expected from the expected genomic analysis of local co-infection ratios of chimeras 2 and 3 (Fig 4A, upper and middle pie charts).

Taken together, our data support a hypothetical co-infection model in which equal amounts of agroinfiltration-delivered T-DNAs express similar amounts of mRNAs that once in the cell cytoplasm translate into viral proteins, initiating viral amplification. The initial viral translation products quickly hoard scarce materials and sites available for replication/translation, limiting competition among replication complexes and thus, fixing early the proportion of replication complexes for each virus variant (equal on average in the infiltrated patch but perhaps not necessarily in the individual cell), and that proportion will not change through time, independent of the different accumulation rates of each variant. Each variant contributes to the total viral population according to its relative proportion. Each variant replication appears independent from the other one (Tables 2 and 3). We find no evidence of trans-complementation. Very few replication interactions seem to occur, as suggests the very low presence of homologous recombinants. Variants do not appear to influence each other through the functions of their cytoplasmic HCPros either, further extending their isolation from each other. Each variant follows an essentially autonomous replication trajectory within the infected cell.

While co-infecting variants do not seem to influence each other, environment parameters by contrast could alter not only variant co-infection titers (Fig 2), but also their ratios, because their differential effects on functions affected by the specific HCPro mutation that each variant has, affects their relative accumulation rates. This is shown for chimeras 2/3 in WT plants under HT vs. RT conditions, and in D2D4 vs. WT plants, at RT (Fig 4A).

When we looked at the systemic infection outcomes in chimeras 2 + 3 co-infections we found that chimera 3 prevailed in all 45 plants, under different environments. Only in two plants, kept at RT, exclusion of chimera 2 was not yet fully achieved at 29 dpi (~17–20% of the viral population; Fig 6). It appears then that a faster proteolytic cleavage by HCPro provides a clear advantage to the systemic dispersion of a variant (chimera 3) than a higher suppression of silencing (chimera 2), once the virus is in the cell. And this results in the rapid systemic prevalence of the former. Nevertheless, the higher suppression provided by the 352 mutation does have an additive positive effect on the relative viral fitness in the systemic infection of the variant that carries it: in systemic chimera 3 + 4 coinfection, all 16 plants retained detectable/substantial levels of chimera 3 (Figs 6 vs S2).

Interestingly, we found four additional mutations in the *HCPro* cistron that separately incorporated in the systemic chimera 3 variant in a sizeable or even prevalent part of the population (Fig 7). The mutants occurred in a minority of plants, and separately. This is rather similar to how mutations 352 and 454 appeared in the initial chimera [31]. The 329Gly → Glu substitution of chimera 5 represents a mutation that became prevalent in one plant. It however did not translate into detectable phenotypic changes in the experimental assays performed. Further, no significant differences were observed in local accumulation, systemic prevalence, or transient expression levels and silencing suppression activity on a reporter, relative to chimera 3 (Fig 8). However, these results do not exclude an impact of this mutation on other HCPro-related functions, or on the viral genome structure that were not assessed yet.

Interestingly, two of the new mutations were silent in the cistron, but not in a putative small reverse one. One of them might be a response to higher temperature (Fig 7, mutation 367), and would need testing. Examples of viral adaptation to thermal stresses through functional mutations are scarce (i.e., [39]).

Our results in conclusion show how nearly isogenic viral variants carrying distinct, adaptive mutations in a multifuctional protein, co-exist, compete among themselves, and adapt: we show that in local co-infections our variants do not influence each other with regard to titers and ratios. In contrast, biotic and abiotic environment factors can alter both, because of their differential effects on each variant HCPro functions. We also show that adaptation via new mutations in the *HCPro* cistron could proceed further through separate paths, in different plants.

## Materials and methods

### Plants and environment conditions

Two types of plants were used: WT *N. benthamiana* plants, and transgenic *N. benthamiana* plants whose *Dcl2* and *Dcl4* genes are partially silenced by a single hairpin-type transgene (transgenic line DCL2/4.5i; [38]). The transgenic line was kindly provided by Professor Kriton Kalantidis (University of Crete).

Three growth chamber-controlled environments were used to study viral infections: standard WT/RT conditions in which WT plants were kept at 23 °C (room temperature); conditions in which a biotic parameter was altered, using *Dcl2,4* silenced plants, (D2D4/RT conditions), and conditions in which an abiotic parameter, temperature, was elevated to 29 °C, 24 hours after infiltration of WT plants at 23 °C (WT/HT conditions). The three environments had a 16-hour light/8-hour dark photoperiod.

### Viruses and HCPro constructs

Four chimeras that differed in one or at most two nucleotides in their genomes were described previously: chimeras 1, 2, 3 and 4 ([32]; Fig 1). Chimera 1 was generated by replacing the P1-HCPro bi-cistron of a PPV-GFP construct (construct pLX-PPV, [40]) with that of PVY [construct pLX-(PVY P1-HCPro)-PPV, [25] and Fig 1]. Chimeras 2 and 3 each contained a beneficial mutation that was acquired naturally during infection of *N. benthamiana* plants, at HCPro amino acids 352 and 454, respectively, and chimera 4 contained both mutations combined (Fig 1).

An additional chimera 2 variant in which an *mRFP* cistron replaced the *GFP* one, creating chimera 2R [construct pLX (PVY P1-6x-HCPro_352Ile→Thr_)-mRFP] was synthesized using a two-step overlapping PCR approach, amplifying first overlapping PCR fragments PCR1, using as forward oligo PPV NIb F *Cla*I (5′-GCCAATCC**ATCGAT**TGGAGG-3′) and reverse oligo NIb mRFP R overlap (5′- CATGGCCTGATGTACTACGACATTGGACTCACC-3′), and pLX-PPV as template; and PCR2, using as forward oligo NIb mRFP F overlap (5′- ATGTCGTAGTACATCAGGCCATGGCCTCCTCCGAG-3′), and reverse oligo mRFP 3´kpn minus stop (5´- GTT**GGTACC**GGCGCCGGTGGAGTGGCGGCCC-3′), using the binary construct pROK-mRFP [22] as template. We then combined the gel-excised and cleaned PCR1 and 2 fragments and performed an overlapping fusion PCR, the first 5 cycles without adding any oligos. Reaction conditions were 98 °C 2 min; then 5 cycles of 50 °C 10 s and 72 °C 5 min. After that, flanking forward of PCR1 and reverse of PCR2 oligos were added, and the PCR proceeded for 30 more cycles of 98 °C 10 s, 63 °C 10 s and 72 °C 15 s. The fusion PCR product thus obtained was digested with *Cla*I and *Kpn*I, and cloned into the chimera 2 construct, thus creating the construct expressing chimera 2R.

An additional chimera 5 in which the original Gly at amino acid 329 of the HCPro encoded by chimera 3 was replaced by a Glu [construct pLX-(PVY P1–6 × -HCPro_329Gly→Glu_)-PPV] was created with an approach similar to the one used to obtain chimera 2R: we first made a single nucleotide substitution GGG (Gly)→G**A**G (Glu) using a two-step overlapping PCR approach, amplifying first overlapping PCR fragments PCR1, using as forward oligo 5′- TCCTCCCGTGCTACAAGA-3′ and reverse oligo 5′- CCGAATC**CTC**TTTTGGTAAATCAACAAATTTTTGG-3′, and PCR2, using as forward oligo 5′- CCAAAA**GAG**GATTCGGAGATGTTATACATTGC-3′ and reverse oligo 5′- CAGTTGTTTGTGAGCCAAA-3′ using the chimera 3 as template for amplification. We then combined the gel-excised and cleaned PCR1 and 2 fragments and performed an overlapping fusion PCR, the first 5 cycles without adding any oligos. Reaction conditions were 98 °C 2 min; then 5 cycles of 50 °C 10 s and 72 °C 5 min. After that, flanking forward of PCR1 and reverse of PCR2 oligos were added, and PCR proceeded for 30 more cycles of 98 °C 10 s, 56 °C 10 s and 72 °C 20 s.

The fusion PCR product thus obtained was firstly digested with *Sal*I and *Xho*I and cloned into pROK2-P1-6x-HCPro [22] that expresses the polyhistidine-tagged WT form of HCPro that is in the chimera 3 variant), to create a binary vector expressing HCPro with Glu 329 instead of Gly 329, thus creating construct pROK2-P1-6x-HCPro_329Glu_, that expresses HCPro_329Glu_. We digested this construct with *NgoM*IV-*Nru*I, and transferred the elicited fragment into the similarly-digested chimera 3 construct pLX (PVY P1-6x-HCPro_454Leu→Arg_), creating construct pLX (PVY P1-6x-HCPro_329Gly→Glu, 454Leu→Arg_) that encodes the chimera 5.

### Plant tissues used in the assessment of viral titers and variant ratios in local and systemic infections

Binary constructs expressing chimeras 1, 2, 2R, 3, 4 and 5 were transferred to the same batch of electrocompetent *Agrobacterium tumefaciens* C58C1 cells. Cultures derived from one single colony were stored in 10% glycerol at -80 °C, until use. For agroinfiltrations, cell cultures were grown to exponential phase in Luria-Bertani medium with antibiotics at 28 °C. Bacterial cultures were then pelleted, and resuspended in 0.01 M MES, 0.01 M magnesium chloride medium with 0.002 M acetosyringone, to a final OD at 600 nm of exactly 0.3 for each cell culture, under the premise that this will induce overall equal amounts of T-DNAs to enter the cells of infiltrated discs. After two hours incubation at RT, bacterial media were vortexed and infiltrated with a needle-less syringe.

To assess viral titers and variant ratios in local infections, fully-expanded leaves of large but not-flowering *N. benthamiana* plants were agroinfiltrated, and at 5 dpi, leaf discs of 21 mm diameter were collected with a borer. Leaf discs were kept frozen until use in extractions of total protein and RNA. For confocal microscopy studies, freshly detached leaf discs were used.

To assess viral titers and variant ratios in systemic infections, young small *N. benthamiana* plants at the 4–5 leaf stage were agroinfiltrated in three lower leaves. Studies on systemic tissues were performed at 29 days post infiltration, except in the experiment shown in Fig 8C, in which systemic tissues were assessed at 14 dpi. Three 1 cm diameter discs were collected from two systemic leaves (2 + 1) with a borer, and kept frozen until analysis.

### Relative quantitation of viral-encoded proteins in local infections

Detection and quantitation of the relative accumulation of chimera variants, in single or mixed infections, in agroinfiltrated leaf tissues at 5 dpi were performed as described [32]. Briefly, total proteins in leaf discs were extracted in 400 µl of extraction buffer (0.1 M Tris-HCL pH 8, 10 mM EDTA, 0.1 M LiCl, 1% β-mercaptoethanol and 1% SDS). Extracted proteins were mixed 1:1 with Laemmli 2x buffer, boiled for 5 minutes, clarified by bench centrifugation, resolved in 15% SDS-PAGE gels, and transferred to PVDF nylon membranes by electro-blotting (western blot). Protein bands corresponding to the GFP or coat protein (CP) of PPV were detected using a rabbit polyclonal antibody to GFP, kindly provided by Professor Lesley Torrance (The James Hutton Institute, UK), or a commercial antiserum to the viral coat protein (CP) of PPV (cat. 07163S/500. Loewe Biochemica), respectively. The detected protein bands were subjected to densitometry analysis using ImageJ software. A rabbit polyclonal antiserum to HCPro [41] was also used, to detect and quantify PVY HCPro in agropatch assays (see below). To detect the mRFP expressed by chimera 2R, a commercial rabbit polyclonal antiserum was used (cat. 600–401-P16S. Rockland).

### Assessment of viral ratios by Sanger sequencing

One of the means to assess the relative accumulation of co-infecting chimeras in a particular leaf sample was through Sanger sequencing. To do this, total RNAs were Tryzol-extracted from 100 µl of total protein extracts, precipitated 1:1 with isopropanol, resuspended in water, cleaned further twice with ethanol precipitation, and the resulting RNAs were quantified by Nanodrop. The total RNAs were treated with TURBO DNase (Ambion) to remove any DNA from the sample. The 3´-region of the *HCPro* cistron was then amplified by RT-PCR, using reverse primer 5′- GATTTGTGGCTTGTAAATGC-3′ and SuperScript III reverse transcriptase with reduced RNase H activity to obtain ss cDNAs, following the manufacturer’s instructions (Invitrogen). This oligo is complementary to a sequence in the 5´-region of the *P3* cistron, downstream of *HCPro*. Amplification by PCR of the ss cDNAs was performed with High-Fidelity Phusion DNA polymerase (Invitrogen), using oligo pairs Forward 472 + SpeI (5´-ATG**ACTAGT**CAGGAGTCAGCAGAAAATGC-3´) and Reverse 595 + KpnI (5´-ATG**GGTACC**GATTTGTGGCTTGTAAATGC-3´) that allowed the cloning of the amplified fragments to high-copy plasmid pBluescript SK2+ through their added *Spe*I and *Kpn*I sites.

For each plant sample, either the miniprep-purified clones (at least 10 clones/sample) or the direct RT-PCR fragment were Sanger-sequenced by Secugen S.L., using sequencing oligos Forward 472 + SpeI. Contig analyses of the sequences obtained were performed with Vector NTI Advance 11.0 software (Invitrogen).

To determine variant proportions in single nucleotide polymorphic sites within triplets encoding amino acids 329, 352, and 454 (depending on the chimera pairs in co-infections) we used QSV software (University of Leeds, UK; [34]).

### Assessment of viral ratios by nanopore sequencing

A second approach to assess the relative accumulation of co-infecting chimeras in a particular leaf sample was through nanopore sequencing. Hence, ss cDNAs obtained using oligo 5′- GATTTGTGGCTTGTAAATGC-3′ as described in the previous paragraph were converted into ds cDNAs using a Second Strand cDNA Synthesis Kit (Invitrogen). Circa 100 ng of ds cDNAs/sample were submitted to Secugen S.L., for sequencing with nanopore technology, using a library preparation Kit Native Barcoding V14 (NBD24).

Identification and analysis of the viral sequences was performed with our own commands and scripts implemented in Python (version 3.10) (S1 Text). Briefly, raw FASTQ files were quality-filtered using NanoFilt (v2.8.0) with a minimum Phred score threshold of 20 (Q20). To isolate viral sequences, reads were mapped against the indexed HCPro reference sequence using minimap2 (v2.30-r1287). Only reads with robust viral alignment were retained. Indel-containing reads were kept only when the overall alignment was consistent with the reference sequence and did not interfere with the identification of new mutations and chimera classification. The alignment process yielded several hundred viral reads per sample. Downstream analyses to determine chimera ratios combined automated quantification using custom Python 3 scripts and manual inspection via IGV (Integrative Genomics Viewer, v2.19.7) [42]. Indeed, for chimera detection, all viral reads were analyzed based on the triplet reads corresponding to aa 352 and 454. Although the R10.4.1 (V14) chemistry achieves a raw accuracy exceeding 99% (Q20), a minimum detection threshold of 3% and a sequencing depth of ≥ 79 were applied for minority variant identification to minimize false positives arising from nanopore sequencing errors (S1 Table). Furthermore, all new mutations were independently verified at the single read level using IGV software, enabling read-by-read validation of sequence mutations.

### Assessment of GFP- and mRFP-derived fluorescence in local co-infections

We co-expressed chimeras 2R and 3 in fully expanded leaves by agroinfiltration. At 5 dpi, fresh leaf tissues were used to visualize mRFP- and GFP-derived fluorescence in epidermal cell fields, using a Leica SP5 confocal microscope with a 40x oil objective, and settings for detection of mRFP (excitation 561 nm/ emission 575–640 nm) and GFP (excitation 488 nm/emission 500–530 nm).

Samples from agroinfiltrated patches were also analysed by western blot, for the serological detection and quantitation of relative GFP and mRFP levels, as described above.

### HCPro structure analysis

We used AlphaFold version 3 [43], to generate 3D conformations of HCPro dimers tagged with 6xhistidines, and visualize the amino acid substitutions studied here. Of the five 3D-conformation options for each sequence received, we selected the one ranked 1. We then used UCSF Chimera 1.19 [44] to edit the conformations.

### Silencing suppression agropatch assays

6x-HCPro WT and 6x-HCPro_329Glu_ were transiently expressed from the corresponding binary vectors by agroinfiltration in fully expanded leaves of four *N. benthamiana* plants using the agroinfiltration technique, to test for their relative abilities to suppress the silencing of a transiently co-expressed *GFP* reporter. Relative levels of GFP and of the two HCPro variants were assessed by western blot analysis of total protein extracts, using specific antibodies, and ImageJ software, as described above.

### Statistics analysis

The titers of the five chimeras in local single infections and in co-infections were obtained from western blot analysis. Statistical assessment was performed with one-way analysis of variance (ANOVA) for independent groups, followed by two-tailed post hoc tests depending on the homogeneity of variances: Scheffe tests for equal variances and Games–Howell tests for unequal variances (Figs 2 and 8B). Pairwise comparisons of western blot data were performed using two-tailed unpaired Student t-test or Mann–Whitney U tests as appropriate in Figs 5B and 8B.

For statistical assessment of co-infecting variant proportions within local infiltrated discs or systemic discs (Figs 3, 4A and S2), we used a two-tailed Wilcoxon signed-rank test for paired samples. The null hypothesis assumed equal representation of variant A and variant B within co-infected discs, corresponding to a theoretical median pA of 0.5. These proportions were estimated from the relative peak areas of Sanger sequencing chromatograms obtained from independent biological samples.

For pairwise comparisons involving two independent groups [chimera 1 in treatment 1 + 2 vs. chimera 1 in treatment 1 + 3 in (Fig 3) and chimera 3 or 4 in WT vs. in D2D4 in systemic tissues in (S2 Fig)], differences were assessed using two-tailed unpaired the Mann–Whitney U test**.**

Statistical assessment of differences in the proportion of either chimera 3 or chimera 2 in local co-infections under three experimental groups (WT/RT, D2D4/RT, and WT/HT; Fig 4A) was performed using a one-way analysis of variance ANOVA for independent groups, followed by two-tailed Games–Howell post hoc tests to account for unequal variances among groups.

## Supporting information

S1 Fig Assessment of the use of Sanger sequencing of RT-PCR fragments to determine ratios in co-infections of chimeras 2 + 3. (A) RT-PCR amplification of a circa 500 nucleotide-long 3´-region of the *HCPro* cistron, from local tissues at 5 days post-infiltration, cloning and sequencing of 20 individual clones indicated presence of homologous recombinants (6 out of 20). (B) RT-PCR amplification, cloning and sequencing from total RNAs of single infections of chimeras 2 or 3 produced only reads corresponding to the parental virus. However, when the two total RNAs were combined prior to RT-PCR a quarter of the sequenced clones (5 out of 20) were recombinants, indicating that they were created by template switching during amplification with Phusion polymerase. (C) The ratios of chimera 2 vs. 3 deducted from the sequencing of clones with or without recombinants were broadly comparable (1^st^ vs. 3^rd^ bars). And those deducted from clones including recombinants and those obtained from direct sequencing of the amplified PCR fragment its and analysis with QSV software were also similar (1^st^ vs. 2^nd^ bar).
(TIF)

S2 Fig Variant prevalence in the systemic tissues of individual plants co-inoculated with two chimeric variants that carry one or two beneficial mutations in the *HCPro* cistron. Charts show co-infection ratios in systemic tissues of individual plants, 29 days after they had been co-infiltrated locally with chimeras 3 + 4. Chimera 3 carries a beneficial mutation at HCpro amino acid 454 (Leu → Arg), while chimera 4 carries two beneficial mutations, at amino acid 352 (Thr → Ile), and 454 (Leu → Arg). Plants were kept under environments: WT/RT, wild-type *Nicotiana benthamiana* plants kept at 23 °C (room temperature), or D2D4/RT, in which silencing-compromised *N. benthamiana* were used instead of WT plants, kept both at RT. Prevalence was determined by electropherogram analysis of polymorphic nucleotide sites in Sanger-sequences of RT-PCR *HCPro* fragments from 8 individual plants/condition. Chimera 4 consistently predominated over chimera 3 in both environment conditions (*P < 0.05*. Wilcoxon test). No significant difference in chimera ratios was observed between WT and D2D4 plants (*P > 0.05*. Mann-Whitney U test).
(TIF)

S1 Table Additional mutations detected by nanopore sequencing in systemic infections. (DOCX)

S1 Raw Images The raw blots (antibody-developed, and Ponceau S-stained) that were used to create the figures and charts of the manuscript are shown in this file. (PDF)

S1 Text Commands and scripts followed for the analysis of nanopore data. (DOCX)
