# Supplementary material for: Competition among co-infecting potyviral chimeras with adaptive, single-nucleotide differences in HCPro: Properties, and influence of environment factors on outcomes
Source: PLoS Pathog. 2026 Aug 18;22(8):e1014529. doi: 10.1371/journal.ppat.1014529 (PMC13502915; doi:10.1371/journal.ppat.1014529)
Supplement: Supplementary file 1 [file ppat.1014529.s004.pdf]

Raw blots used for the Figures and Charts shown in Makki et al.,

Western blot Figures used to obtain **charts** shown in **MANUSCRIPT FIGURE 2A**

Figure A: Chimera 1 vs. chimera 2, and chimera 1 vs. chimera 3, local, 5 dpi, WT plants

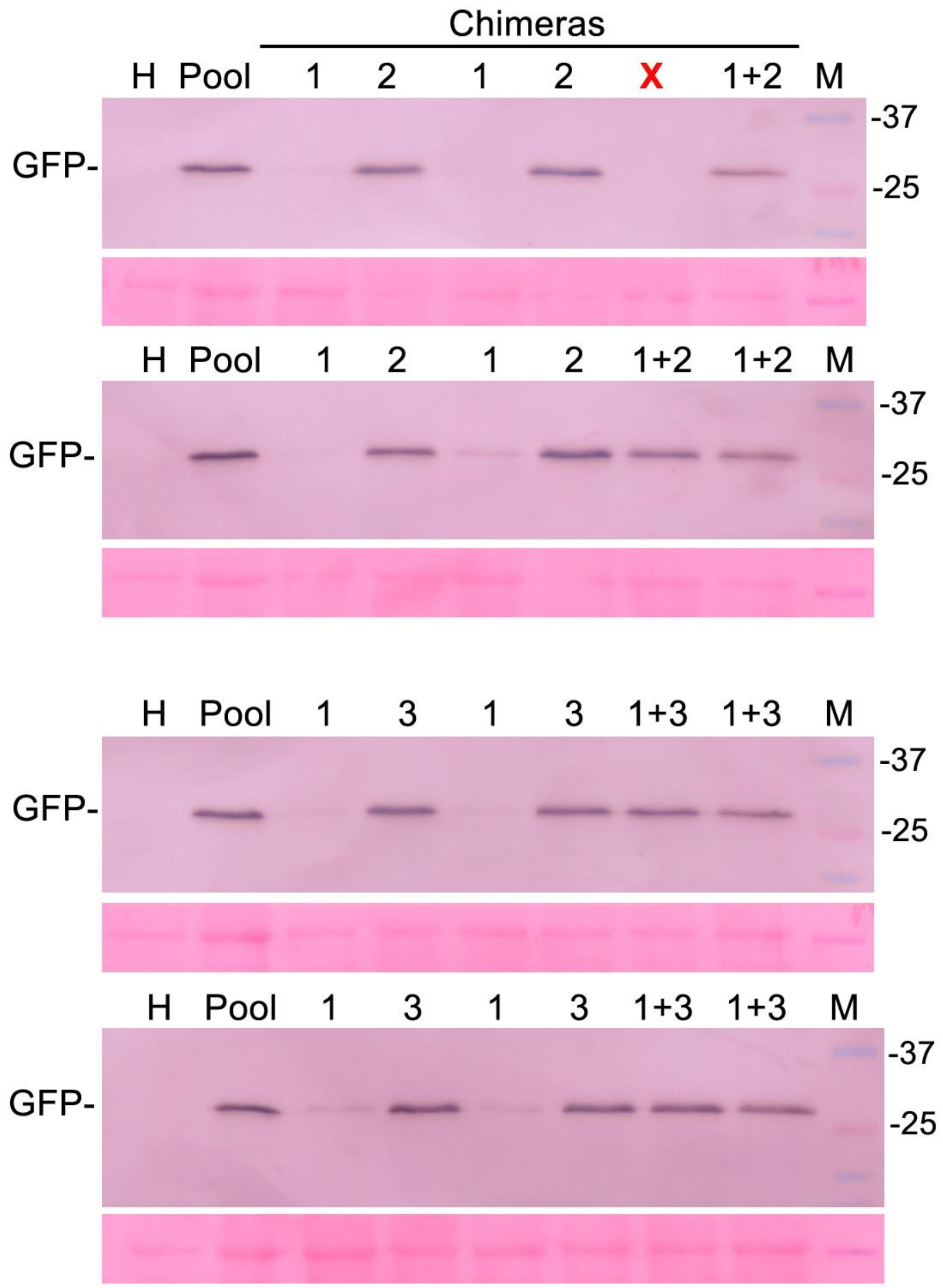

Raw blots for Figure A

Western blots

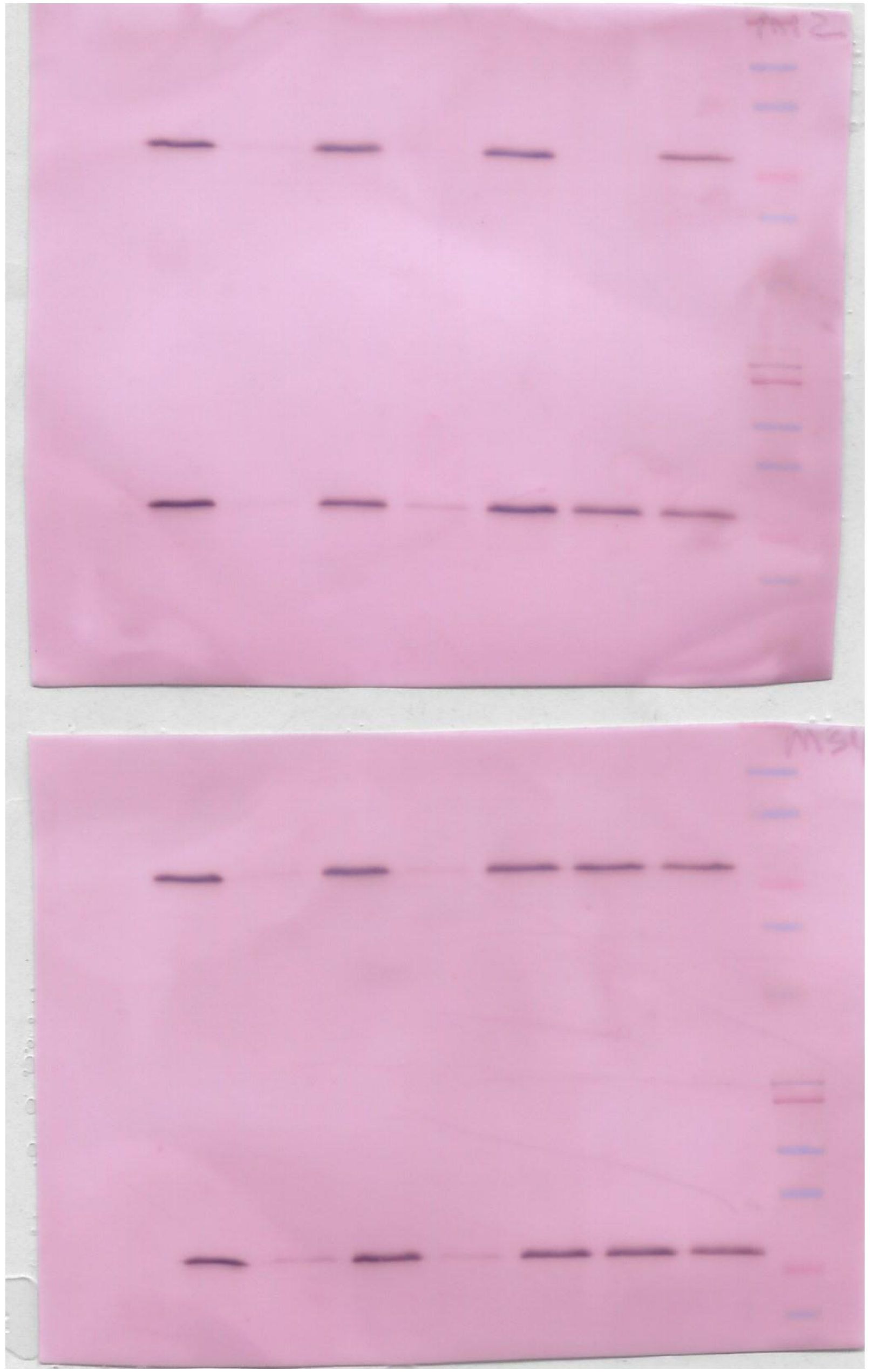

Ponceau-stained blots

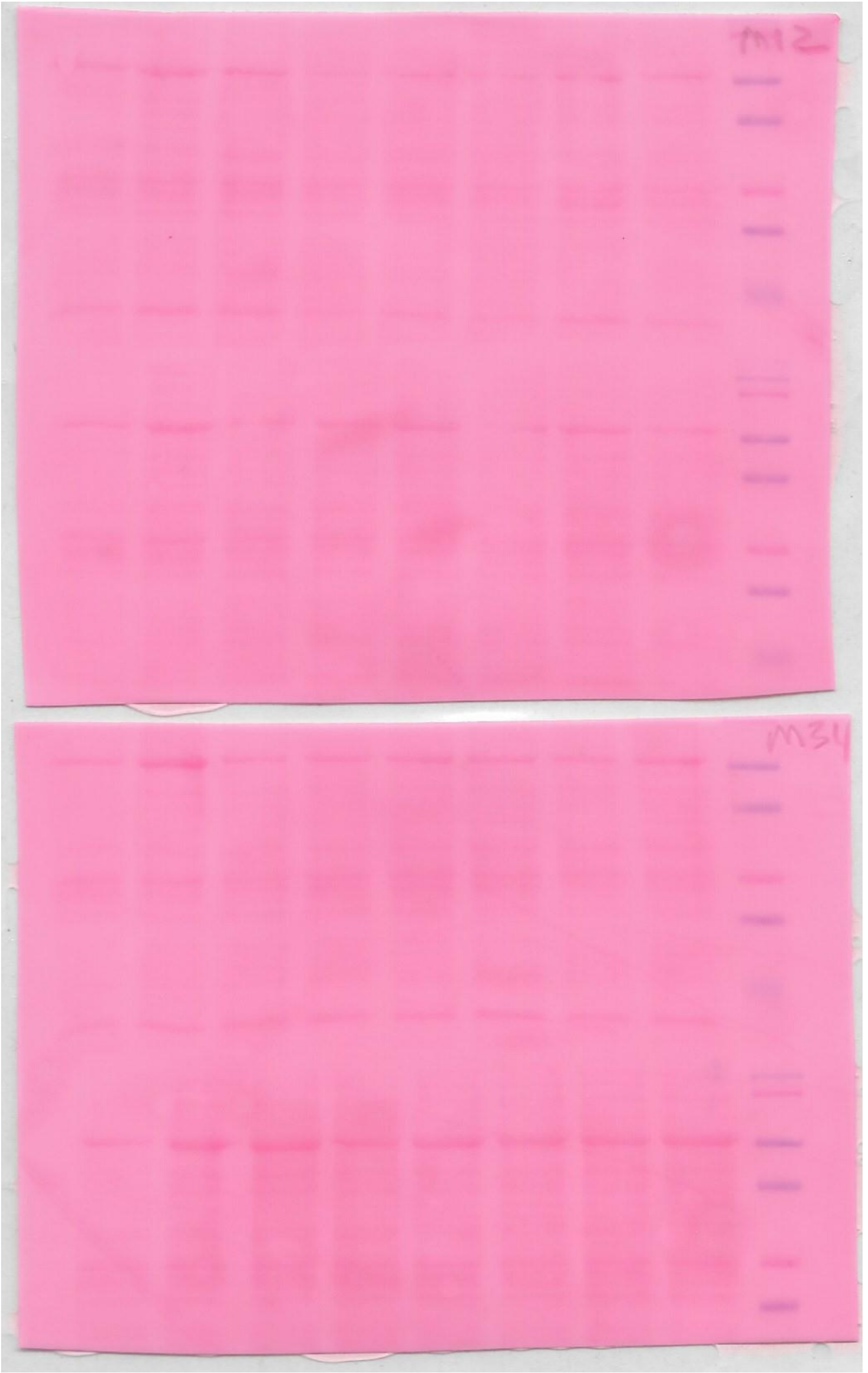

Western blot Figures used to obtain **charts** shown in **MANUSCRIPT FIGURE 2A** (arrows indicate the blots included in MANUSCRIPT FIGURE 2, as examples)

Figure B: Chimera 1 vs chimera 2, and chimera 1 vs. chimera 3, local, 5 dpi, WT plants

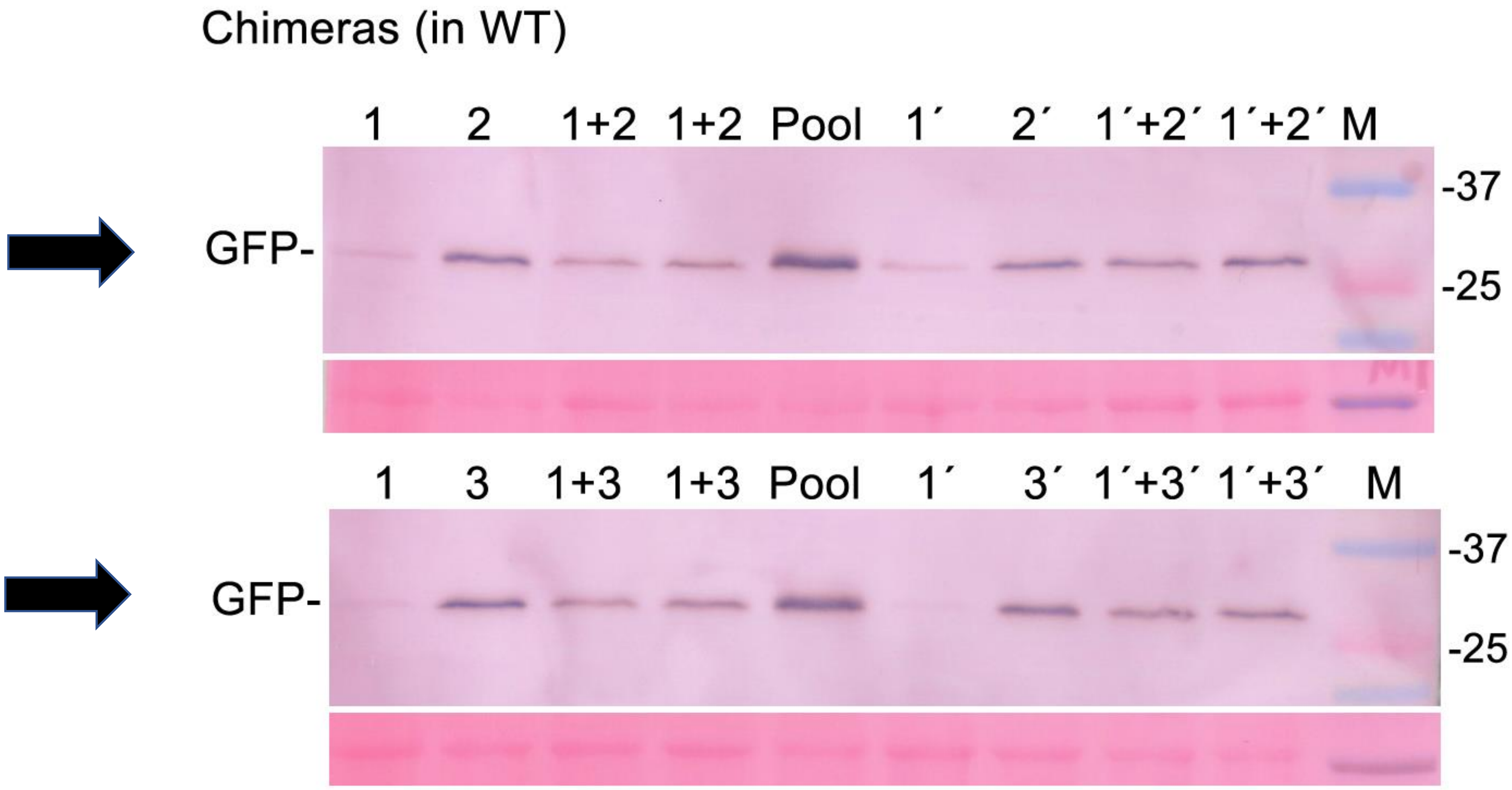

Raw blots for Figure B

Western blot

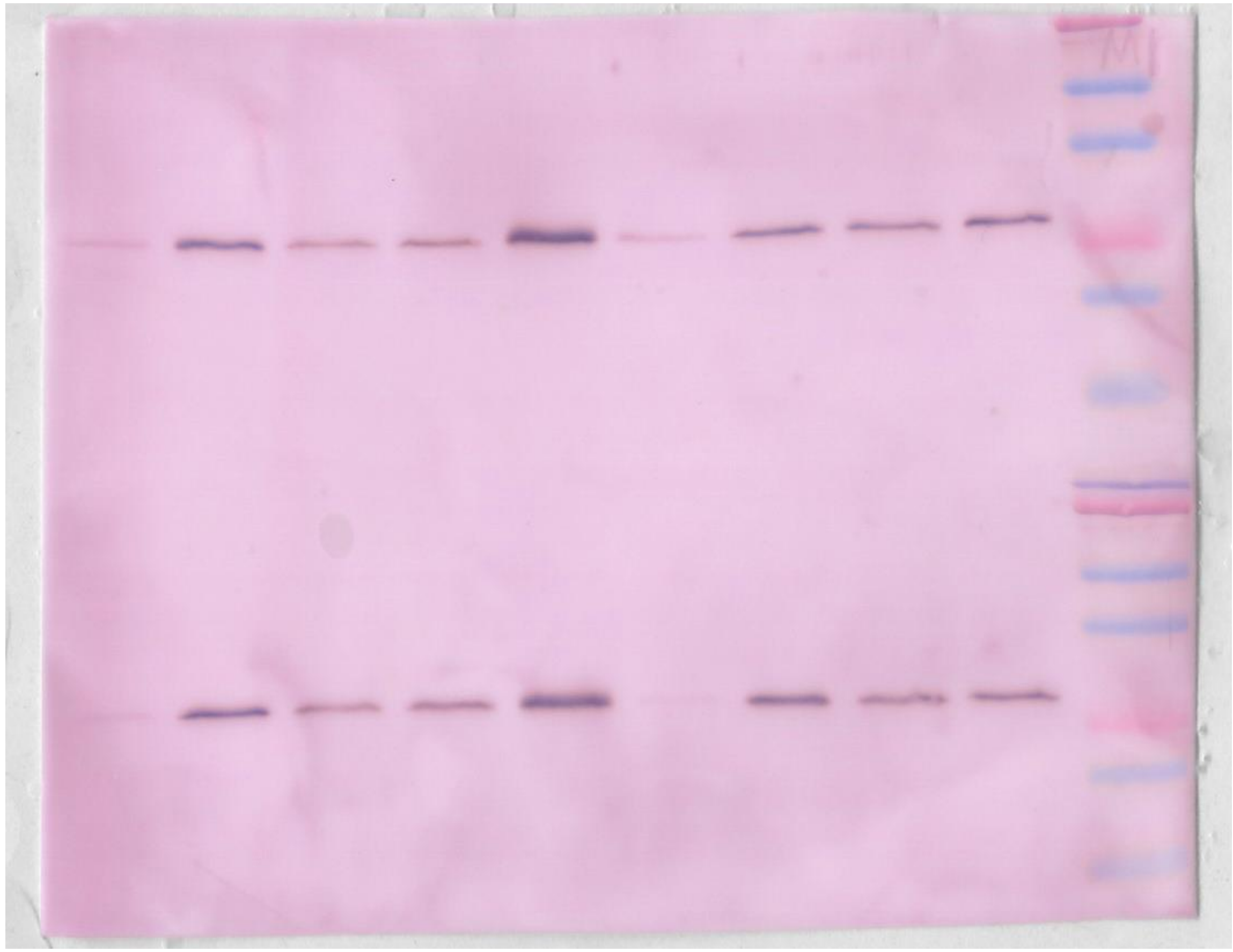

Ponceau-stained blot

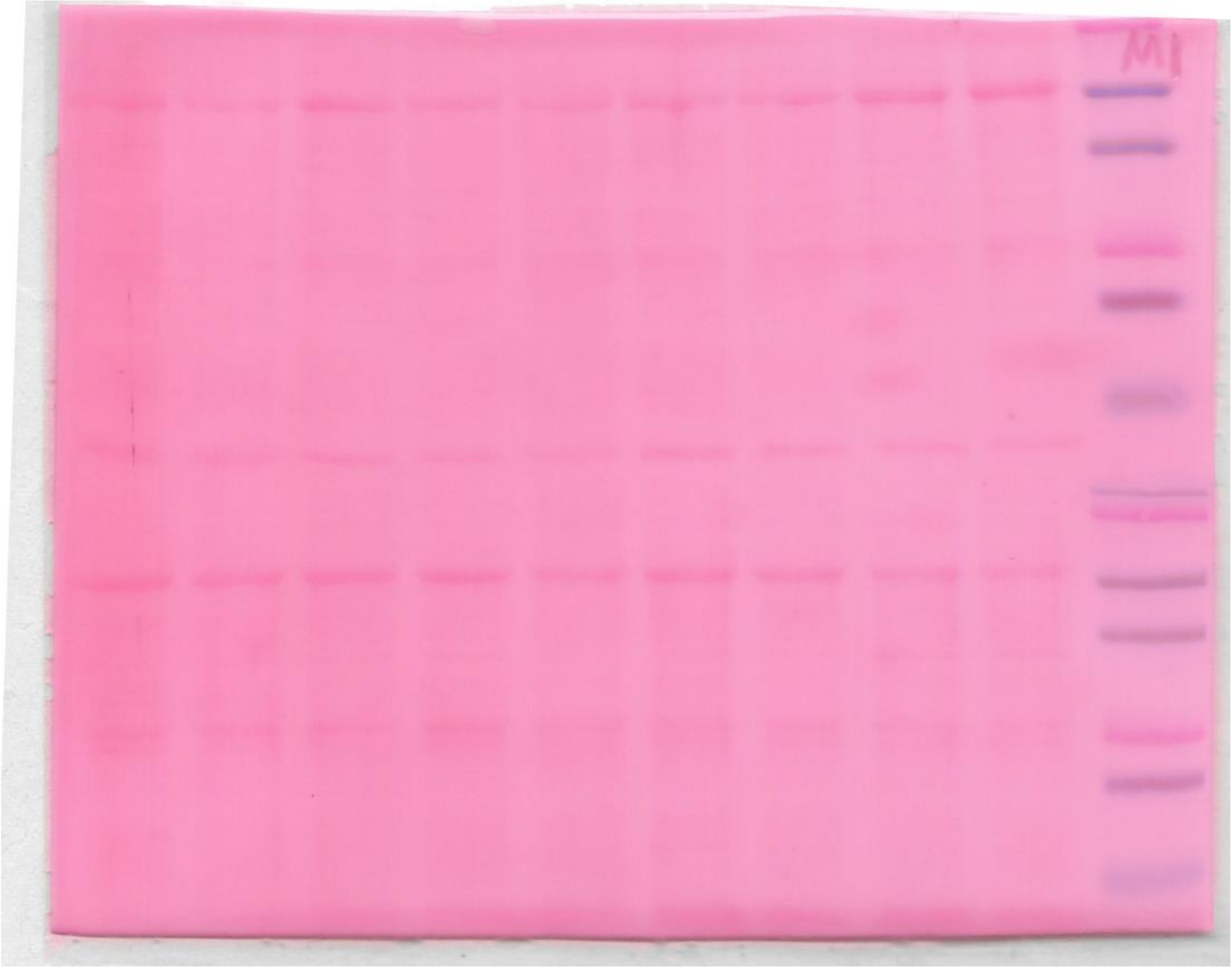

Western blot figures used to obtain charts shown in **MANUSCRIPT FIGURE 2A and 2B**

Figure C: Chimera 2 vs. chimera 3, and chimera 3 vs. chimera 4, local 5, dpi, WT and D2D4 plants

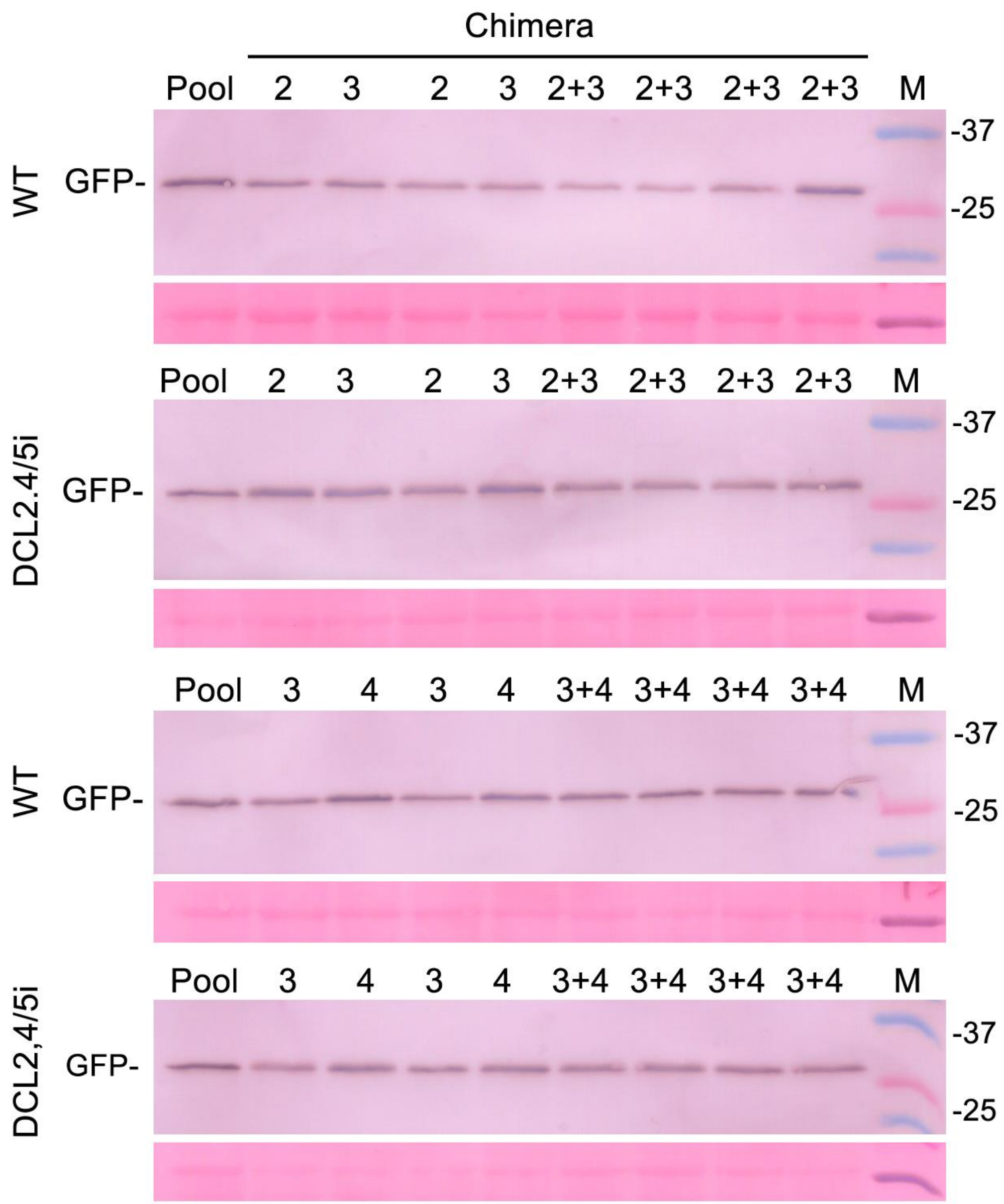

Raw blots for Figure C

Western blots

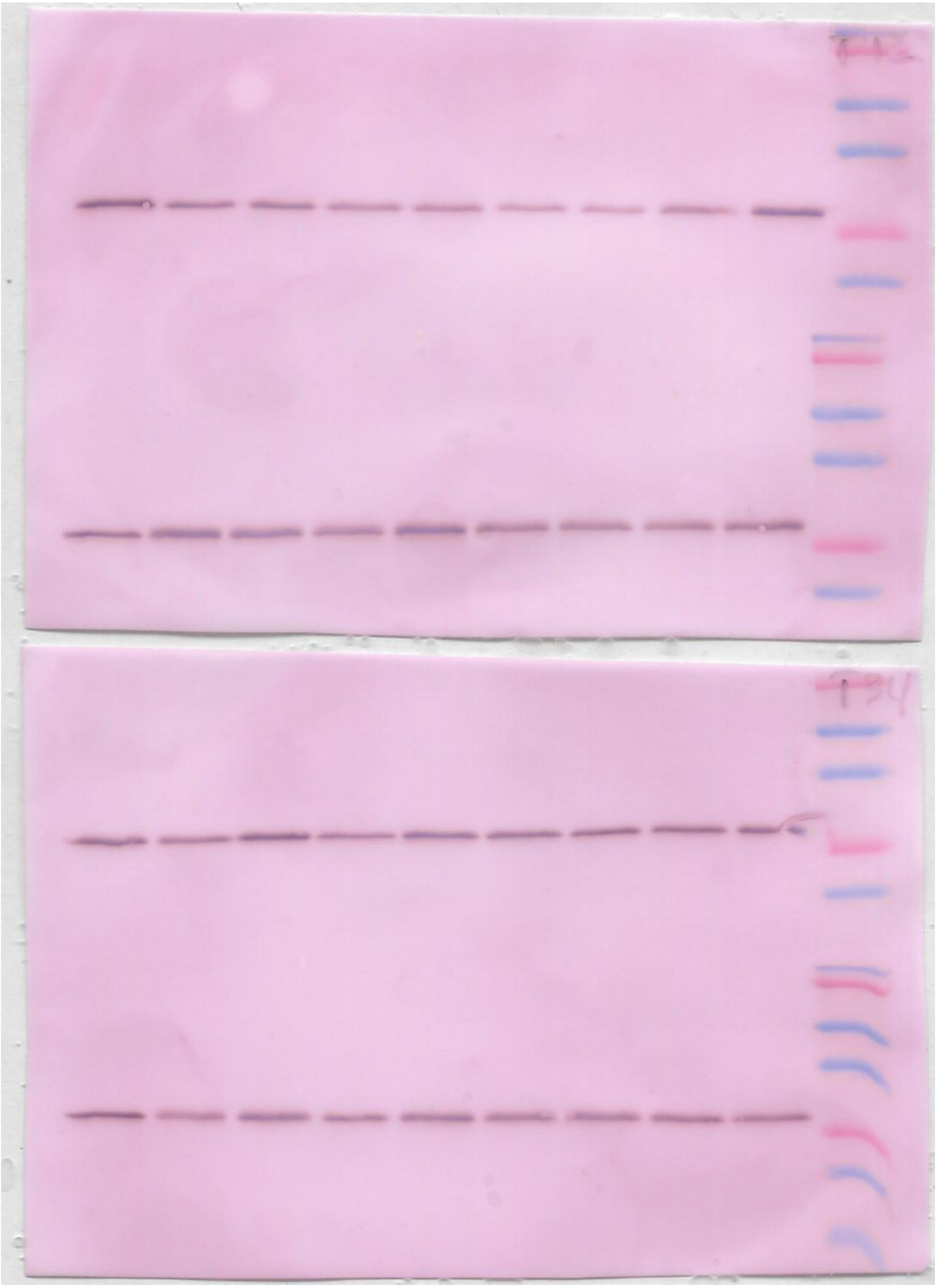

Ponceau-stained blots

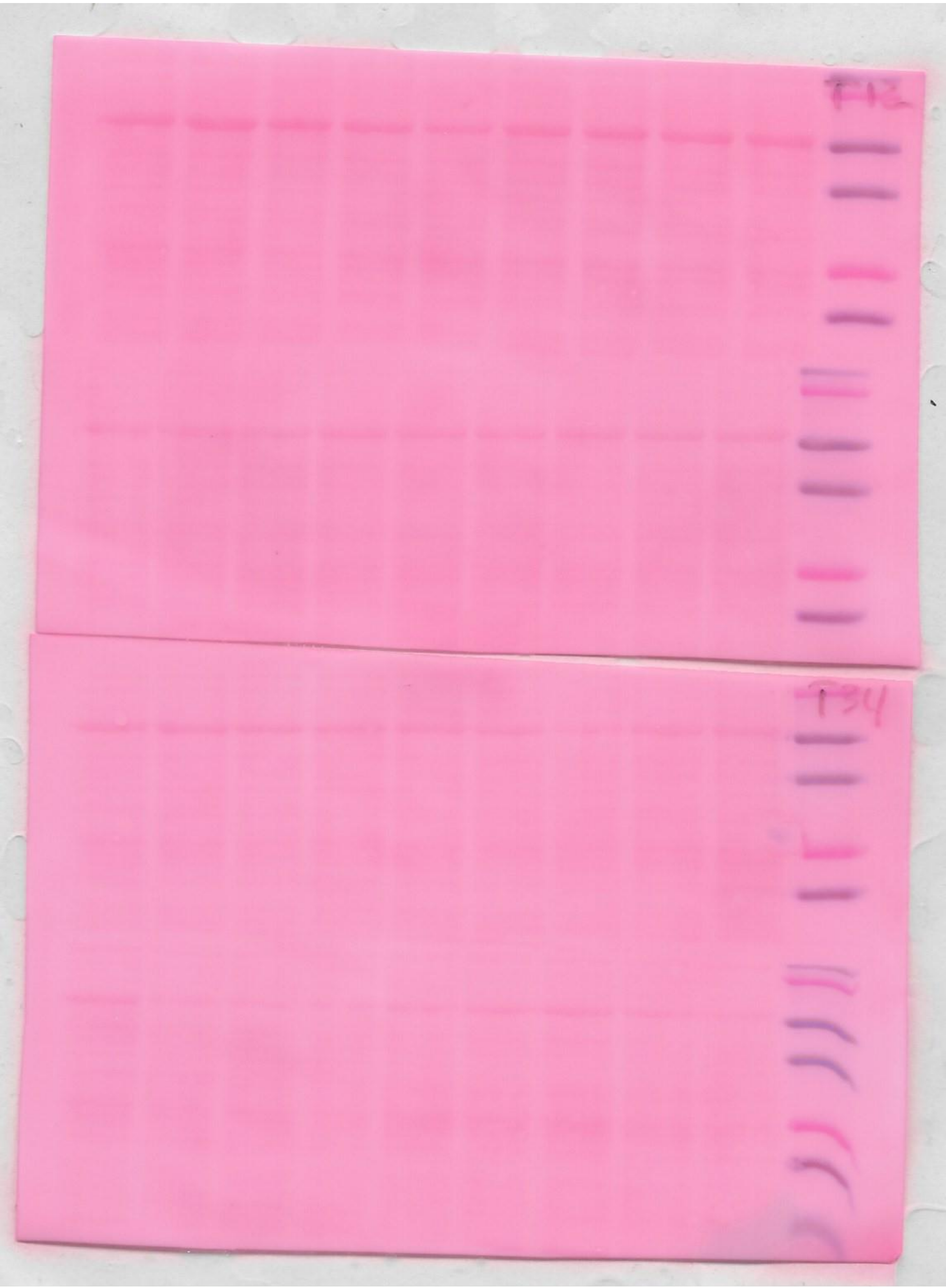

Western blot figures used to obtain charts shown in **MANUSCRIPT FIGURE 2A and 2B** (arrows indicate the blots included in MANUSCRIPT FIGURE 2, as examples)

Figure D: Chimera 2 vs. chimera 3, and chimeras 3 vs. chimera 4, local, 5 dpi, WT and D2D4 plants

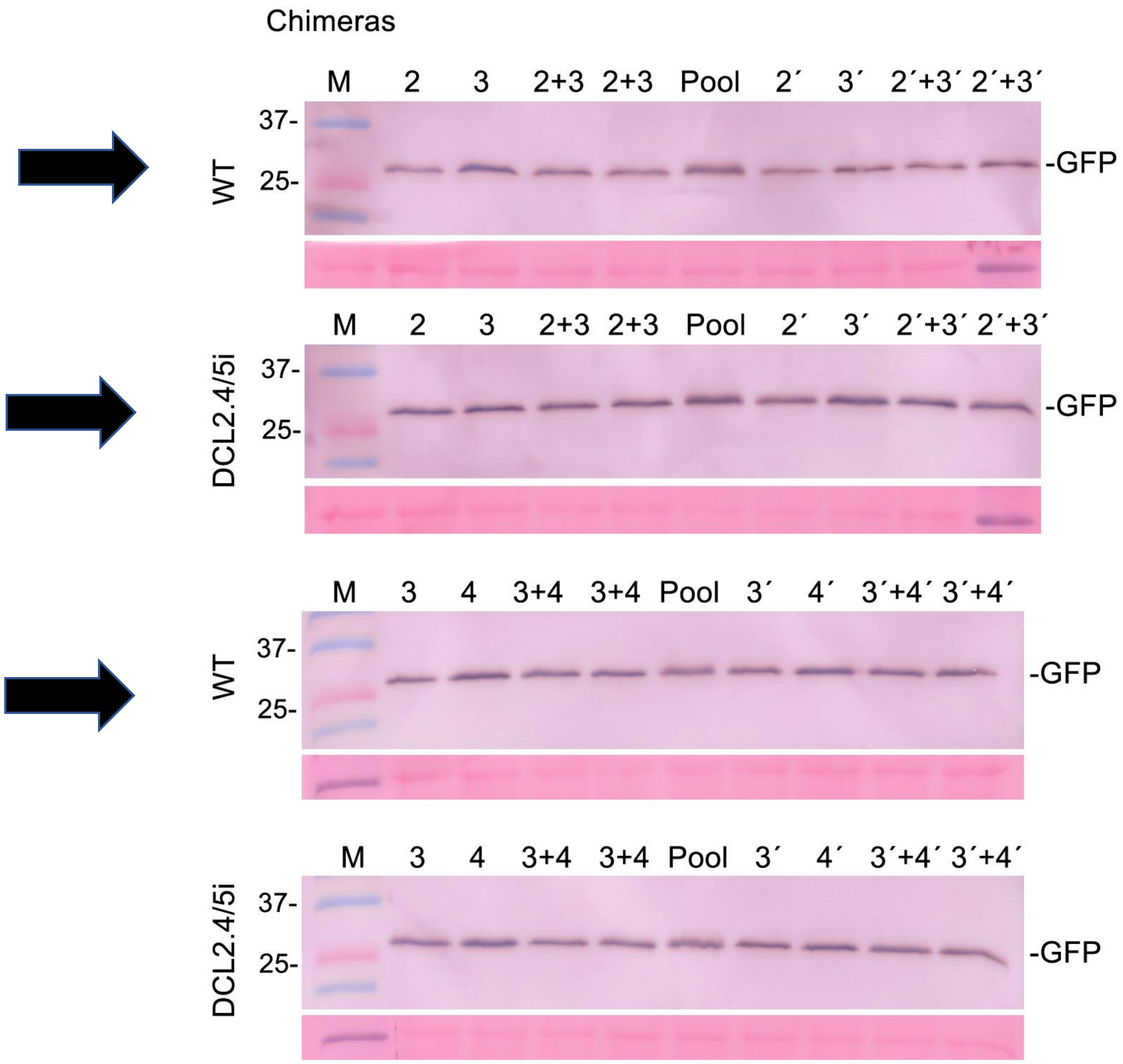

Raw blots for Figure D

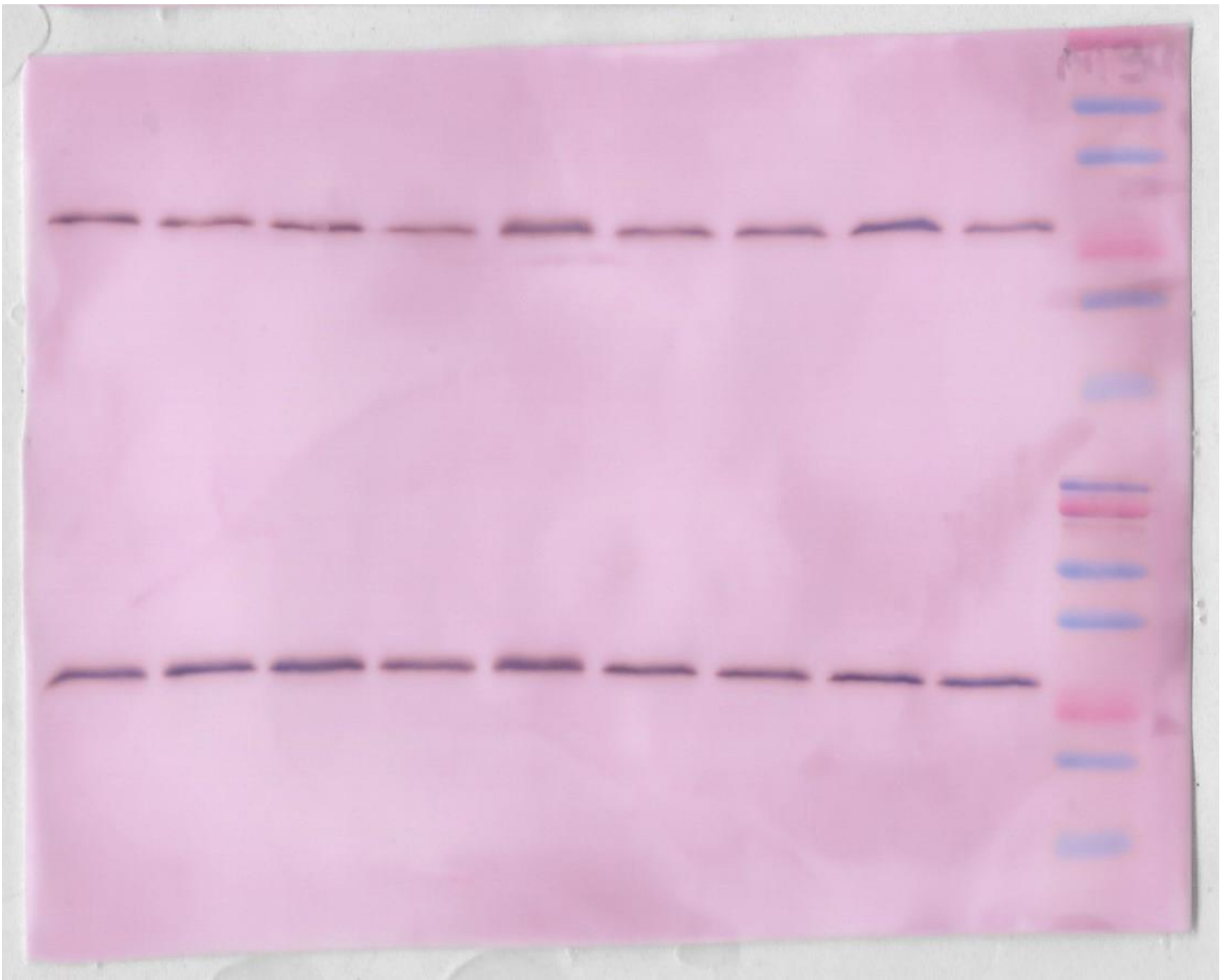

Western blots

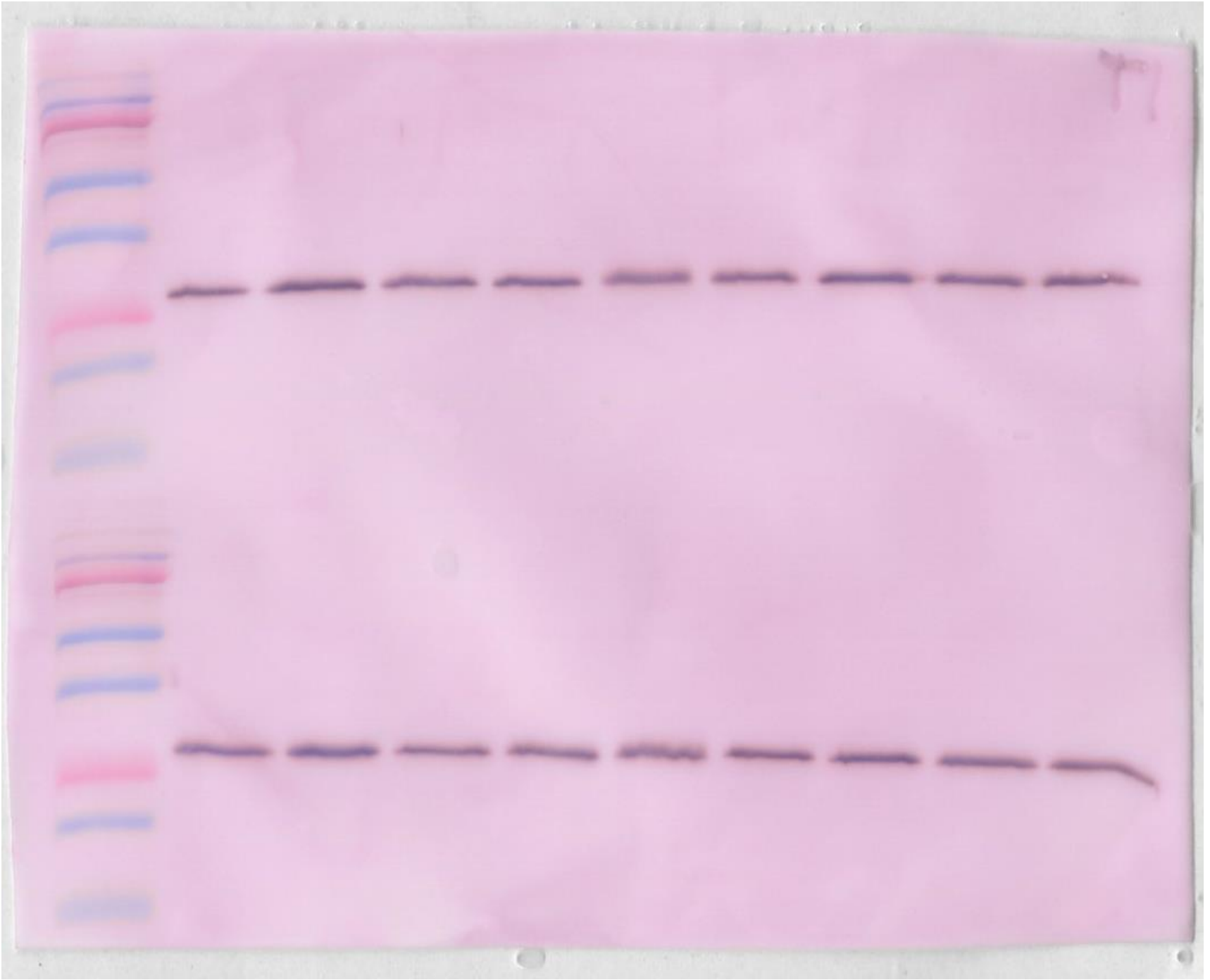

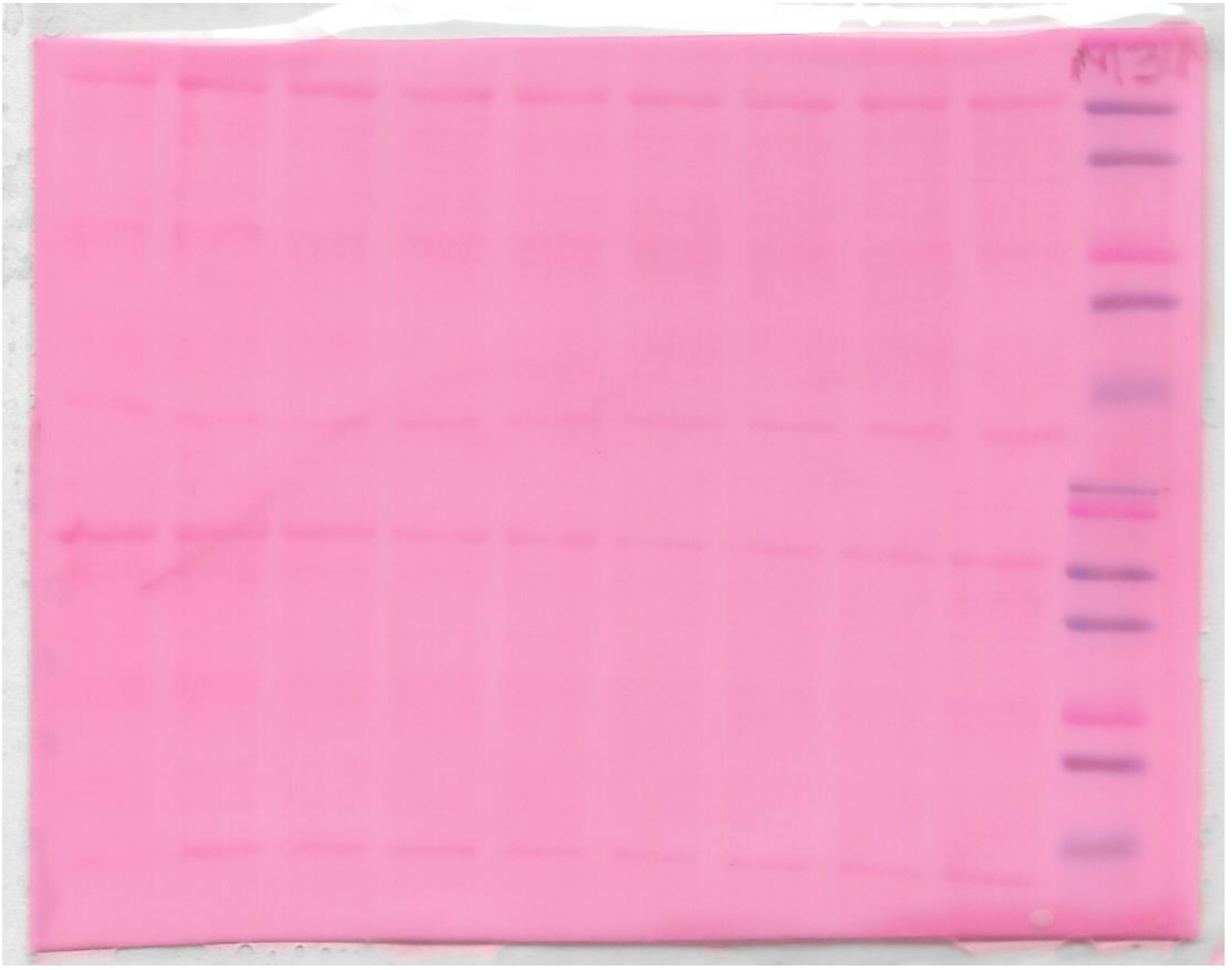

Ponceau-stained blots

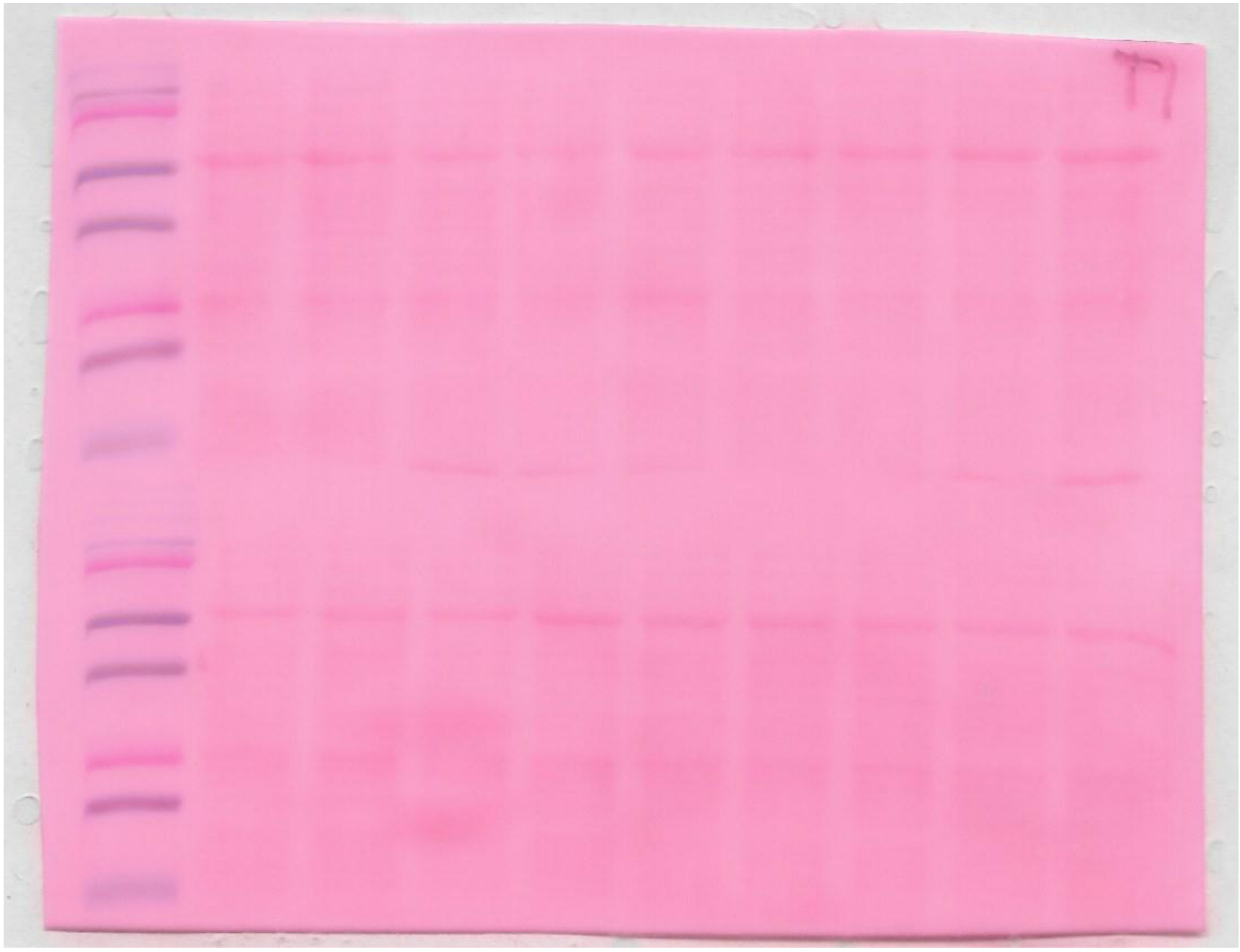

Western blot Figure used to obtain the chart shown in **MANUSCRIPT FIGURE 2B**

Figure E: Chimera 2 vs. chimera 3, local, 5 dpi, WT plants,at standard (RT) vs. high (HT) temperature

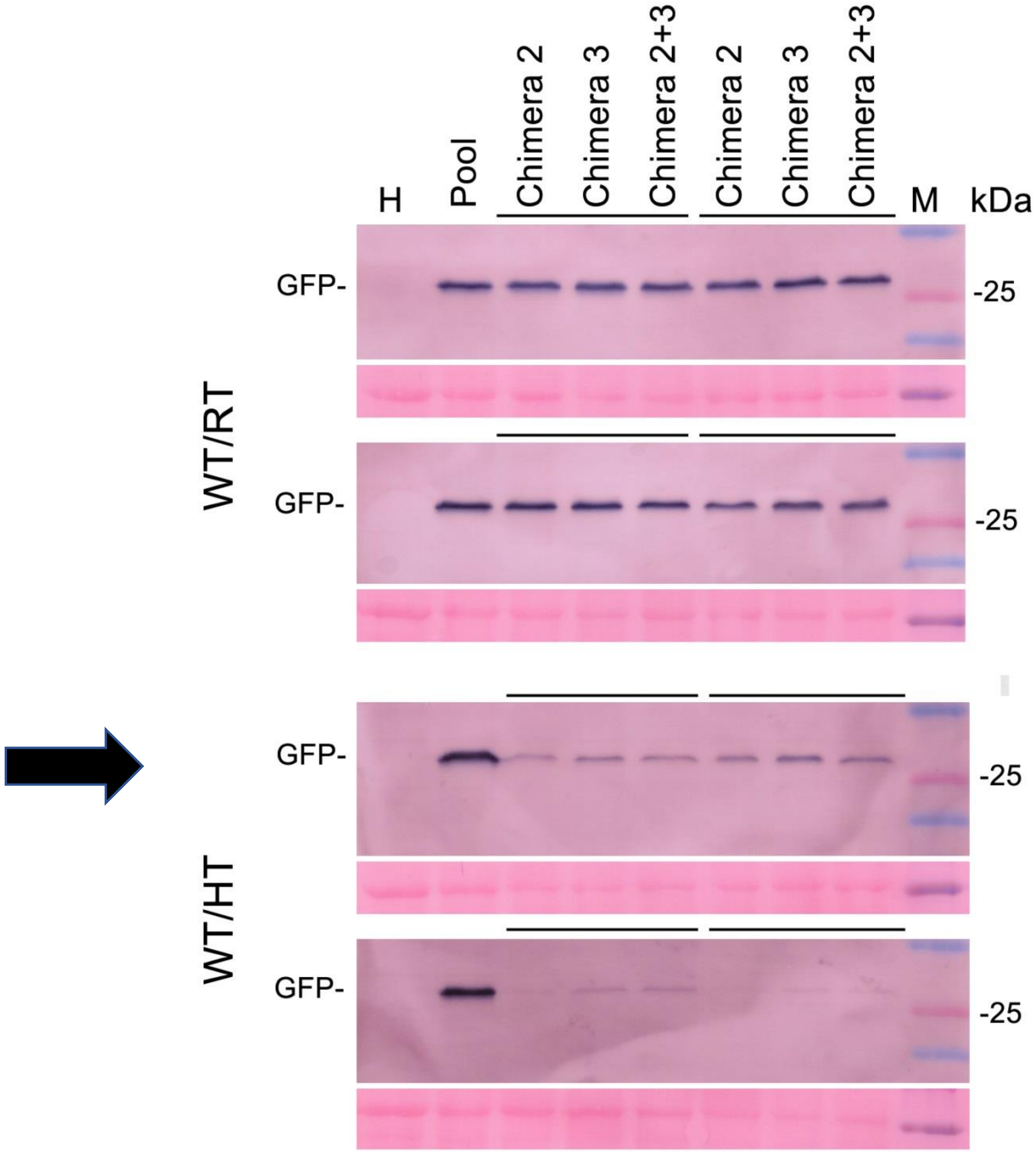

Raw blots for Figure E

Western blots

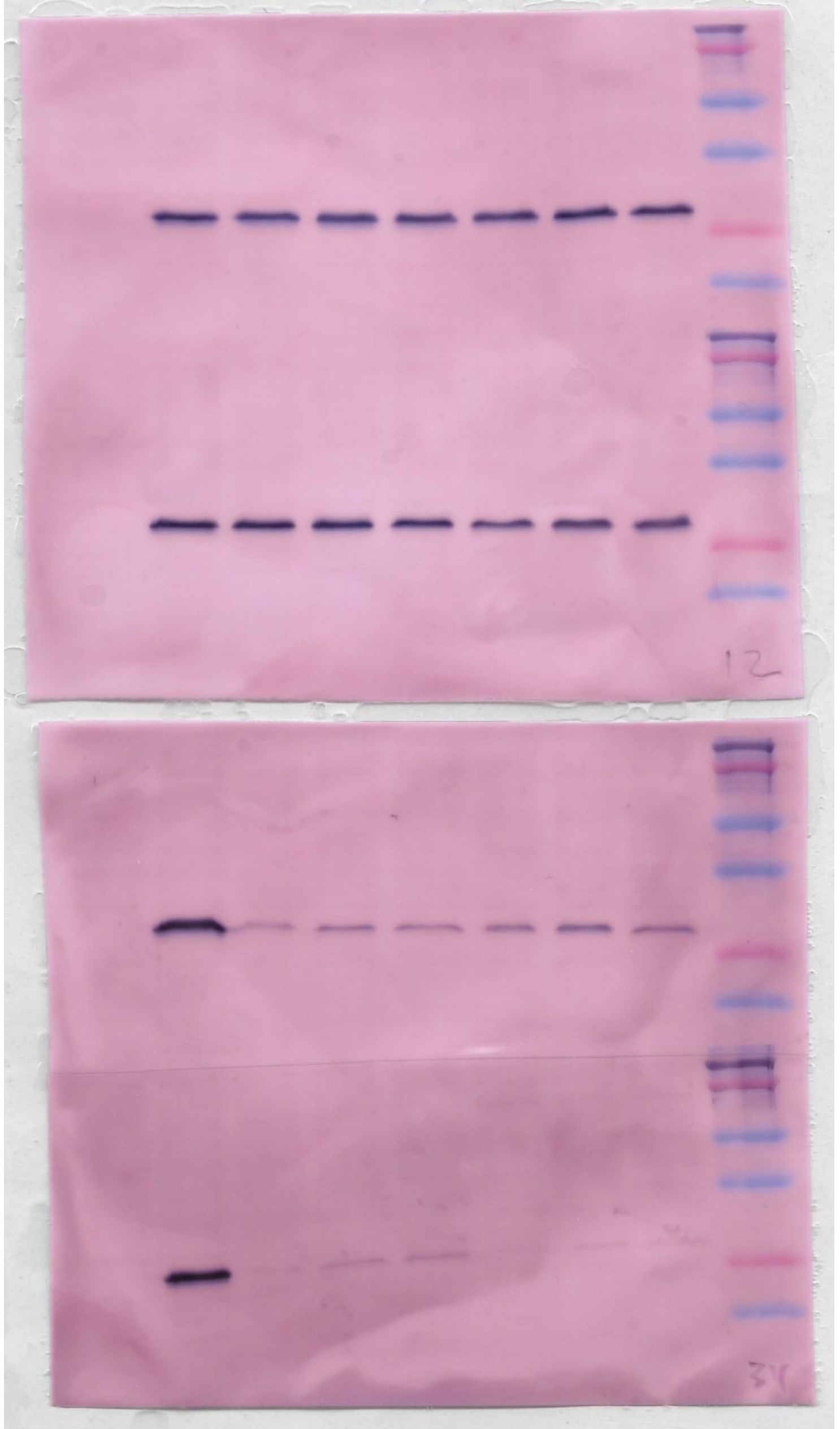

Ponceau-stained blots

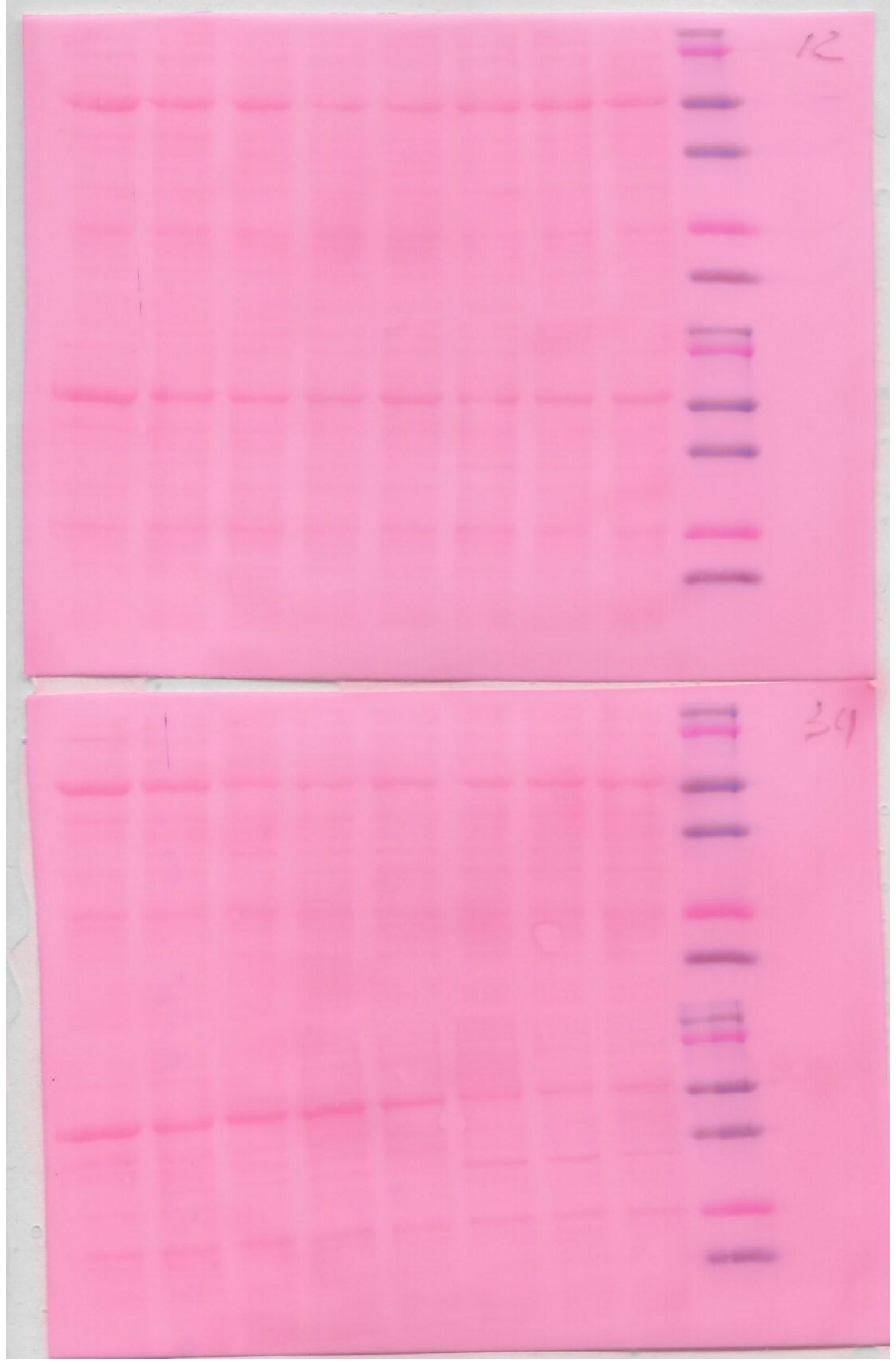

Western blot Figure used to obtain the chart shown in **MANUSCRIPT FIGURE 2B**

Figure F: Chimera 2 vs. chimera 3, local, 5 dpi, WT plants,at standard (RT) vs. high (HT) temperature

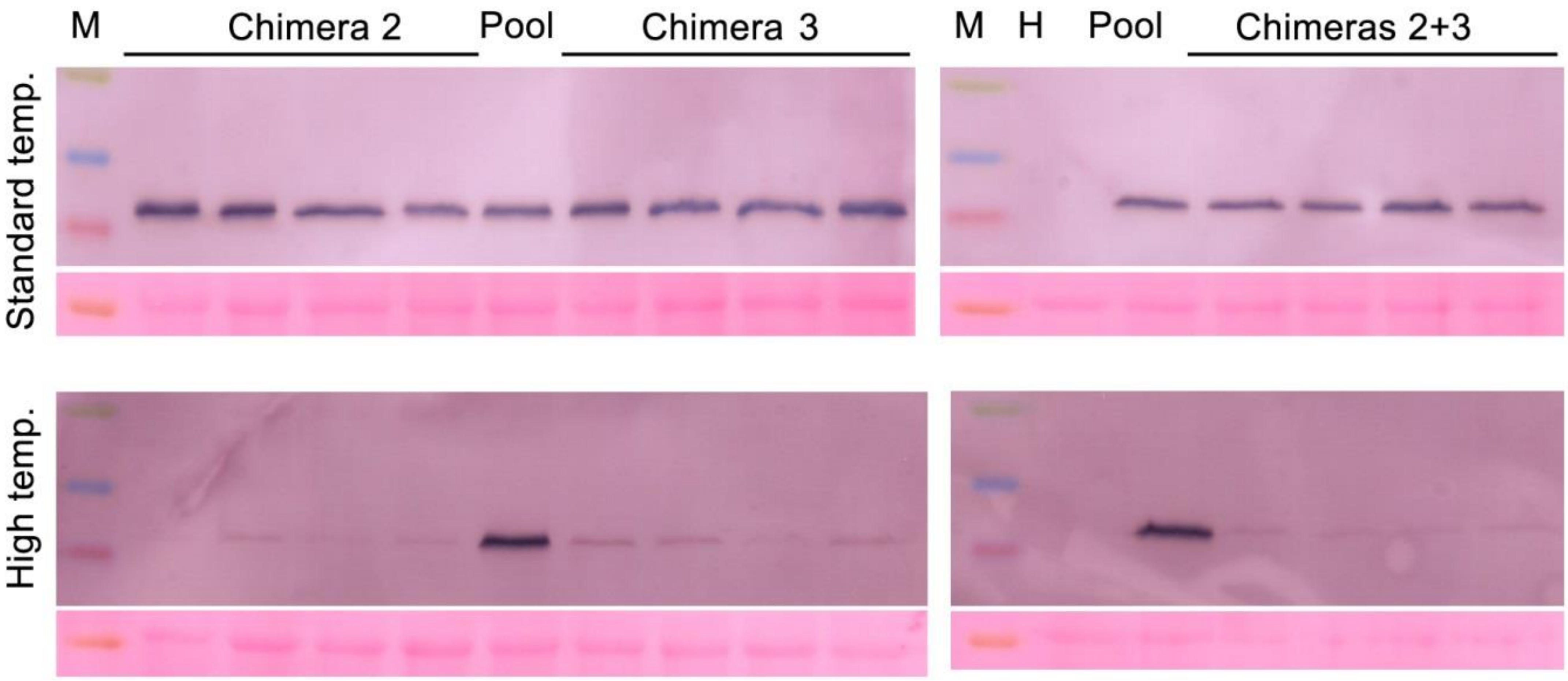

Raw blots for Figure F

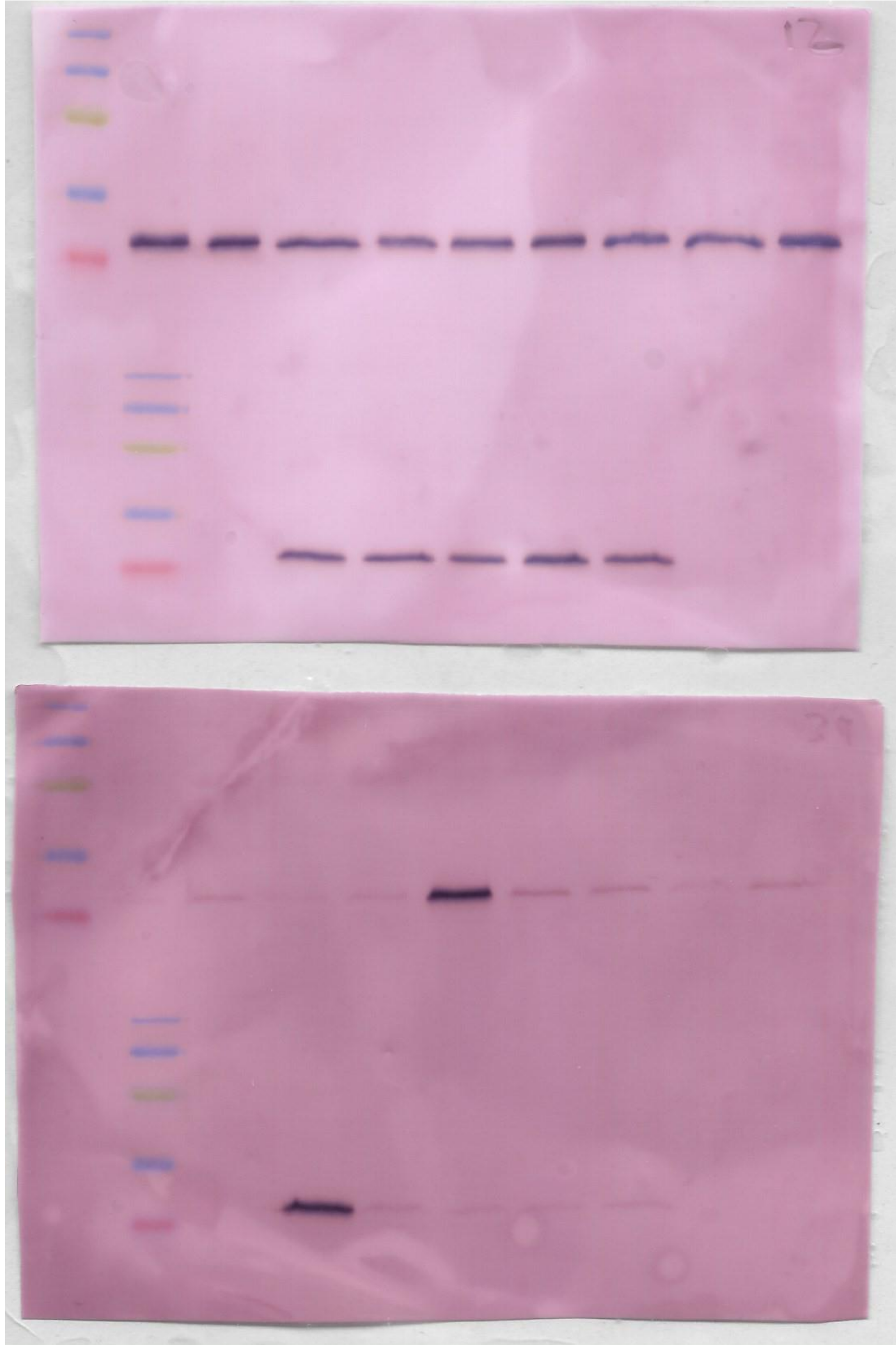

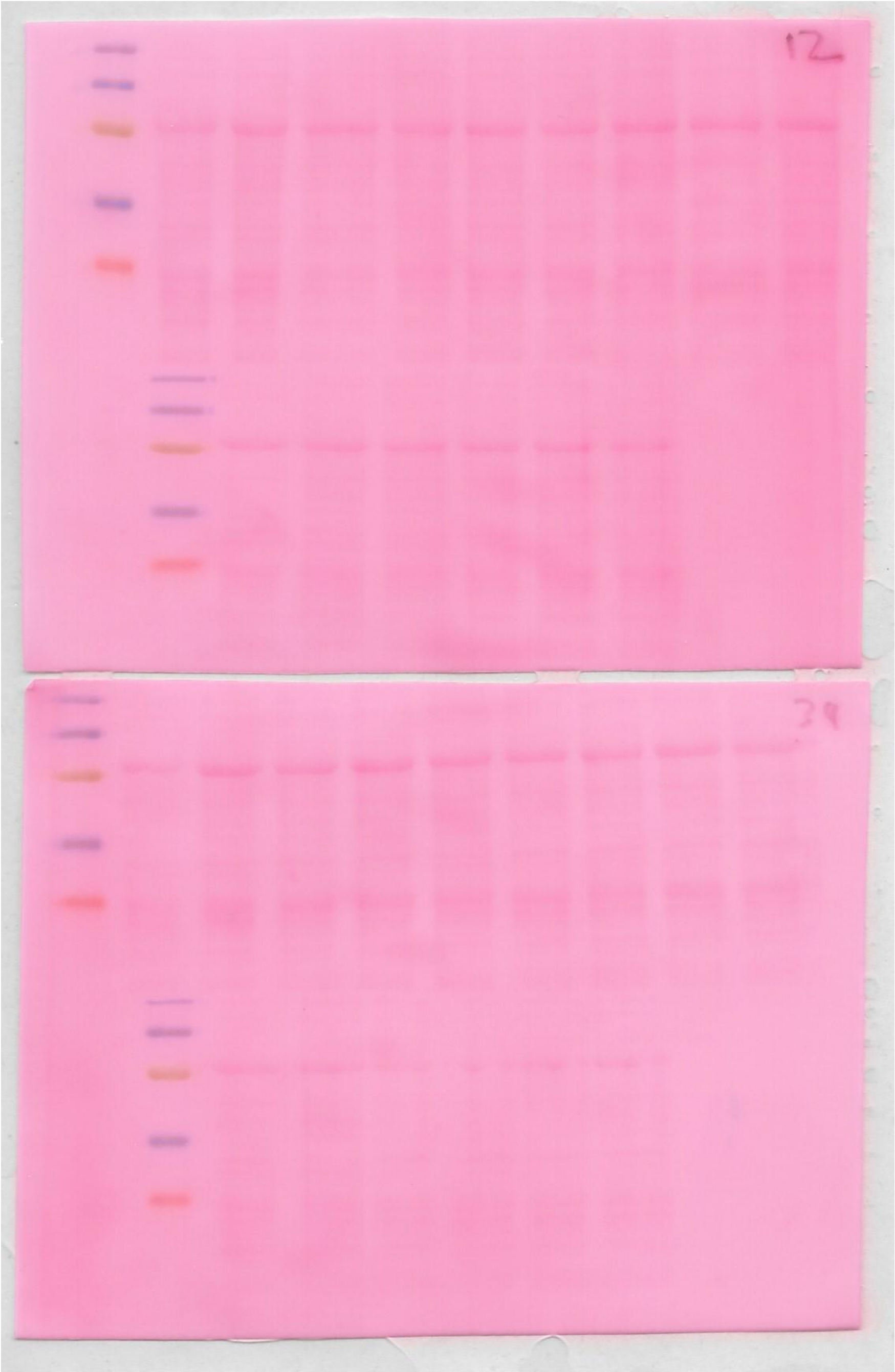

MANUSCRIPT FIGURE 5B

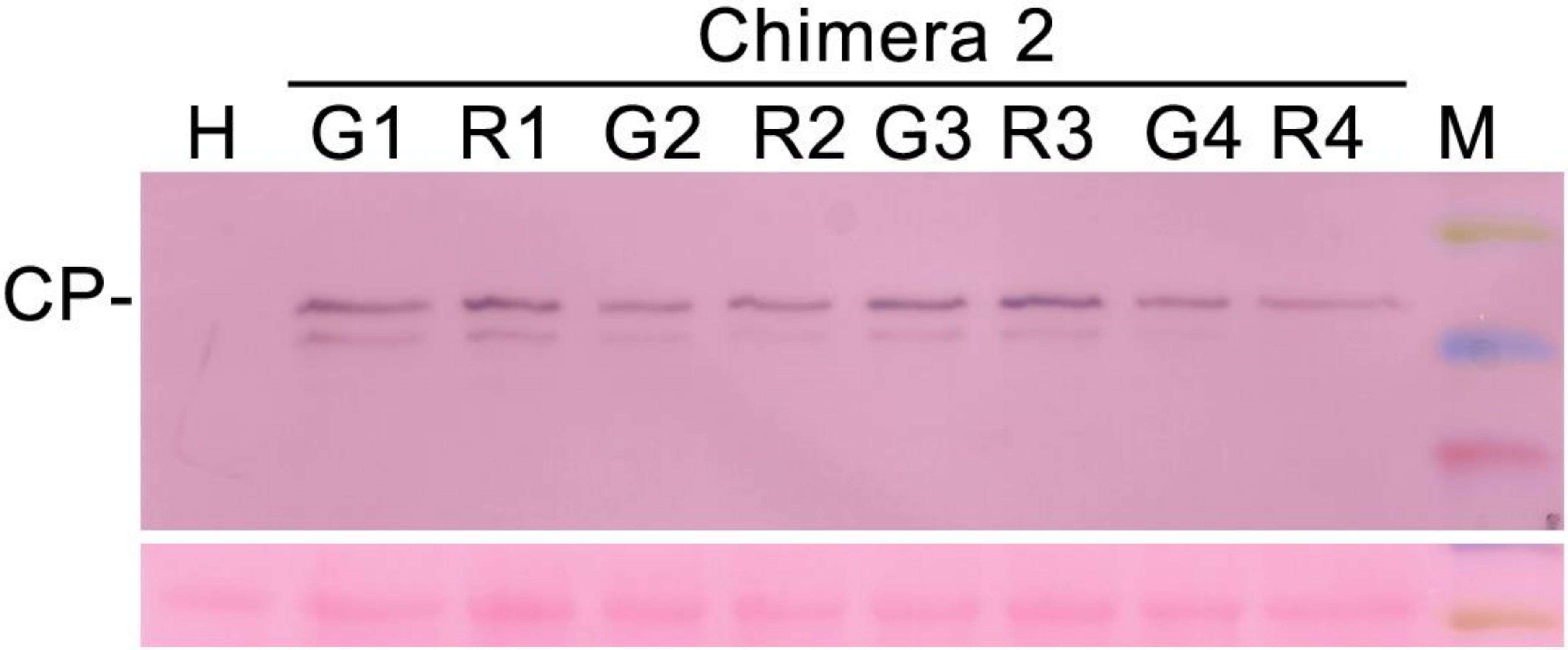

Raw blots for MANUSCRIPT FIGURE 5B

Western blot

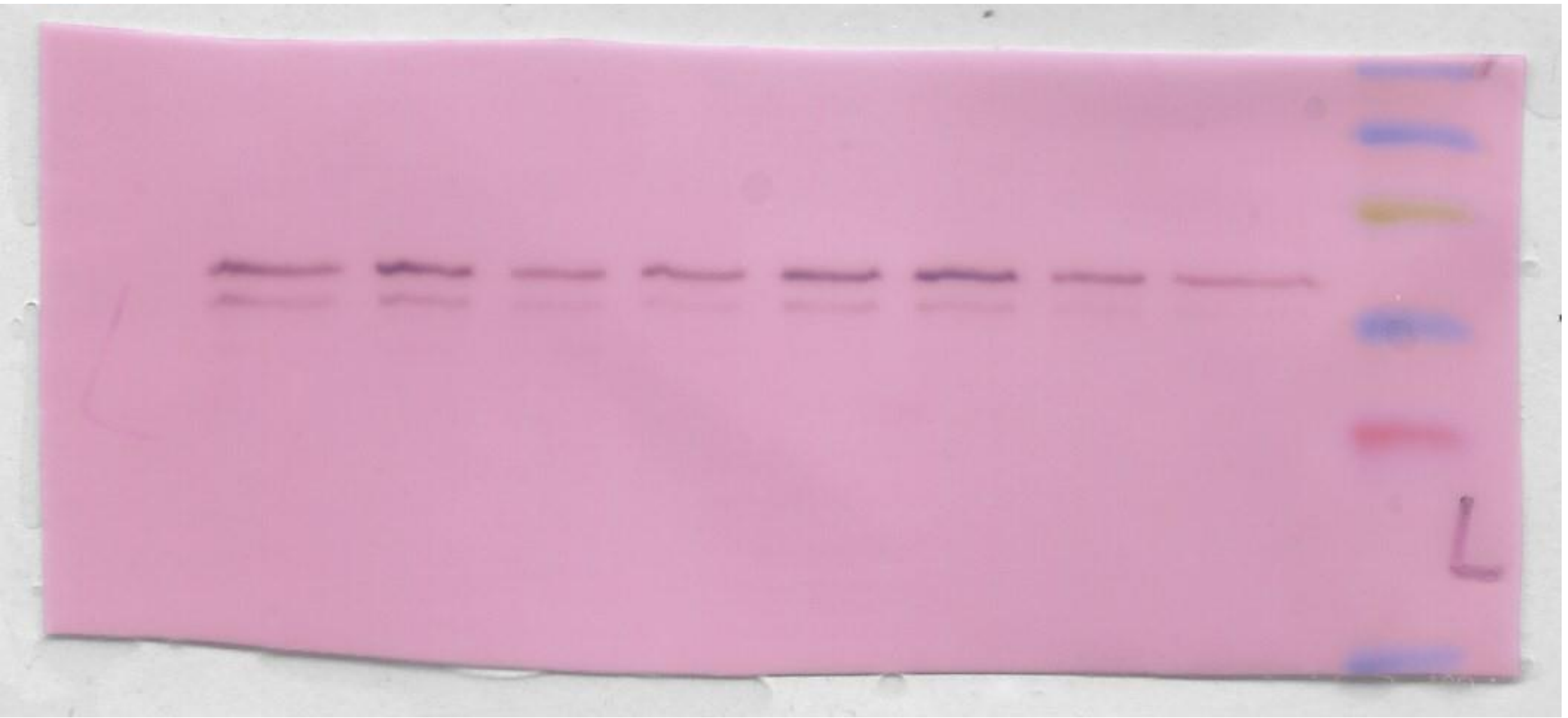

Ponceau-stained blot

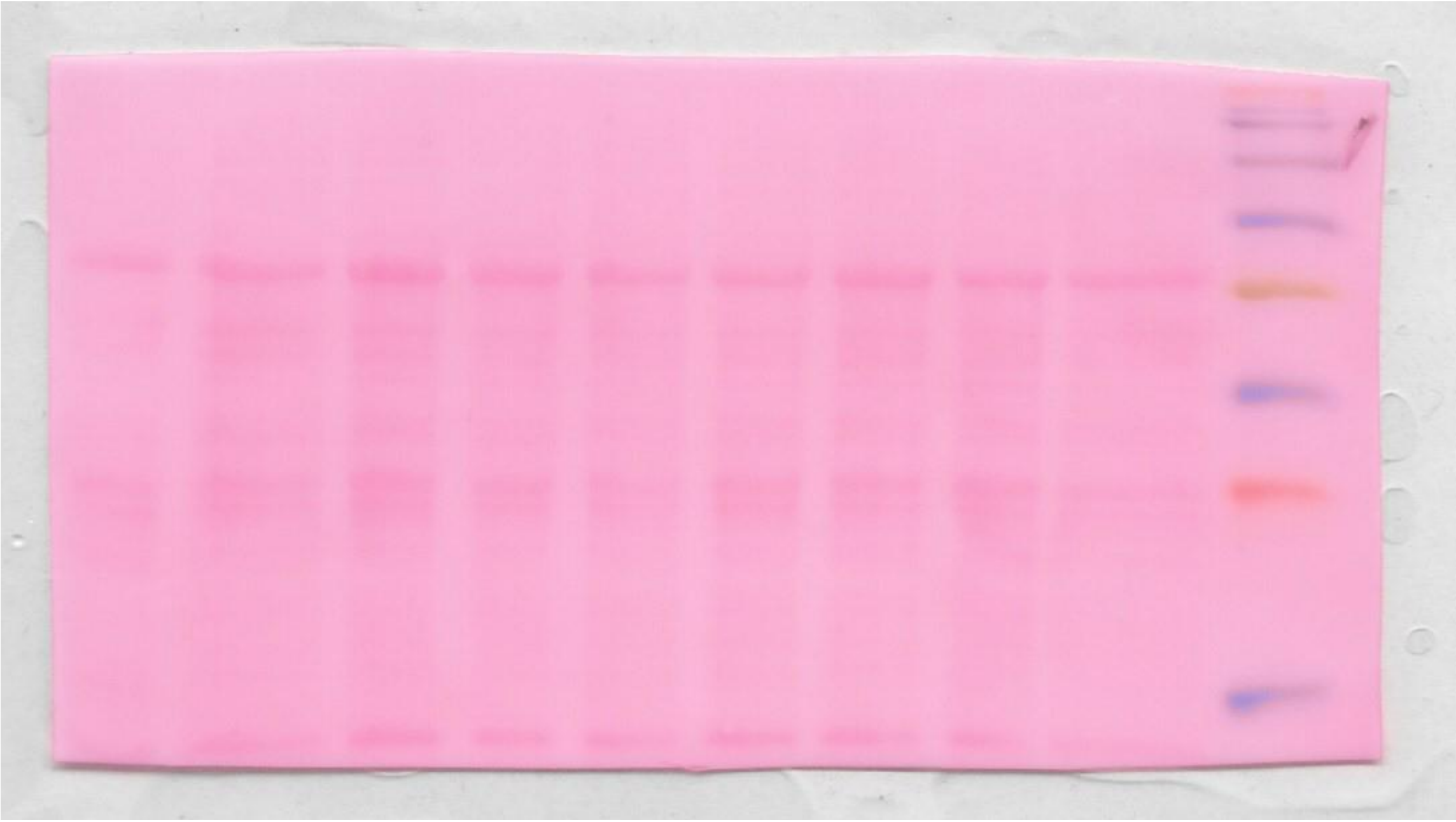

MANUSCRIPT FIGURE 5C WESTERN AND CHARTS

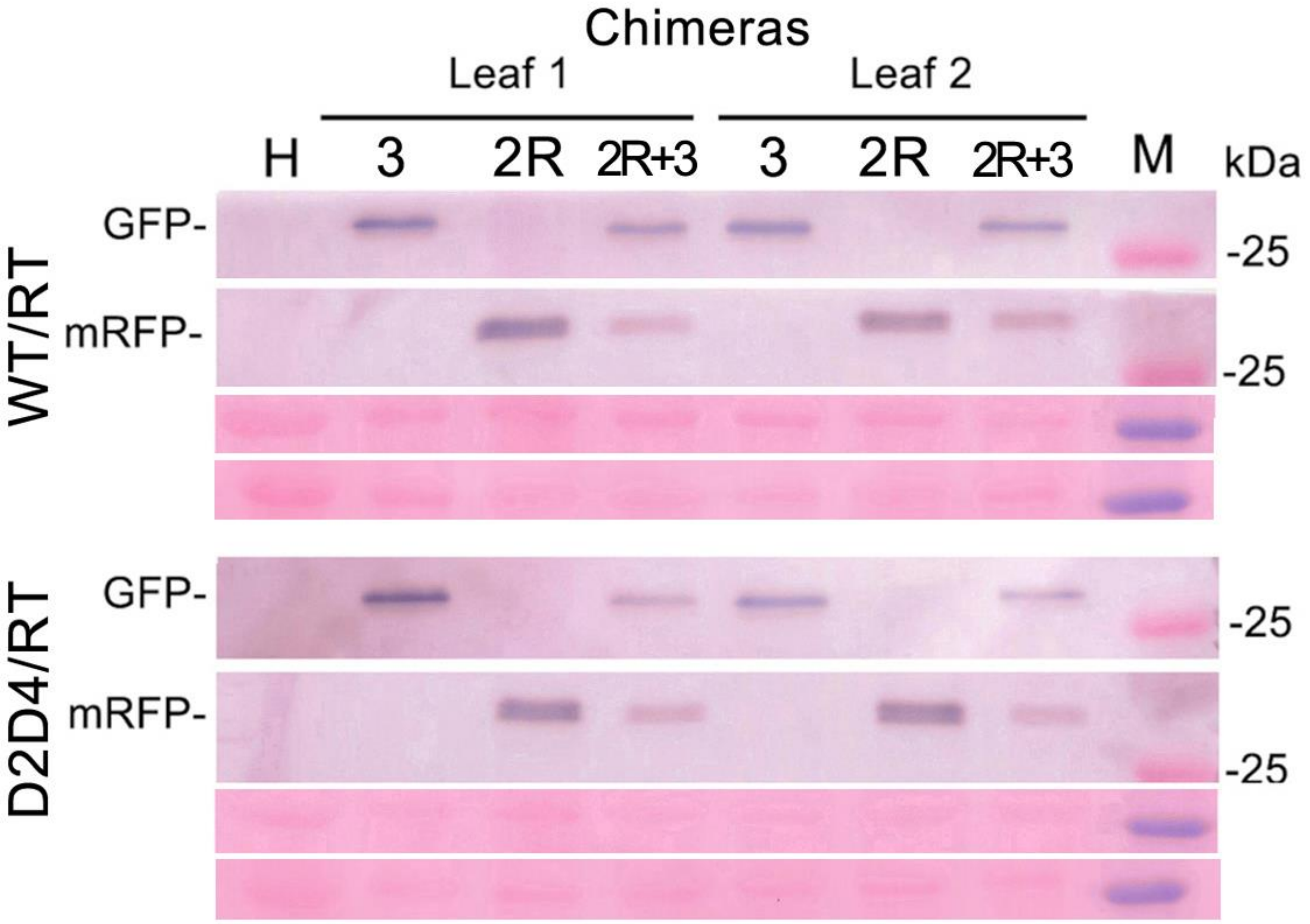

MANUSCRIPT FIGURE 5C WESTERN AND CHARTS

Western blots

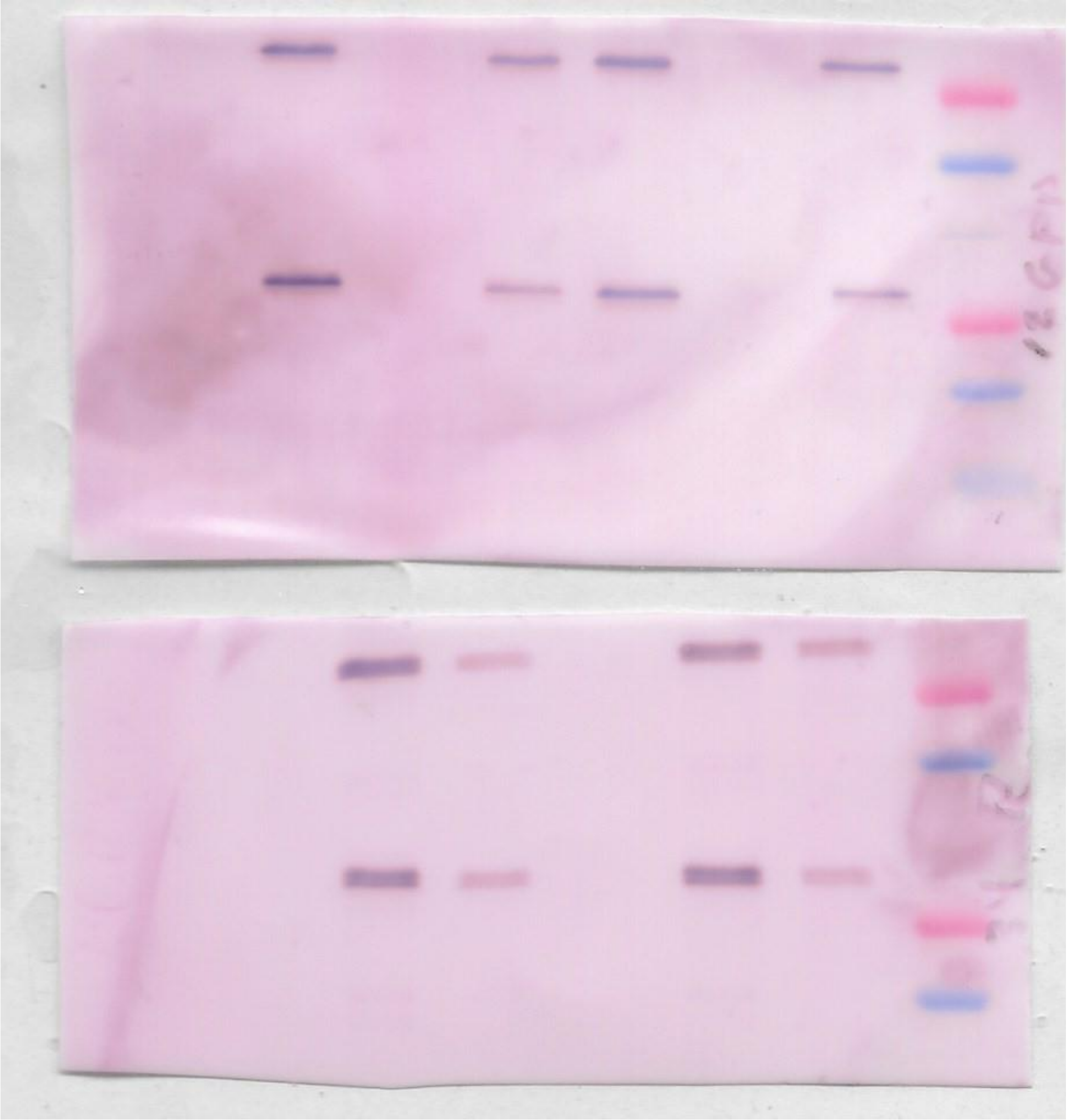

Ponceau-stained blots

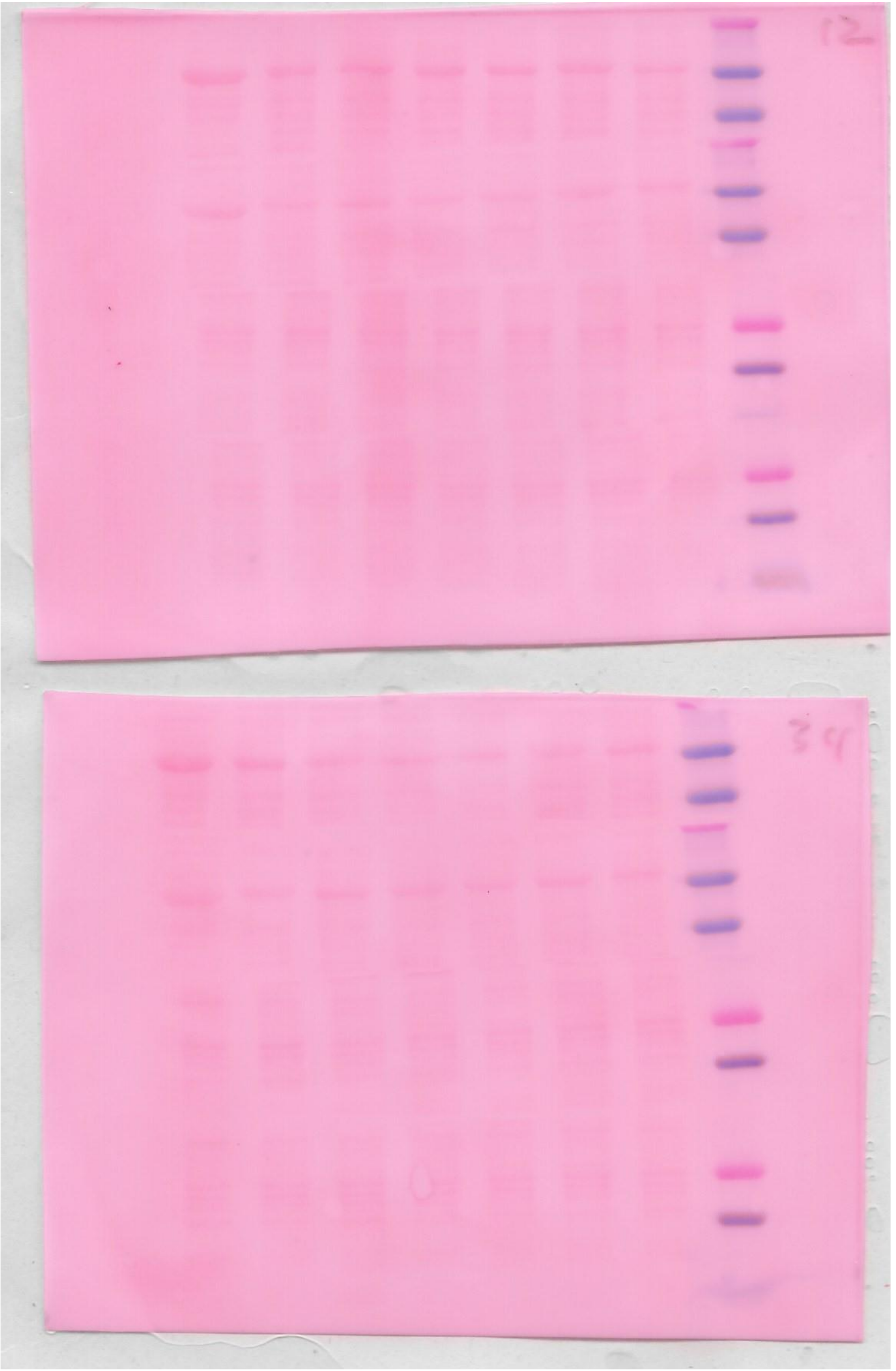

MANUSCRIPT FIGURE 5C CHARTS

Figure G: Chimera 2R and chimera 3, local, 5 dpi, WT or D2D4 plants,at RT

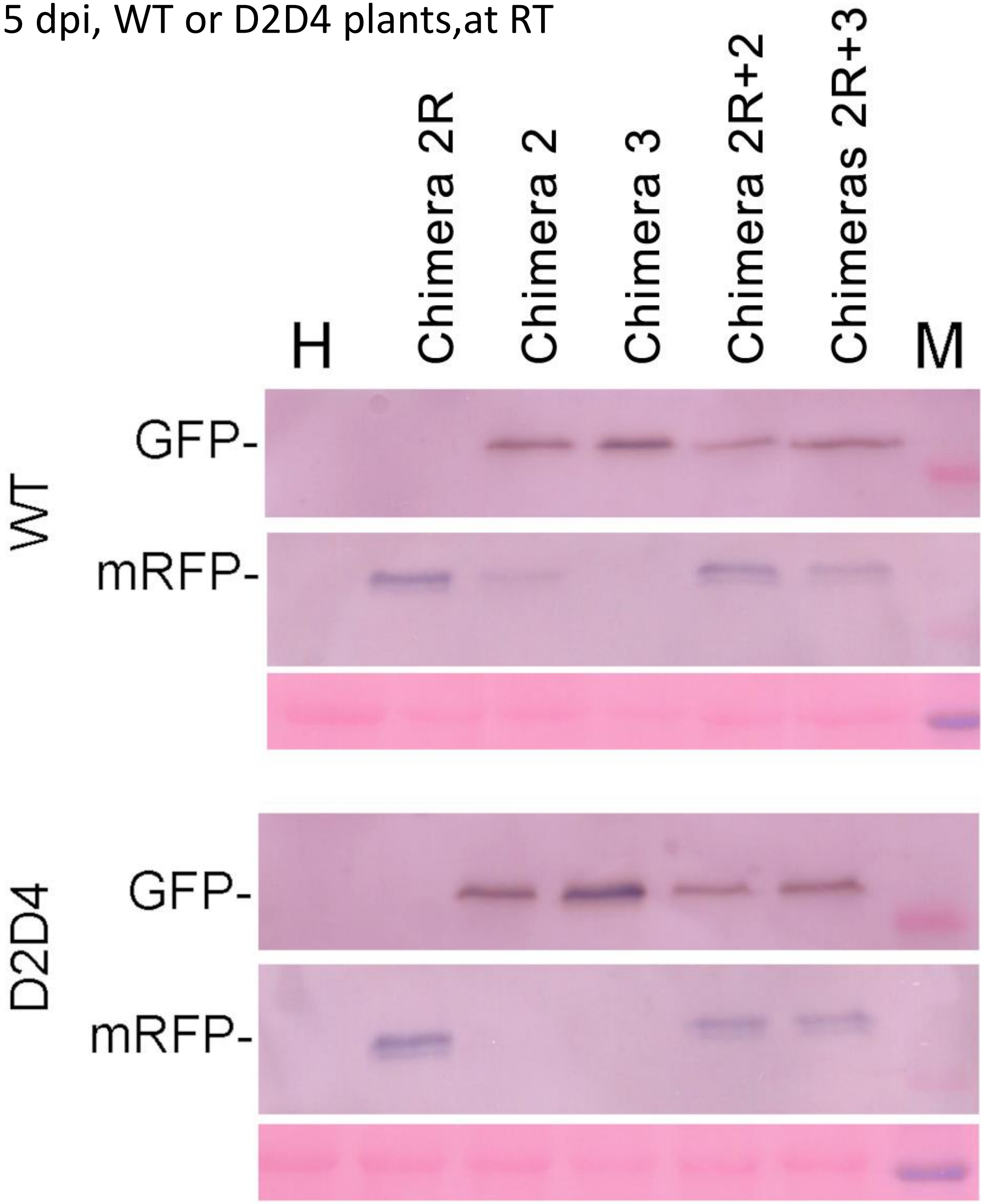

Raw blots for Figure G

Western blots

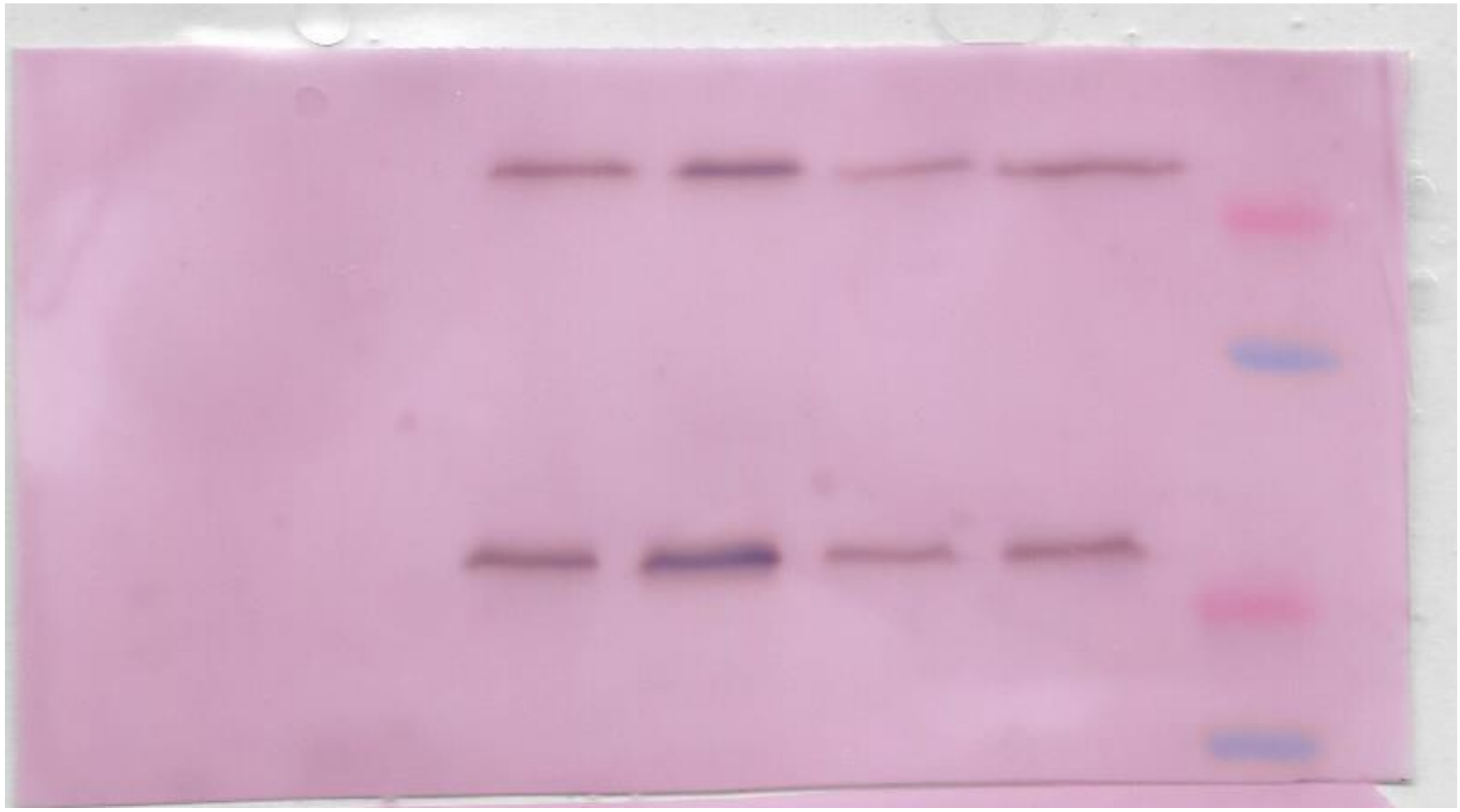

GFP

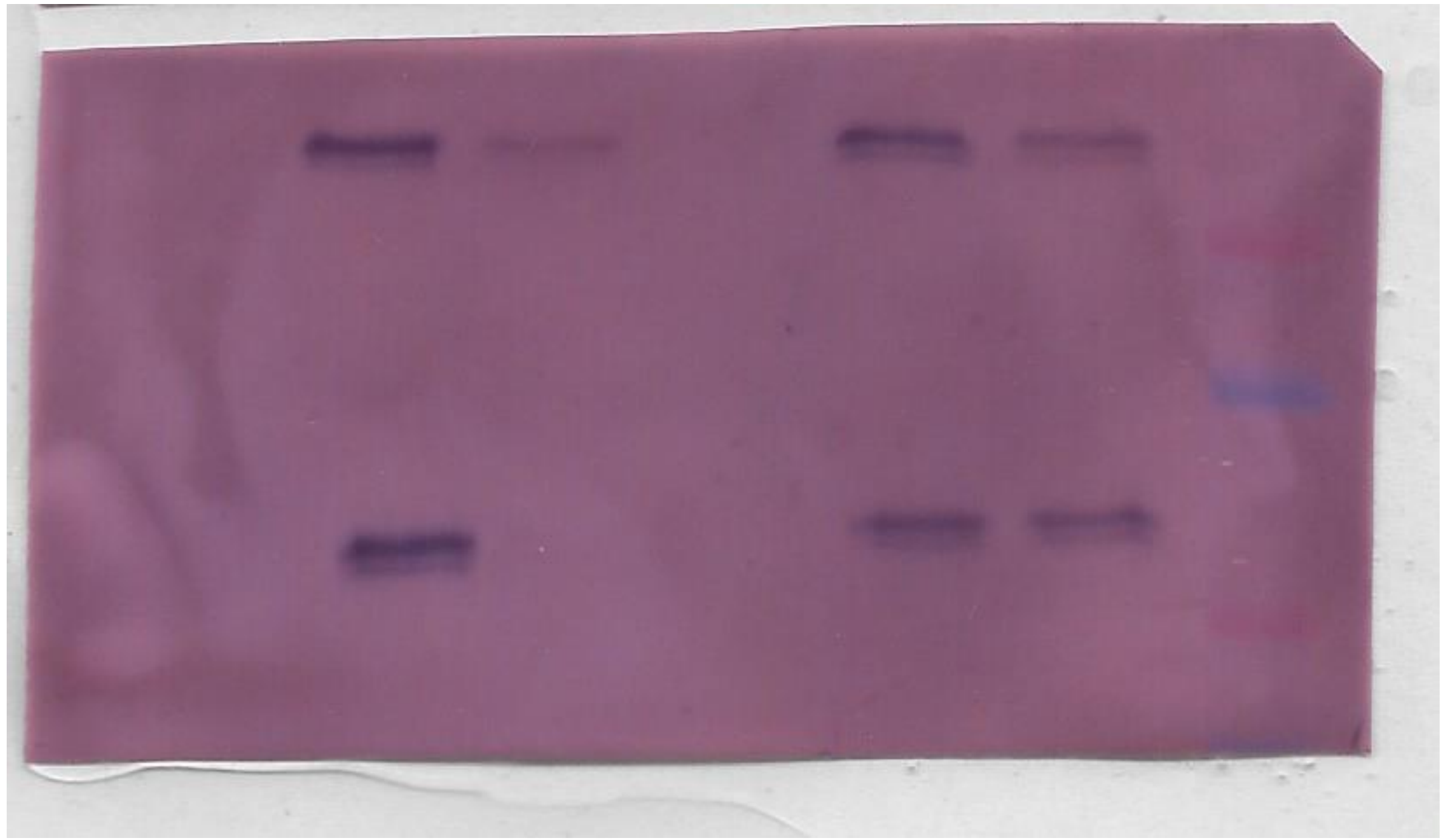

mRFP

Ponceau-stained blots

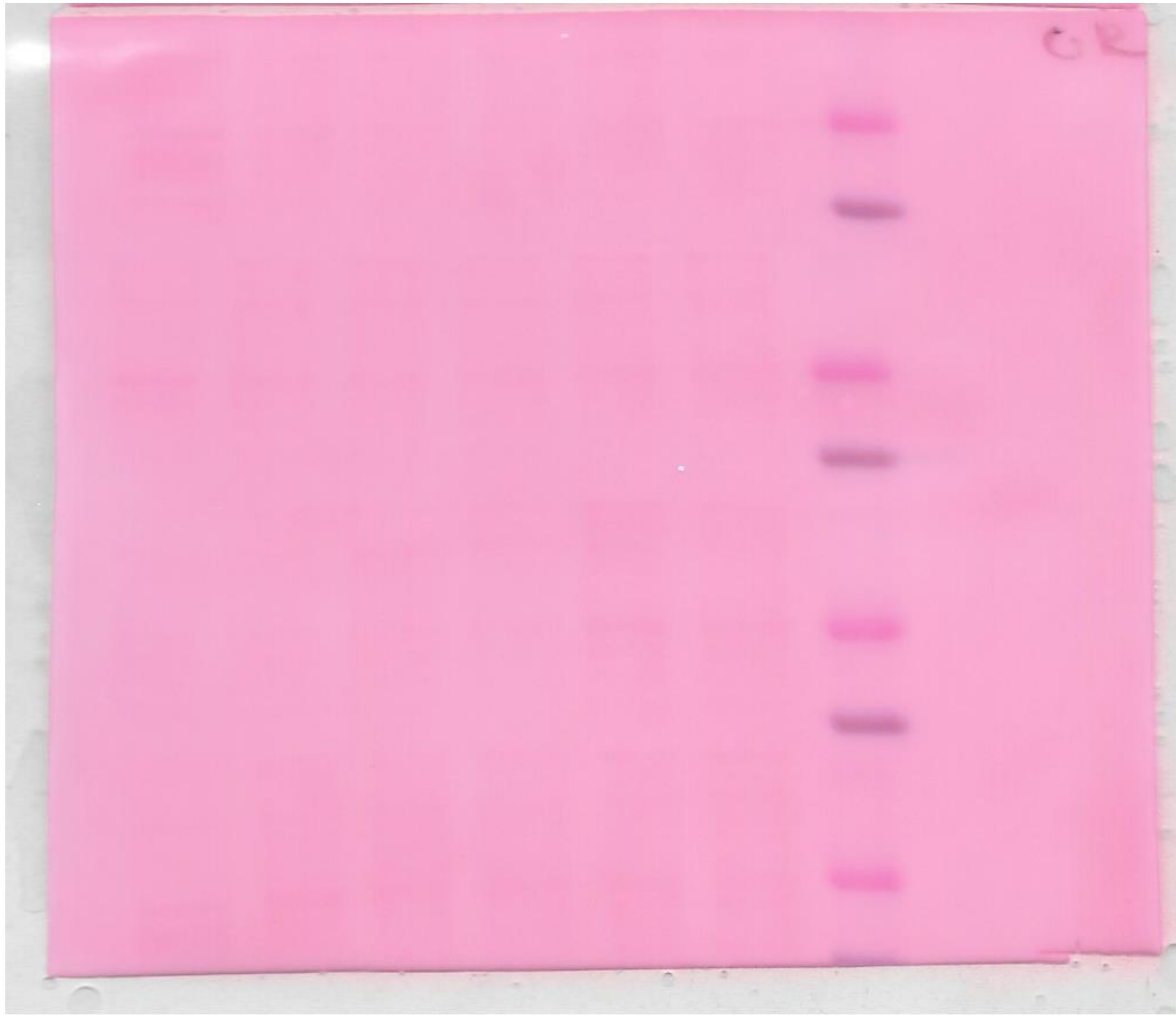

Western blot Figures used to obtain the chart shown in **MANUSCRIPT FIGURE 8B**

Figure H: Chimera 3 vs. chimera 5, local, 5 dpi, WT plants

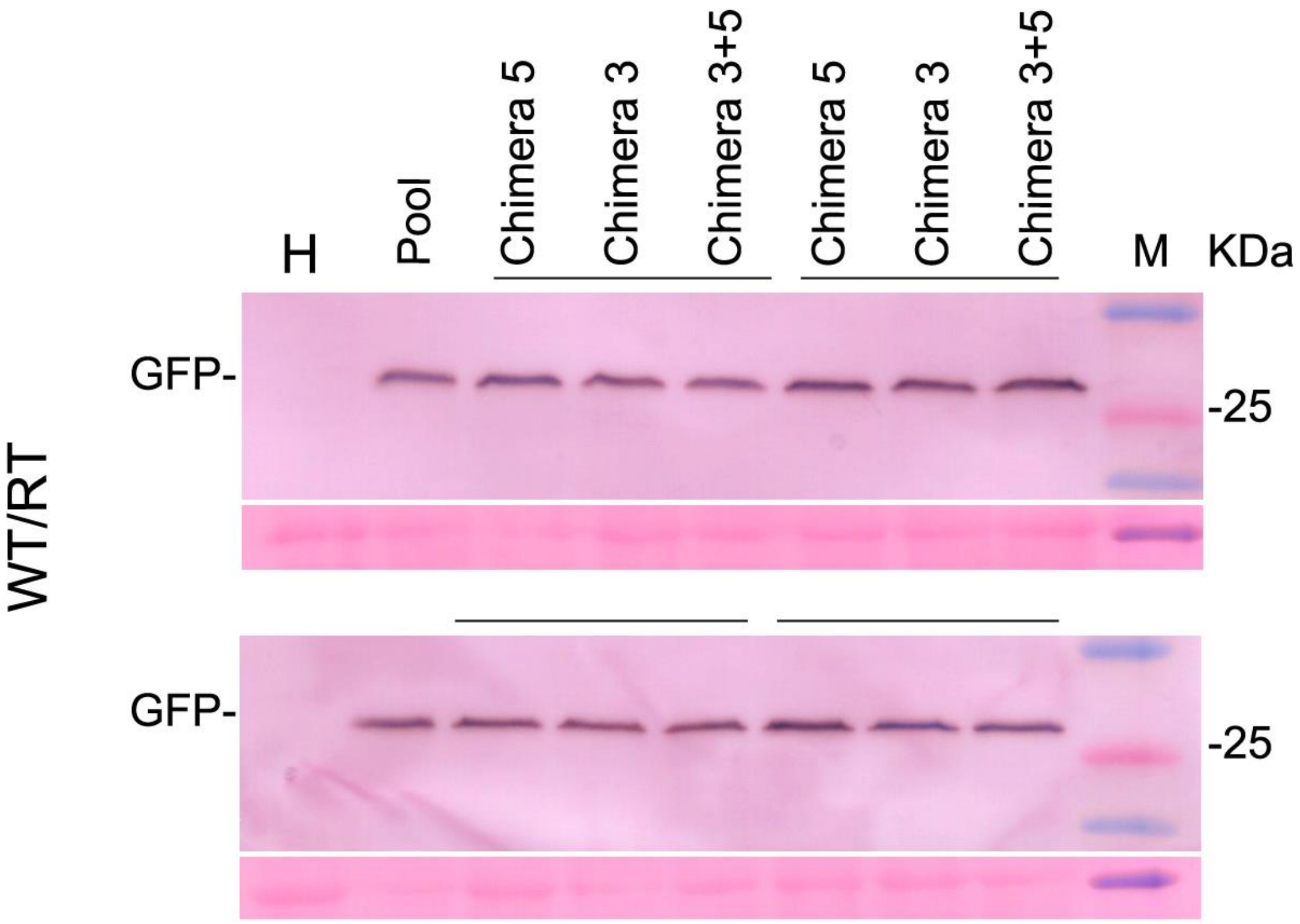

Raw blots for Figure H

Western blot

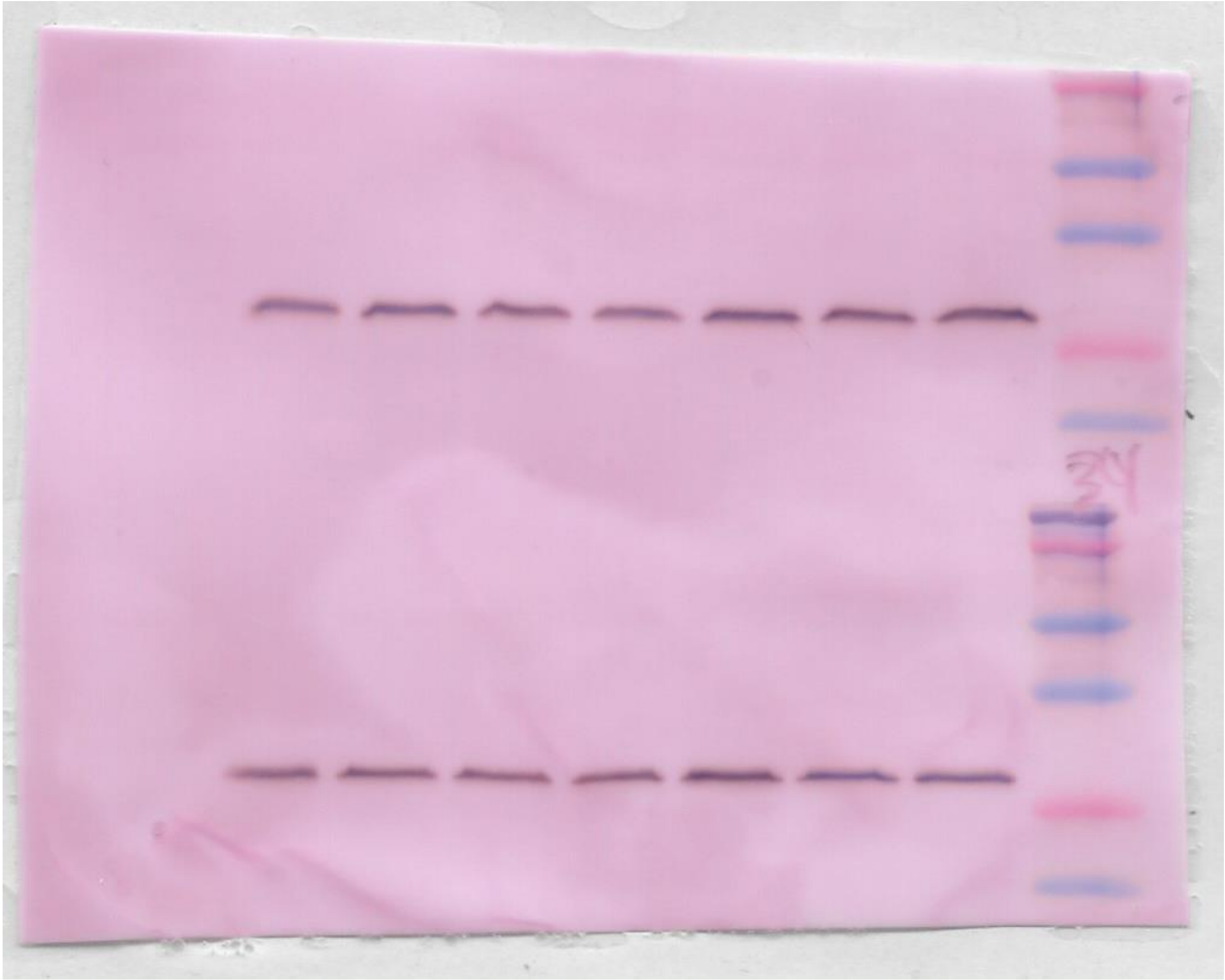

Ponceau-stained blot

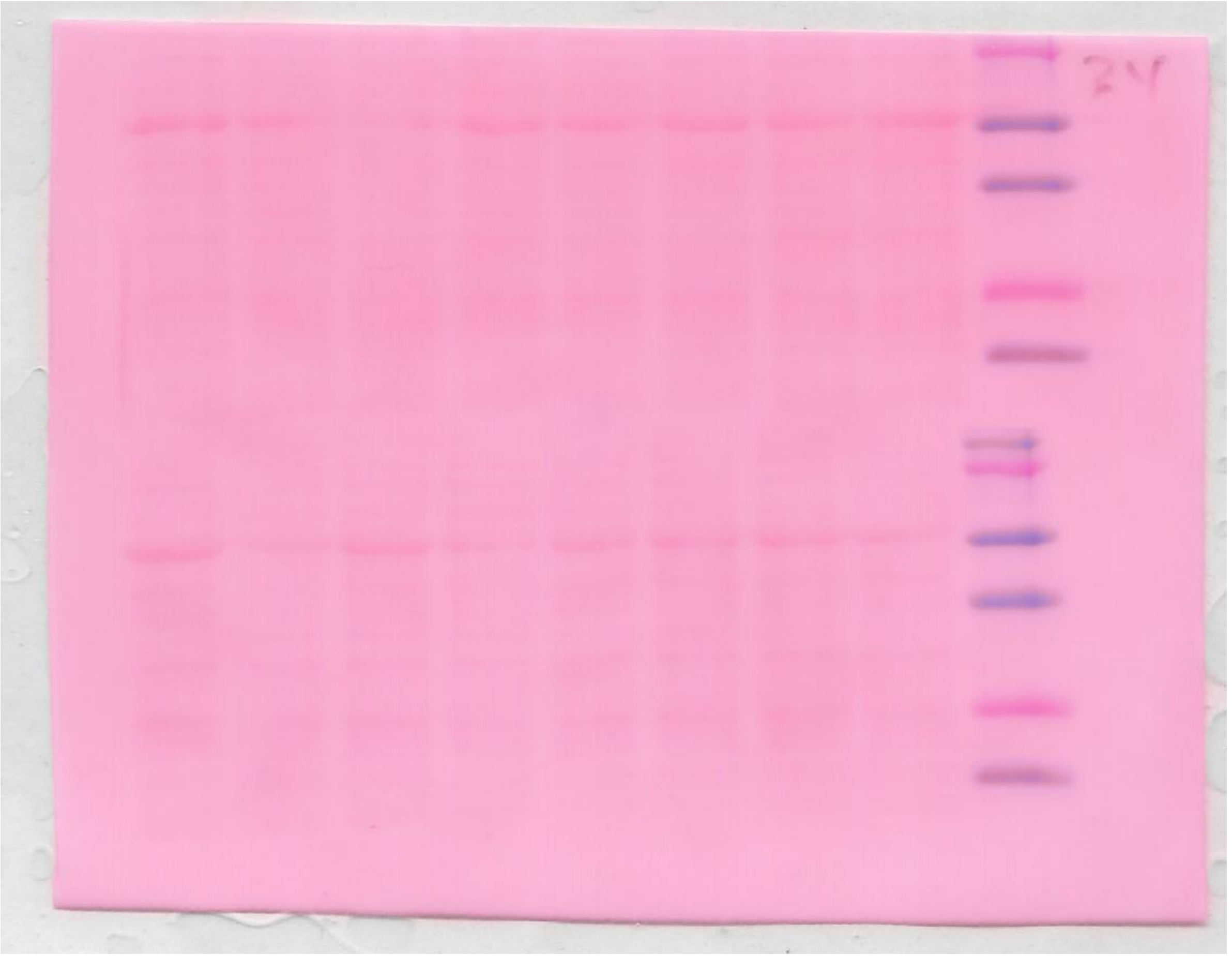

Western blot Figures used to obtain the chart shown in **MANUSCRIPT FIGURE 8B**

Figure I: Chimera 3 vs. chimera 5, local, 5 dpi, WT plants

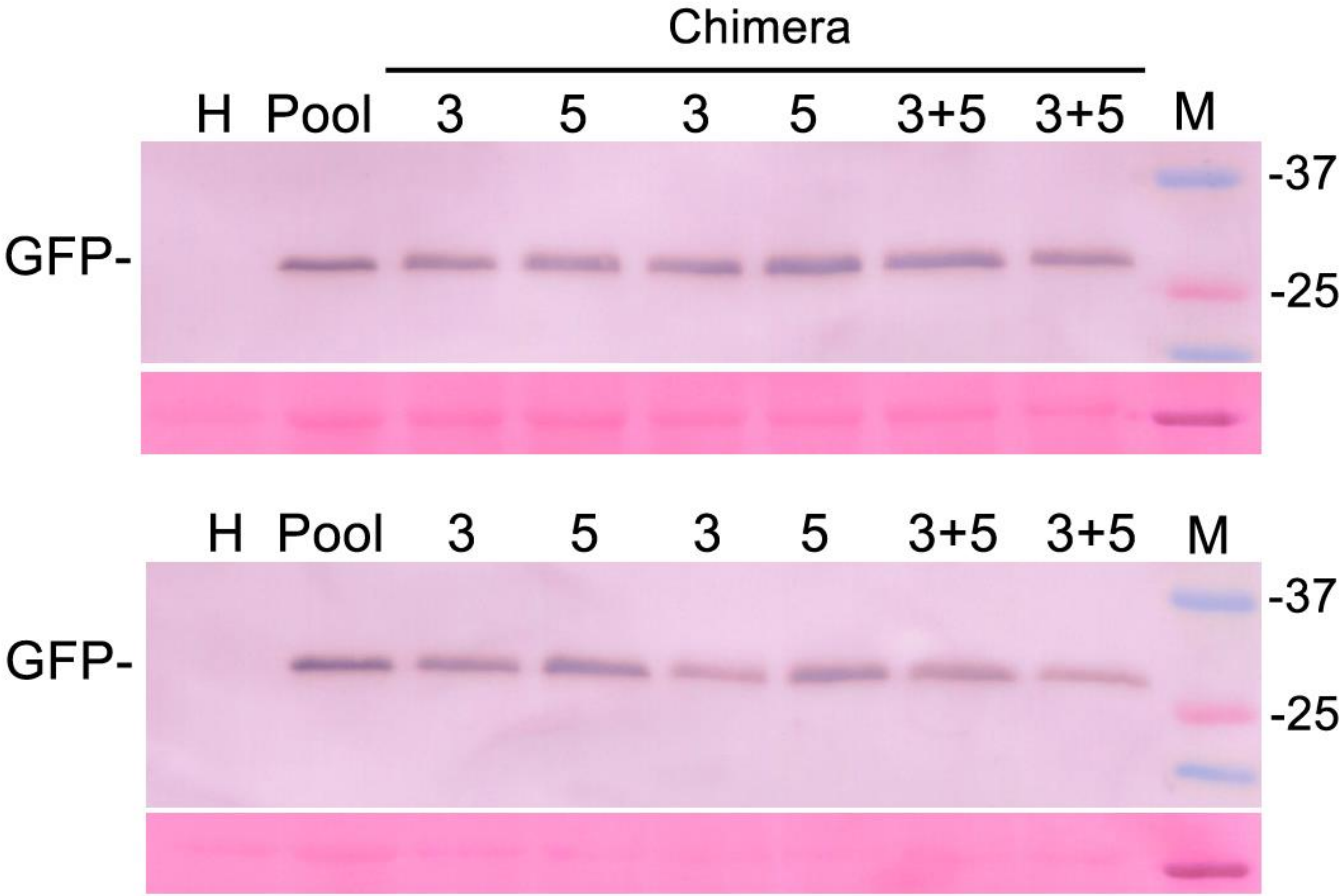

Raw blots for Figure I

Western blot

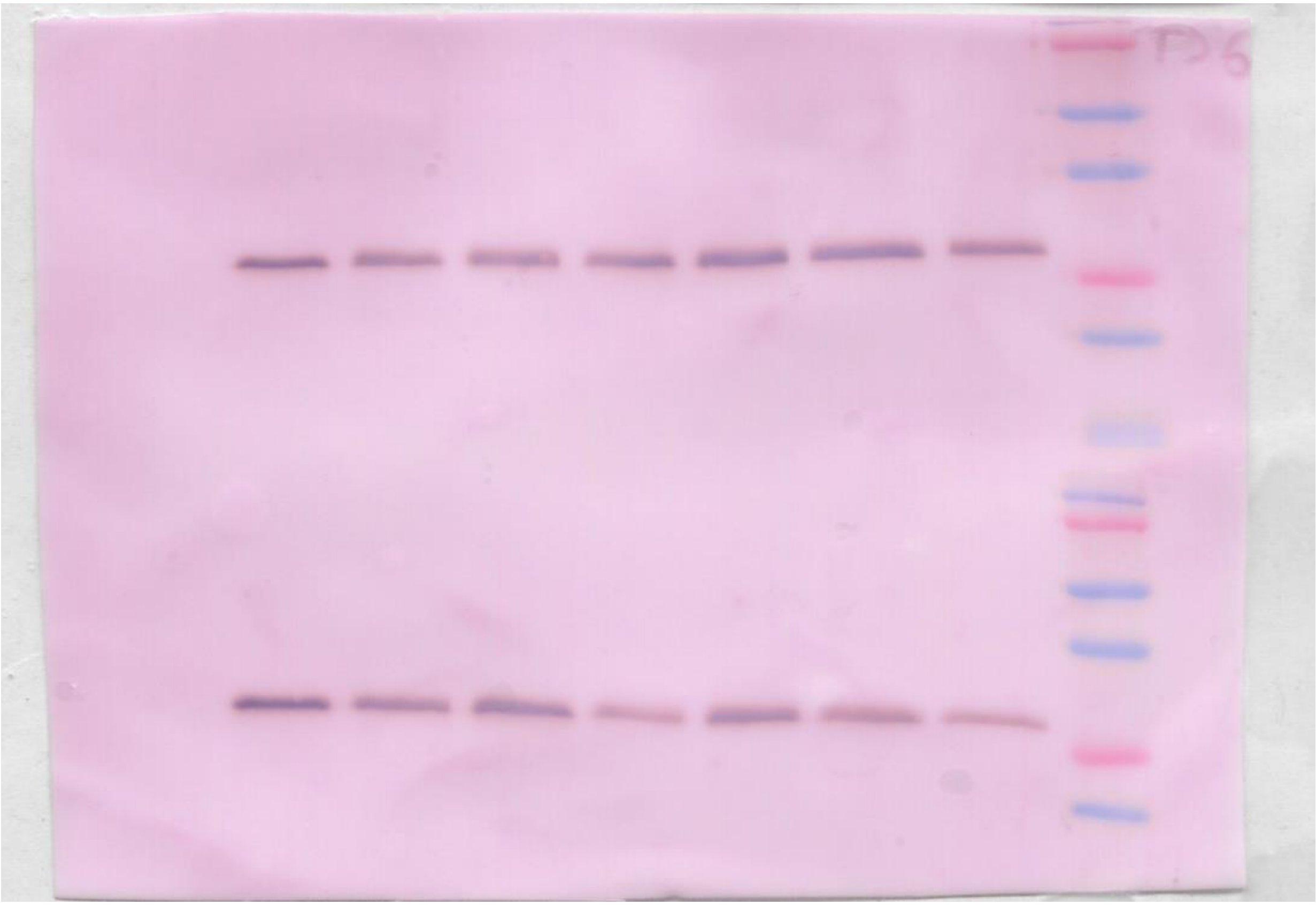

Ponceau-stained blot

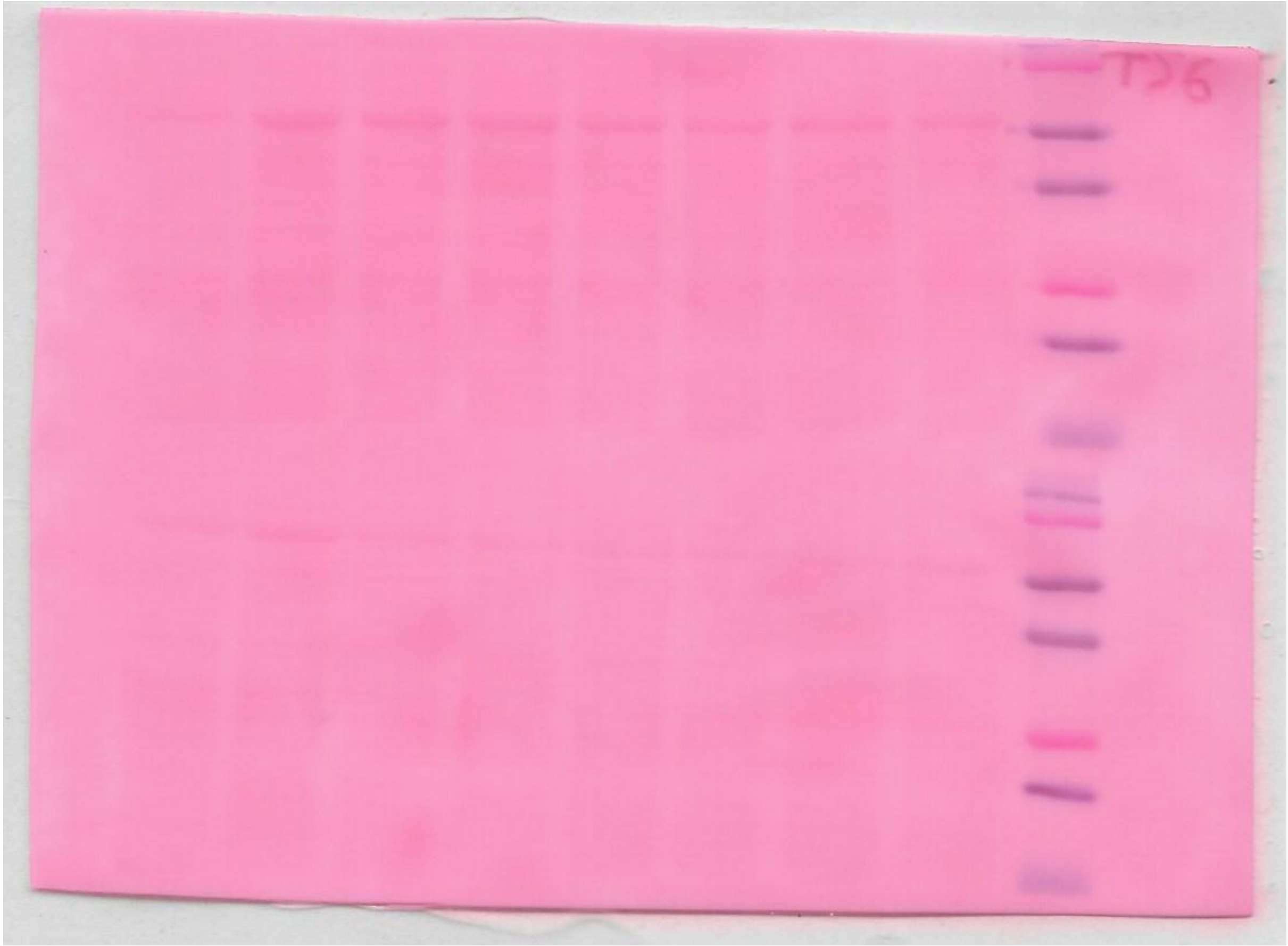

Western blot Figures used to obtain the chart shown in **MANUSCRIPT FIGURE 8B**

Figure J: Chimera 3 vs. chimera 5, local, 5 dpi, WT plants

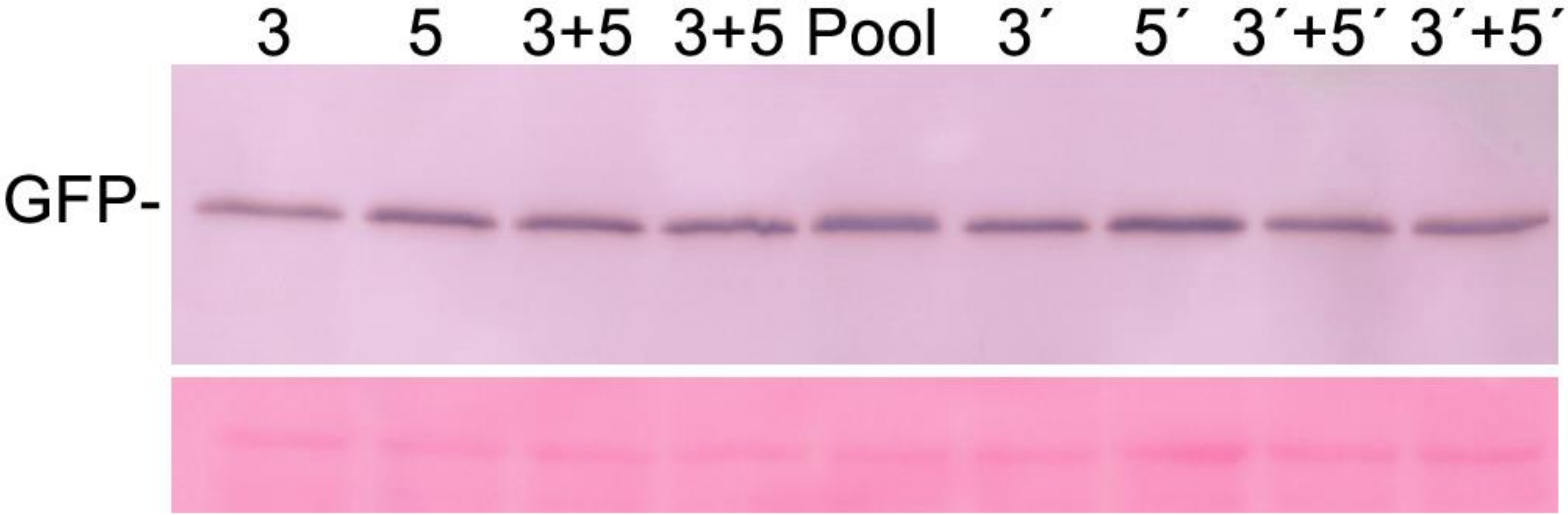

Raw blots for Figure J

Western blot

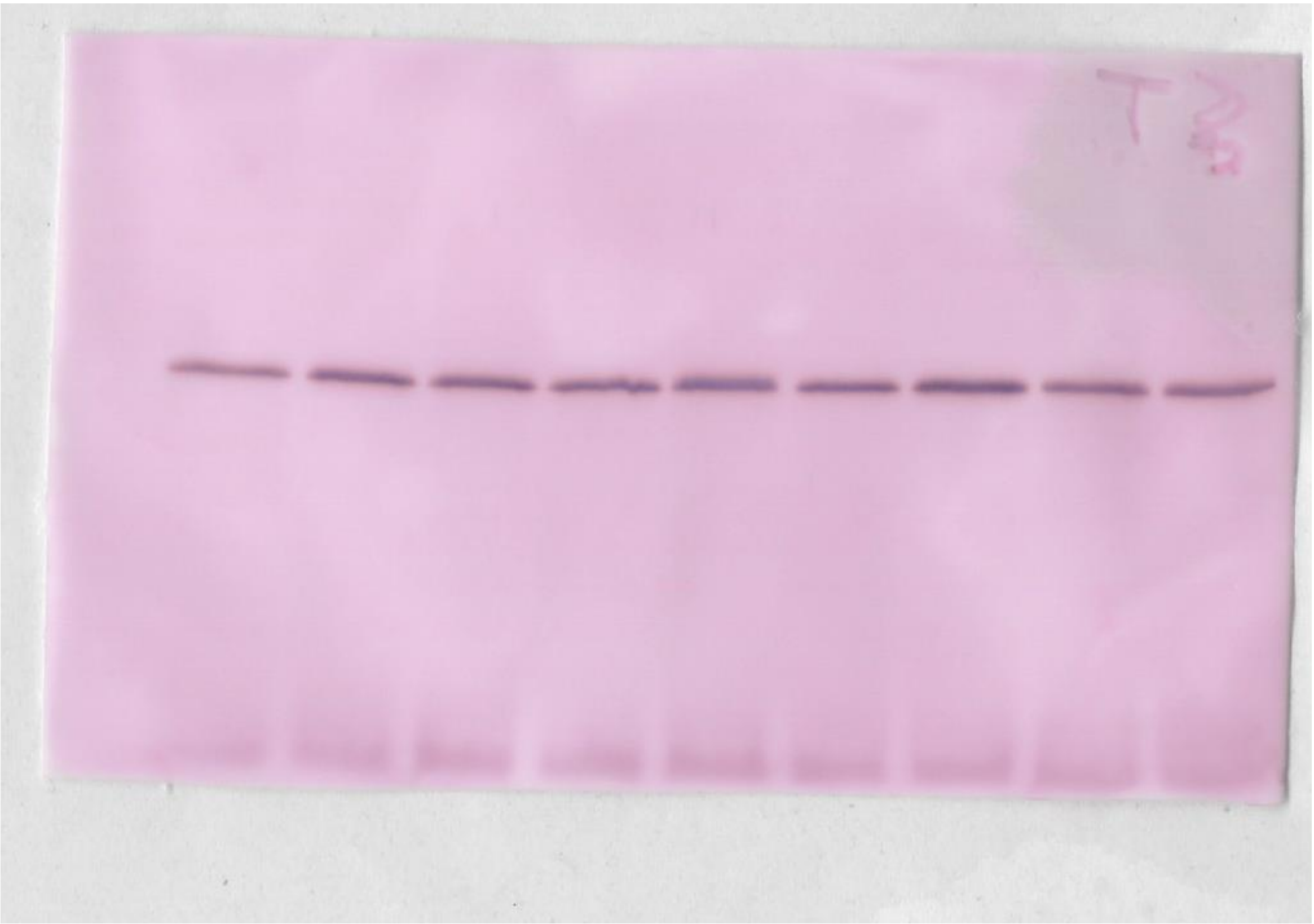

Ponceau-stained blot

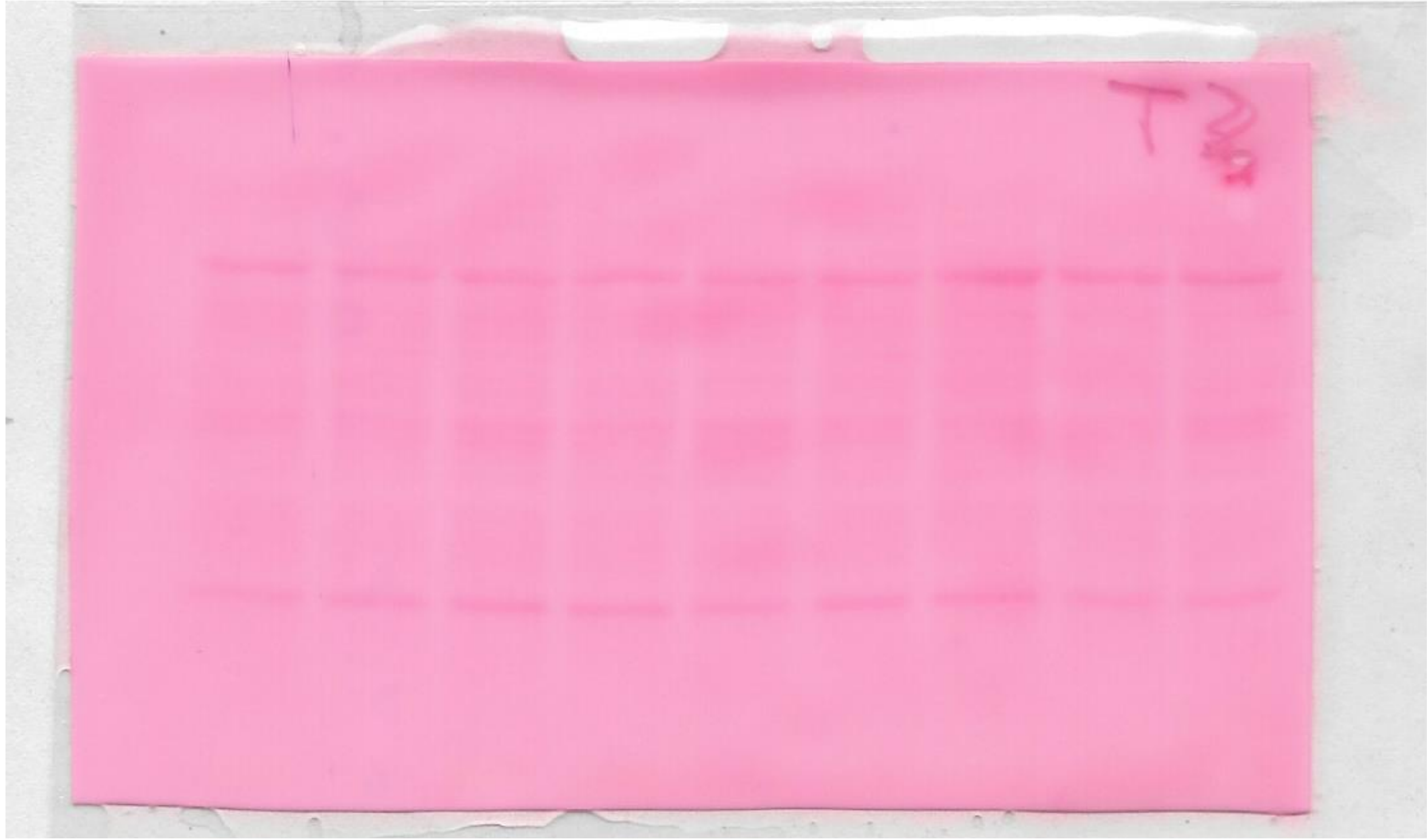

MANUSCRIPT FIGURE 8D

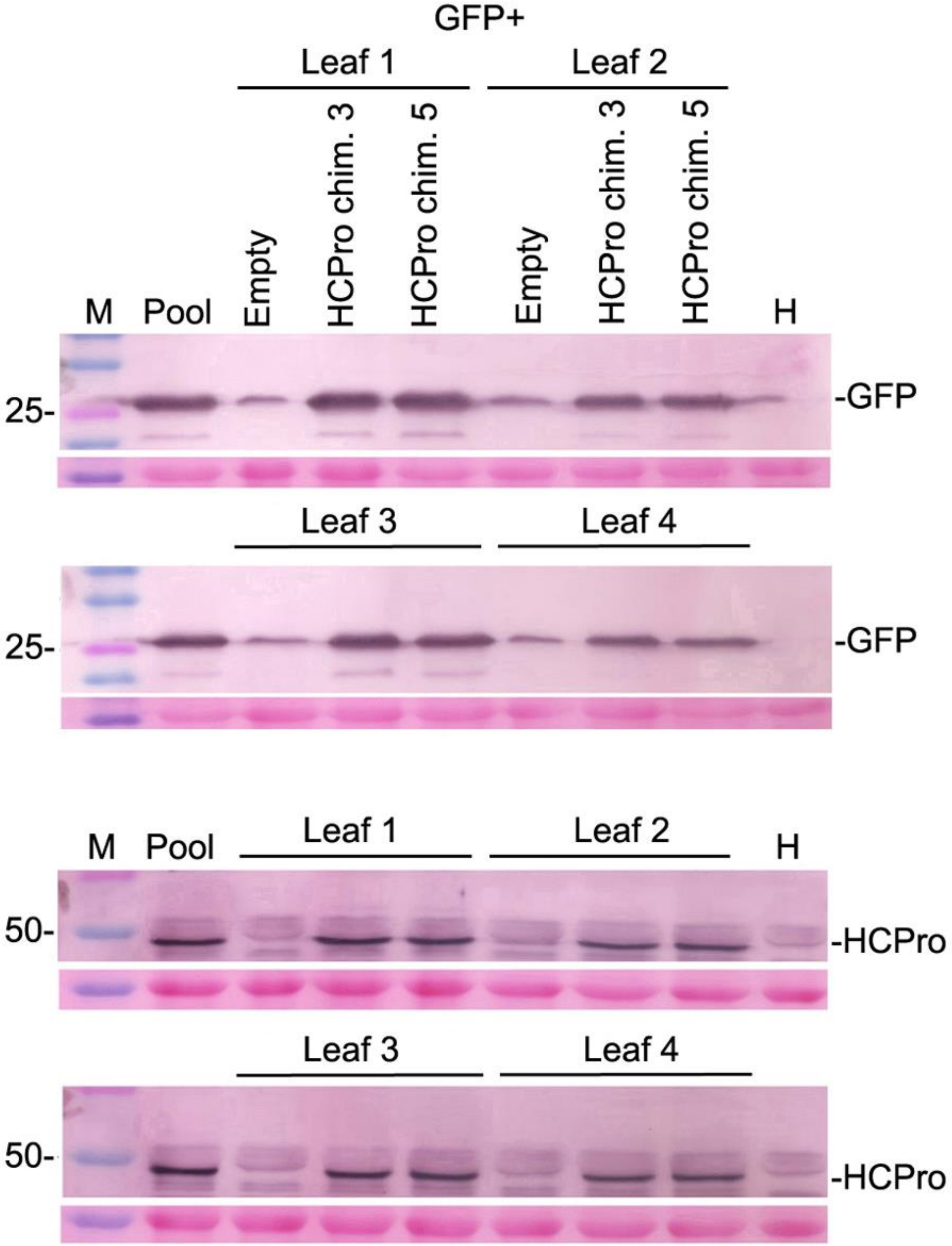

Raw blots for **MANUSCRIPT FIGURE 8D**

GFP

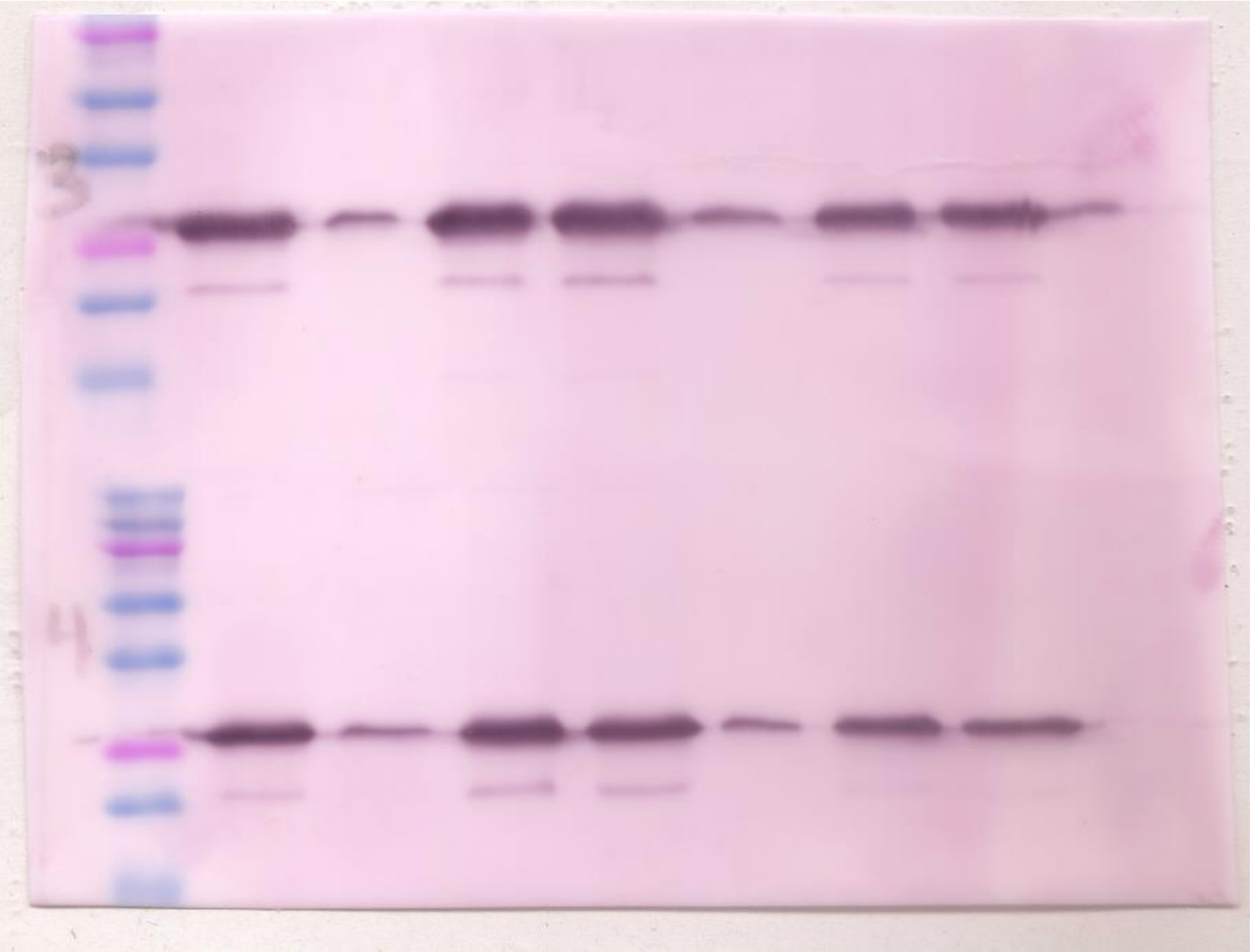

HCPro

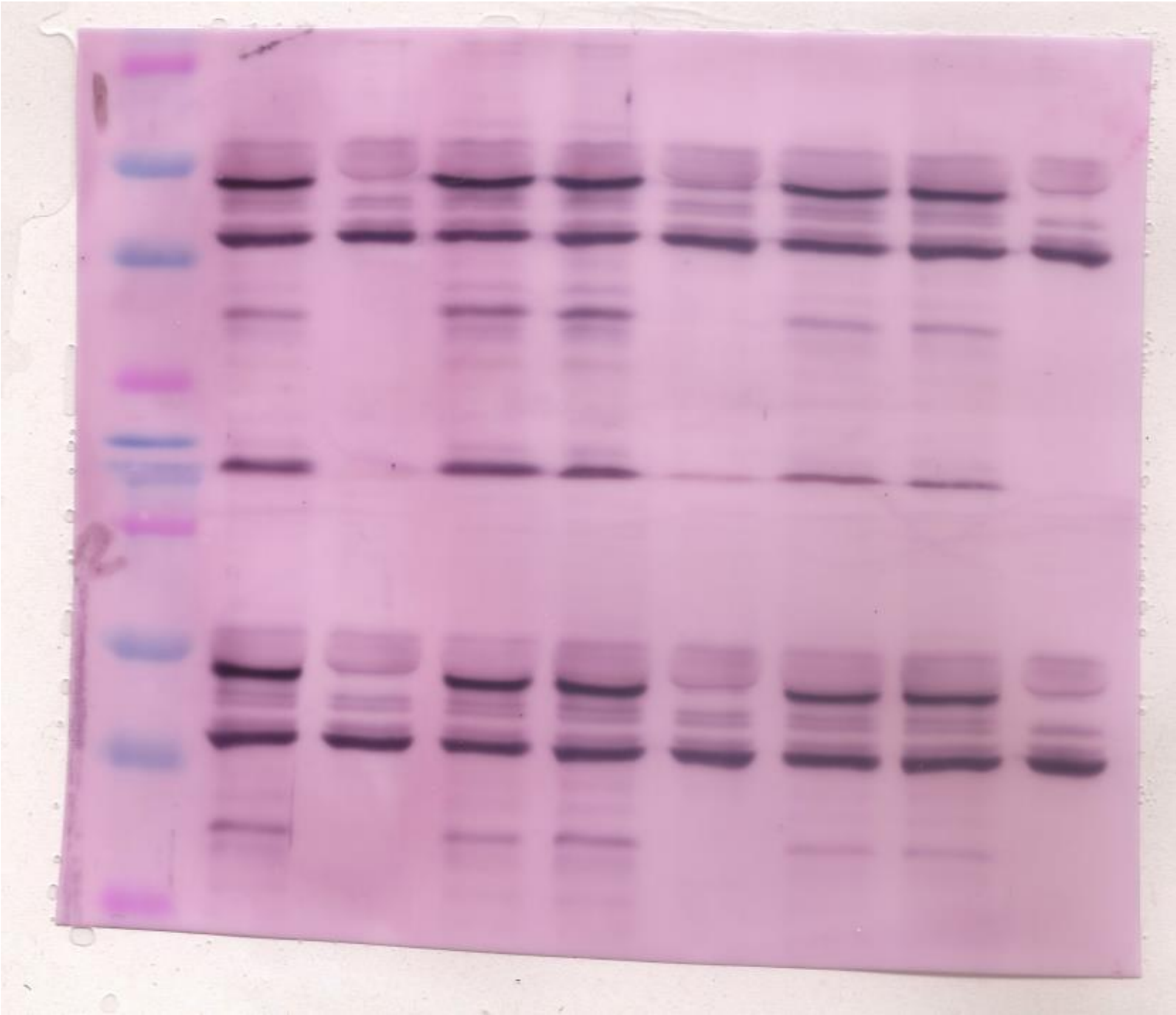

Western blots

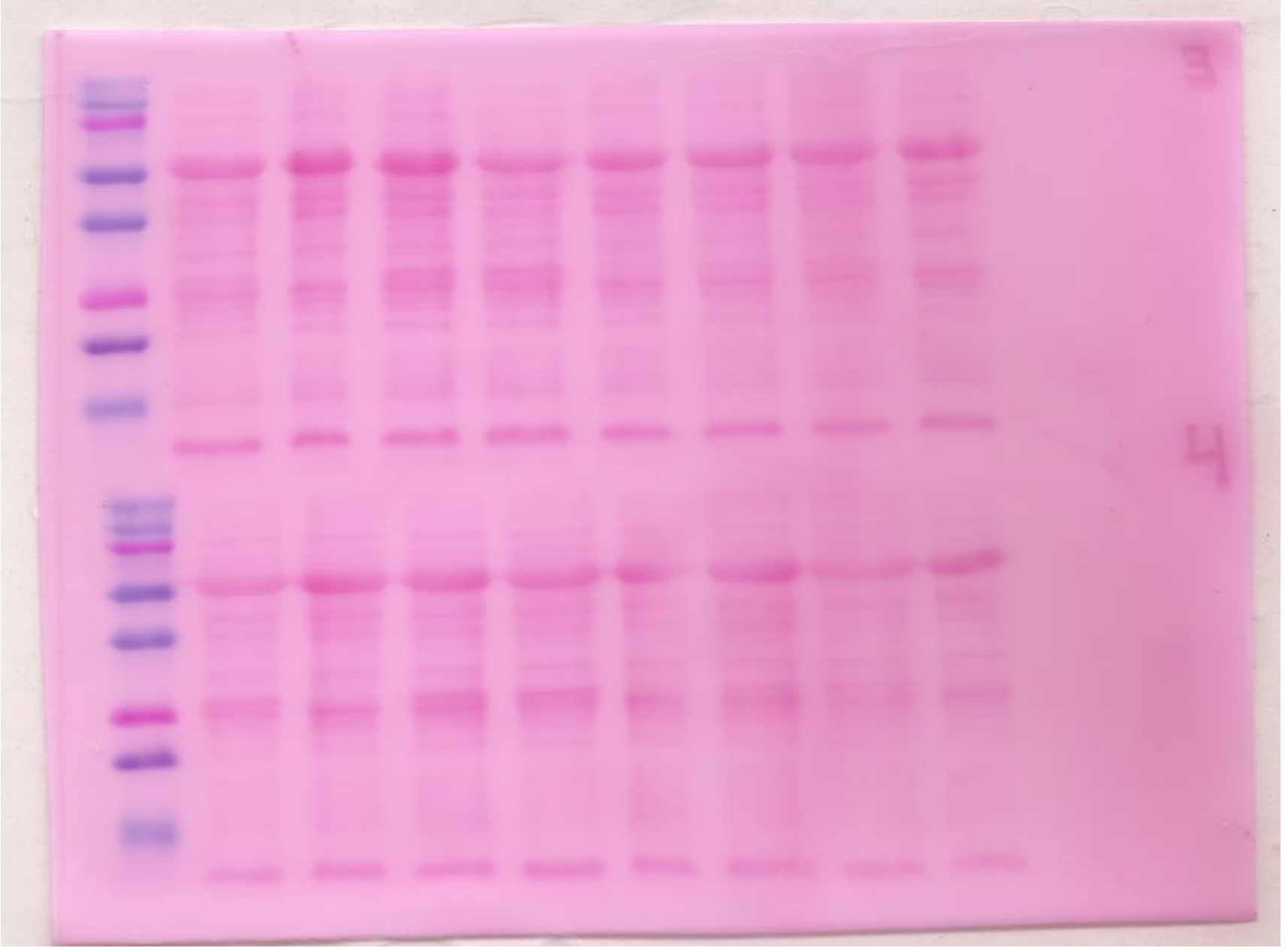

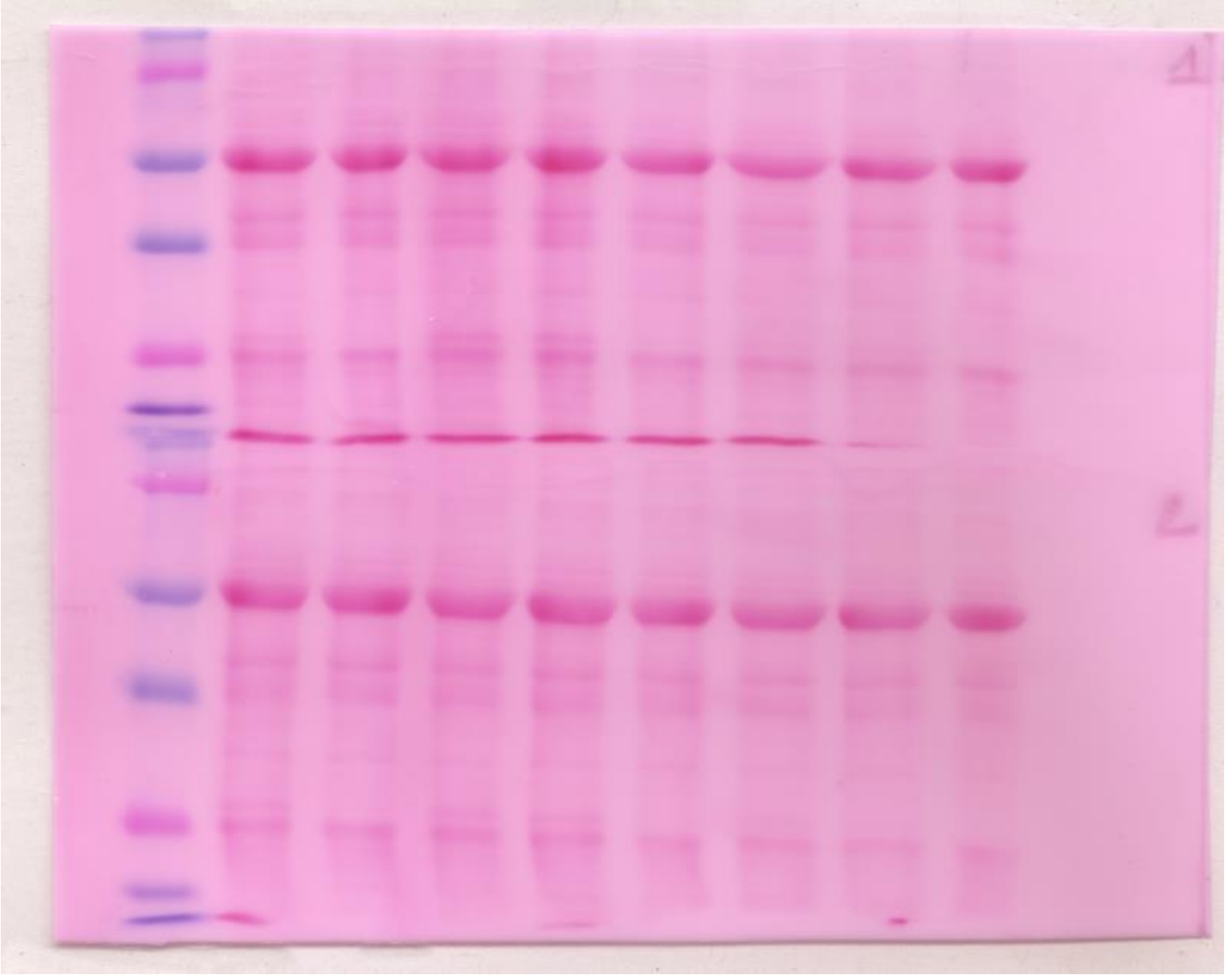

Ponceau-stained blots

NUMBER OF INDIVIDUAL AGROPATCH REPLICATES (n):

MANUSCRIPT FIGURE 2A

		n
WT/RT	Chimera 1	12
	Chimera 2	6
	Chimeras 1+2	7
		n
WT/RT	Chimera 1	12
	Chimera 3	6
	Chimeras 1+3	8
		n
WT/RT	Chimera 3	8
	Chimera 4	4
	Chimeras 3+4	8

MANUSCRIPT FIGURE 2B

		n
WT/RT	Chimera 2	4
	Chimera 3	8
	Chimeras 2+3	8
D2D4/RT	Chimera 2	4
	Chimera 3	8
	Chimeras 2+3	8
WT/HT	Chimera 2	8
	Chimera 3	8
	Chimeras 2+3	8

MANUSCRIPT FIGURE 8B

		n
WT/RT	Chimera 3	10
	Chimera 5	10
	Chimeras 3+5	12
