# Supplementary material for: Competition among co-infecting potyviral chimeras with adaptive, single-nucleotide differences in HCPro: Properties, and influence of environment factors on outcomes
Source: PLoS Pathog. 2026 Aug 18;22(8):e1014529. doi: 10.1371/journal.ppat.1014529 (PMC13502915; doi:10.1371/journal.ppat.1014529)
Supplement: Supplementary file 2 [file ppat.1014529.s003.docx]

**S1 Table. Additional mutations detected by nanopore sequencing in systemic infections**

| **Position** | | **Changes** | **Frequency /Depth** | | | | | |
| --- | --- | --- | --- | --- | --- | --- | --- | --- |
| **nt** | **aa** | ***HCPro*** | **Control*** | | **D2D4/RT** | | **WT/HT** | |
|  |  |  | **Freq.** | **Depth** | **Freq** | **Depth** | **Freq** | **Depth** |
| 51 | 17 | CT**G**(Leu)🡪CT**C**(Leu) | ND | | 0,04 | 108 | ND | |
| 232 | 78 | **G**AT(Asp)🡪**T**AT(Tyr) | 0,03 | 79 | ND | | ND | |
| 530 | 177 | T**C**T(Ser)🡪T**T**T(Phe) | 0,06 | 104 | 0,05 | 164 | 0,04 | 227 |
| 581 | 194 | T**C**G(Ser)🡪T**A**G(STOP) | ND | | ND | | 0,03 | 262 |
| 581 | 194 | T**C**G(Ser)🡪T**G**G(Trp) | ND | | ND | | 0,03 | 262 |
| 646 | 216 | **C**GG(Arg)🡪**T**GG(Trp) | ND | | ND | | 0,03 | 280 |
| 775 | 259 | **G**AA(Glu)🡪**A**AA(Lys) | ND | | 0,03 | 213 | ND | |
| 1152 | 384 | GC**G**(Ala)🡪GC**T**(Ala) | 0,04 | 226 | ND | | 0,05 | 580 |
| 1155 | 385 | AC**C**(Thr)🡪AC**G**(Thr) |  |  | ND | | 0,03 | 583 |
| 1170 | 390 | AT**G**(Met)🡪AT**C**(Ile) | ND | | ND | | 0,03 | 608 |
| 1218 | 406 | TT**G**(Leu)🡪TT**A**(Leu) | ND | | 0,03 | 345 | ND | |
| 1221 | 407 | GT**C**(Val)🡪GT**T**(Val) |  |  | 0,03 | 353 | 0,04 | 634 |
| 1222 | 408 | **G**AC(Asp)🡪**T**AC(Tyr) |  |  | 0,03 | 354 | 0,03 | 645 |
| 1234 | 412 | **C**AA(Gln)🡪**G**AA(Glu) | ND | | ND | | 0,03 | 664 |

ND: not detected

*: nanopore reads from ds cDNA obtained from combined single chimera 2 and single chimera 3 local infections, at 5 days post-infiltration, in WT/RT conditions (shown in **Fig 4B**).
