# Supplementary material for: Competition among co-infecting potyviral chimeras with adaptive, single-nucleotide differences in HCPro: Properties, and influence of environment factors on outcomes
Source: PLoS Pathog. 2026 Aug 18;22(8):e1014529. doi: 10.1371/journal.ppat.1014529 (PMC13502915; doi:10.1371/journal.ppat.1014529)
Supplement: Supplementary file 3 [file ppat.1014529.s005.docx]

**S1_Text.** **Commands and scripts followed for the analysis of nanopore data**

- Raw FASTQ files were quality-filtered using NanoFilt software (v2.8.0) with a minimum Phred score threshold of 20 (Q20).
- To isolate viral sequences, reads were mapped against the indexed HCPro reference sequence using minimap2 software (v2.30-r1287). Only reads with robust viral alignment were retained. The alignment process yielded several hundred viral reads per sample.
- Downstream analyses to determine chimera ratios combined automated quantification using custom Python 3 scripts and manual inspection via IGV (Integrative Genomics Viewer, v2.19.7). This step allowed for manual confirmation of the chimera ratios identified by the automated analysis.
- Although the **R10.4.1 (V14)** chemistry achieves a raw accuracy exceeding 99% (**Q20**), a minimum detection threshold of **3%** was applied for minority variant identification.

**Detection of chimera ratios in co-infections:**

1-Filter results to Q20 average:

NanoFilt -q 20 < results.fastq > q20_filtered.fastq

**A-Samples co-infected with chimera 1 and 2; 1 and 3; and 3 and 4:**

1-Reference

Reference is 105 nucleotides around the mutation

Position of mutation=53

🡪For samples co-infected with chimeras 1 and 2; and 3 and 4, triplets of amino acid (aa) 352 were analyzed:

**Reference352:** tacattgccaagcagggttattgttatattaacgtgtttcttgcaatgctg**att**aacattagcgaggaggatgcaaaggatttcacaaagaaagttcgcgacatg

🡪For samples co-infected with chimeras 1 and 3, triplets of aa 454 were analyzed:

**Reference454:**

caacttatcttgtttgcaaatgatgaattagaatctgatataaaacattac**cgg**gttggaggtcttgaagtggataagtgtgacgaattcaaaaatgtcaagctc

2-Indexation of reference

minimap2 -x lr:hq -k 11 -w 5 -d ref.mmi reference.fasta

3-Alignment and Indexation

minimap2 -ax lr:hq -t 4 ref.mmi q20_filtered.fastq | \

samtools sort -o alignement.bam

samtools index alignement.bam

4-Extraction of viral reads in separete fasta files

samtools fasta -F 2052 alignement.bam > viral-reads.fasta

5- Check the ratios of the chimera pairs

samtools mpileup -r "reference352:53-53" alignement.bam | cut -f5 | tr 'a-z' 'A-Z' | fold -w1 | grep -E "C|T" | sort | uniq -c

or:

samtools mpileup -r "reference454:53-53" alignement.bam | cut -f5 | tr 'a-z' 'A-Z' | fold -w1 | grep -E "T|G" | sort | uniq -c

**B-Samples co-infected with chimeras 2 and 3:**

1-Select sequence of reference:

Reference = coding sequence between the two triplets for aa 352 and 454

>reference

tgttatattaacgtgtttcttgcaatgctgattaacattagcgaggaggatgcaaaggatttcacaaagaaagttcgcgacatgtgtgtgccgaagcttggaacctggccaactatgatggatttggcgaccacttgtgctcaaatgagaatattctatcctgacgtgcatgatgcagaactgcctagaatattggtcgaccatgacactcaaacgtgtcatgtggttgactcatttggctcacaaacaactggatatcatattctaaaagcatccagcgtgtctcaacttatcttgtttgcaaatgatgaattagaatctgatataaaacattaccgggttggaggtcttgaagtggataagtgtgac

2-Index the reference:

Indexed one time this reference, and use to filter all the samples that are coinfections of chimeras 2 and 3

minimap2 -x lr:hq -k 15 -w 5 -d virus_ref.mmi reference.fasta

3-Alignment:

Align and ignore the sequences that are shorter than 307 bp (from aa triplet 352 to 454)

minimap2 -ax lr:hq -t 4 -m 307 virus_ref.mmi q20_filtered.fastq > alignement_filtre.sam

4-Extraction of viral reads:

samtools fasta -F 4 alignement_filtre.sam > viral_reads.fasta

5-Detection of chimera ratios:

nano chimeras_detection.py

from Bio import SeqIO

from Bio.Seq import Seq

from collections import Counter

refs = {

"c2352F": "CTAACATT",

"c2454F": "CATTACCT",

"c3352F": "TTAACAT",

"c3454F": "CATTACCG"

}

input_file = " viral_reads.fasta "

rc_refs = {k: str(Seq(v).reverse_complement()) for k, v in refs.items()}

stats = Counter()

output_data = {

"chimera1": ("c3352F", "c2454F"),

"chimera2": ("c2352F", "c2454F"),

"chimera3": ("c3352F", "c3454F"),

"chimera4": ("c2352F", "c3454F")

}

files = {name: open(f"{name}.fasta", "w") for name in list(output_data.keys()) + ["tocheck"]}

def check_match(sequence, s1, s2):

fwd = (refs[s1] in sequence) and (refs[s2] in sequence)

rev = (rc_refs[s1] in sequence) and (rc_refs[s2] in sequence)

return fwd or rev

print(f"Analysis of {input_file}...")

for record in SeqIO.parse(input_file, "fasta"):

seq_str = str(record.seq).upper()

found_category = "tocheck"

for cat, (p1, p2) in output_data.items():

if check_match(seq_str, p1, p2):

found_category = cat

break

SeqIO.write(record, files[found_category], "fasta")

stats[found_category] += 1

for f in files.values(): f.close()

print("\n--- Results---")

for cat in ["chimera1", "chimera2", "chimera3", "chimera4", "tocheck"]:

print(f"{cat.ljust(10)} : {stats[cat]} sequences")

print(f"{'TOTAL'.ljust(10)} : {sum(stats.values())} sequences")

CTL+O then ENTER

CTL+X

Run:

python3 chimeras_detection.py

The “tocheck” file is then analysed with IGV (Integrative Genomics Viewer) software.

**Analysis of new mutations**

1-Filter results to Q20 average:

NanoFilt -q 20 < results.fastq > q20_filtered.fastq

2-Search of viral reads using sequence of wild-type *HCPro* cistron as reference

minimap2 -ax map-ont -k 12 --secondary=no HCPRO.fasta q20_filtered.fastq > aln_test.sam

samtools view -bh aln_test.sam | samtools sort -o aln_test.bam

samtools flagstat aln_test.bam

3- Filter MAPQ 20 after alignment

samtools view -bh -q 20 aln_test.bam > virus_Q20.bam

4-Indexation

samtools index virus_Q20.bam
