# Supplementary material for: Competition among co-infecting potyviral chimeras with adaptive, single-nucleotide differences in HCPro: Properties, and influence of environment factors on outcomes
Source: PLoS Pathog. 2026 Aug 18;22(8):e1014529. doi: 10.1371/journal.ppat.1014529 (PMC13502915; doi:10.1371/journal.ppat.1014529)
Supplement: Supplementary file 4 [file ppat.1014529.s007.docx]

**Part I – Summary**

**Reviewer #1:** This study analyzes the effects of single and co-infections of two natural variants, each harboring a single-amino acid mutation, on viral titers. It also provides insights into the possible biological significance of these mutations in response to biotic and/or abiotic stresses. While the authors tackle a complex subject and employ two different methods to assess mutant abundance, the evidence provided remains insufficient. Furthermore, the analysis is restricted exclusively to the HCPro sequence, leaving it unclear whether unexamined mutations exist elsewhere in the viral genome. Consequently, the current data do not fully **substantiate the authors’ conclusions**.

We thank reviewer 1 for the comments. The manuscript studies how co-infecting variants of a potyviral chimera with single amino acid differences in HCPro (Sun et al., 2024) interact with each other in local infections. This includes the assessment of overall viral titers and variant ratios.

In systemic tissues, our assessment is limited to determining which co-infecting variant prevails, in separate individual plants. This was done through sequencing of the *HCPro* region that harbored the differences between variants. Sequencing of this region allowed us to detect that in a few plants the prevalent variant had also incorporated novel mutations in the *HCPro* cistron (Figure 7). We describe them in the manuscript because we found them, and because we will be able to assess by reverse genetics their effect on virulence, and in some of them, on HCPro functions. In our lab we have developed through time expertise on different means to assay some of the multiple functions of HCPro. (i.e., two types of silencing suppression assays on transiently-expressed reporters; plant-to-plant or membrane-mediated aphid transmission tests; in planta proteolytic cleavage rates; intracellular relocation to microtubules of mRFP-tagged homodimers…). These assays could allow us to study effects of mutations on specific functions. We lack expertise on functional assays with other potyviral proteins. In this regard, we started assessing the effect of one of them (at amino acid 329) on the virulence of the virus + limited functional assays. It did not affect titers, prevalence in co-infections, or silencing suppression assays (Figure 8).

It was not an aim of this manuscript to find mutations in other parts of the viral genome, and we do not think that this affects our conclusions on local co-infection properties, or on systemic prevalence. We have gone through the manuscript to make sure our conclusions fit with this limitation.

We know that mutations in other genomic regions can occur. In fact, we found a couple in an earlier work, in addition to mutations at HCPro amino acids 352 and 454 of the chimera, after several months of infection and of mechanical passages, in the VPg/NIa and the CP, respectively, but the virus did not shed the *GFP* cistron (Sun et al., 2022).

**Reviewer #2:** This study systematically investigates the competition dynamics, environmental modulation, and further adaptive evolution of potyviral chimeras carrying single or double beneficial mutations in HCPro. The topic is theoretically significant, directly addressing classical questions about RNA virus quasispecies evolution, competitive exclusion, and co-infection dynamics. The work provides new experimental evidence for how plant RNA viruses **fine-tune** a multifunctional protein to adapt to environmental changes. However, several aspects require clarification, stronger evidential support, **or reinterpretation**.

We thank reviewer 2 for the positive view on our work, and have addressed the issues raised

**Reviewer #3:** This manuscript describes how isogenic potyviral chimeras, differing by one or two adaptive nucleotide substitutions in the HCPro, perform during local and systemic co-infection in *Nicotiana benthamiana* plants. I think that the rationale of this work is sound because HCPro is a multifunctional protein and whether beneficial mutations can act in trans on co-infecting genomes, or compete, and whether environmental conditions can alter the outcome is timely and very interesting. Moreover, it combines viral adaptation, within-host competition, environmental modulation, and the functional pleiotropy of HCPro.

We thank reviewer 3 for these comments on our work

**Part II – Major Issues: Key Experiments Required for Acceptance**

**Reviewer #1:**

As the authors acknowledge, RNA viruses exhibit a high mutation rate. Although the sequences of the HCPro region were verified across different treatments, this targeted sequencing cannot rule out the possibility that the **observed phenotypic differences** are driven by spontaneous mutations elsewhere in the viral genome. Additional experiments need to be carried out to verify this.

The manuscript studies local and systemic infections. With regard to the former, they were 5-day long, single infections or co-infections, in agro-patch leaf tissues. Phenotypic differences assessed were: the relative overall titers and variant ratios in co-infections, in some cases under different environments. We do not see how in this particular experimental design, within this time frame, in absence of cell-to-cell movement, any further mutation occurring in a variant in a particular cell could affect our overall conclusions, which were statistically assessed.

With regard to systemic co-infection experiments, the only phenotypic assessment was the determination of which, if any, of the co-infecting variants would prevailed in individual plants, under different ambient conditions, at 29 dpi (chimeras 2 + 3, 45 plants; chimeras 3 + 4, 16 plants). While it is possible that new mutations could incorporate elsewhere in the genome of variants systemically infecting some of the plants, the fact that variant prevalence signal was so strong makes very unlikely that they could affect the prevalence outcome: that chimera 3 prevailed in 45/45 plants in 2 + 3 co-infections; and chimera 4 in 15/16 plants, in 3 + 4 co-infections.

Using Western blot analysis to compare the accumulation levels of mutants tagged with either GFP or RFP is **methodologically flawed**. Because anti-GFP and anti-RFP antibodies inherently possess distinct binding affinities and detection sensitivities, the relative signal intensities of the protein bands cannot be directly correlated with the actual in planta accumulation levels of the respective viral mutants.

We agree with reviewer 1 comments on antibody affinity. It was not our intention to suggest that the affinities of the GFP or mRFP antibodies were comparable, but by our mistake it is something that we implied when we gave both, the levels of GFP and mRFP, the value of 1 in single infections, within the same chart in Figure 5C.

We did not intend to compare GFP with mRFP levels, but GFP (or mRFP) levels in single infections, with GFP (or mRFP) levels in co-infections. We have amended Figure 5D, so that charts for relative GFP or mRFP levels are now separate.

**Reviewer #2:**

1-Potential circularity in the core conclusion that “variants do not influence each other.”
The authors use Equations 2 and 3 to argue that variants replicate independently: expected co-infection titers calculated from single-infection titers and observed co-infection ratios match the observed co-infection titers. This reasoning is partially circular because the “observed co-infection ratio” used in Equation 2 is itself derived from sequencing data, but the core test should rely on Equation 3 (expected ratio from single titers) compared to actual sequencing ratios. The discrepancy in D2D4/RT conditions (Table 3: expected ratio 0.50/0.50 vs. observed ~0.40/0.60) actually demonstrates that the environment can break the “independence” assumption – a critical finding.

The authors should: clearly distinguish between “expected ratio” (from single titers) and “observed ratio” (from sequencing). Discuss why D2D4 conditions lead to deviation from the independent-replication model. Possibilities include altered relative accumulation rates of HCPro variants in a silencing-deficient background, as the authors briefly mention (lines 410–414).

We agree with the reviewer that proof of independence is provided by Equation (3), in which the expected proportions of the variants are derived solely from their titers in single infections and are then compared with the sequencing-derived proportions measured in co-infected plants. The close agreement between these expected and observed ratios supports the conclusion that the variants replicate largely independently.

Equation (2) is not used as proof of independence, but rather, to show that the measured co-infection titers are compatible with the relative abundances observed by sequencing, and the titers obtained in single infections.

We have altered the order of appearance of the two equations in the manuscript [equation (3) is now equation (2)].

The terms “expected ratio” (from single titers) and “observed ratio” (from sequencing) are standardized along the manuscript.

Regarding D2D4 plants, we have included a whole paragraph on this issue in the Discussion section. In it we indicate in it that in a previous study, we had shown that the transgenic background hampers the relative accumulation of HCPro variants carrying a Thr residue at position 352 during infection (Sun et al., 2024). One major difference between the two plant backgrounds may lie in the populations of 21- and 22-nt small RNAs (sRNAs) generated in response to viral infection or to transcripts derived from agroinfiltrated T-DNA (Katsarou et al., 2019). PVY HCPro is known to bind 21–22 nt sRNAs, through mechanisms that remain unclear, as part of its silencing suppression activity (del Toro et al., 2018). Analysis of the accumulation of the four HCPro variants during local infection at 5 dpi in both plant backgrounds revealed that, in D2D4 plant, the accumulation of HCPro variants containing Thr at position 352 was markedly reduced. This sensitivity of HCPro to the sRNA environment suggests that interactions with sRNAs are important during infection or may contribute to stabilization of the protein. Consequently, the effect of the transgenic background on mixed infection may result from the balance between a positive effect associated with reduced antiviral silencing defenses and a negative effect caused by the decreased accumulation of specific suppressor variants. However, this does not exclude the possibility that in transgenic plants, the lack of interference between variants infecting the plant may be affected. We have now included this paragraph in the Discussion.

1-Insufficient functional characterization of novel mutations, especially 329Gly→Glu.
The negative result: Fig 8 shows that the 329Gly→Glu mutation (chimera 5) does not alter local titers, silencing suppression activity, or transient accumulation compared to chimera 3. This is an important negative finding, but the authors simply state they “could not show any effect.” The mutation became prevalent in one plant (WT/RT plant 2, Fig 7).

The authors should **discuss** whether the mutation might affect other HCPro functions (e.g., DCL interaction, subcellular localization, aphid transmission) that were not tested.

If no tested function is altered, propose alternative explanations: e.g., the mutation confers fitness advantage only under specific within-host conditions not recapitulated in standard assays, or it acts at the RNA level (secondary structure, replication efficiency) rather than protein function. The current discussion avoids this tension.

We have now mentioned in the Discussion section that although our assessment of mutation 329 found it neutral with regard to silencing agropatch suppression, local viral titers, and systemic prevalence, other functions might be affected by the mutation that provide a competitive advantage.

2-Missing methodological details.

Fitness difference calculation (Table 1): The formula used is ln(OR_Bt5/OR_At5) - ln(OR_Bt0/OR_At0). The authors state initial ratios (t0) are 0.5/0.5 because equal OD600 were used. However, Fig 5C shows that individual cells can have very different ratios, likely due to uneven T-DNA copy number per cell. Was the 0.5/0.5 ratio independently validated (e.g., by co-infiltration of two fluorescent markers)? If not, acknowledge this as a limitation in the Discussion.

Co-infiltrations were performed at OD 600 of 0.3, and we were really very careful about that requisite in all our experimental procedures, as it was a basic factor for the experiments performed in the manuscript.

With regard to the visual variability of GFP- and mRFP-derived fluorescence among epidermal cells, while GFP remains mostly soluble, mRFP tends to accumulate in numerous cytoplasmic inclusions of different number and sizes, depending on the cell (Figure 5D). We mentioned this fact in our initial submission but we failed to realize that this variability prevents the use of visualized fluorescence in co-infected epidermal cells as a means to assess variant co-infection levels in individual cells. This is because excitation by confocal lasers of the fluorophore in soluble proteins, and consequent emission of light would be more efficient for soluble proteins than for proteins clustered in inclusions. Therefore, the visual assessment of relative protein content in individual cells may be biased by the different number of mRFP inclusions that each cell has.

On the other hand, the western blot data in **Fig** **5C** assess GFP and mRFP levels in total proteins extracted from the infiltrated leaf in single infections by chimera 3 vs. co-infections by chimeras 3 + 2R. And likewise, of mRFP in single infections by chimera 2R vs. co-infection by chimeras 3 + 2R. In co-infections, both GFP and mRFP levels decreased relative to single infections, the decrease was less marked in the case of GFP (chimera 3), and more so in the case of mRFP (chimera 2R). Since chimeras 2 and 2R accumulate equally (**Fig** **5B**), this is consistent with what would be expected from the expected genomic analysis of local co-infection ratios of chimeras 2 and 3 (Figure 4A, upper and middle pie charts). We have replaced the charts shown in Figure 5C with new ones, after adding data from an additional replica experiment.

Considering these two facts combined, we limit our visual confocal conclusions in the Discussion section to saying that we detect co-infection in most epidermal cells. But we also say that serological assessment of the two reporters in total proteins extracted from the patch (Figure 5C) supports that chimeras 2R+3 co-infection ratios are in line with those obtained for chimeras 2+3 (Figure 4A). Note that chimeras 2R and 2 accumulate the same in single infections (Figure 5B).

2-Statistical reporting: The manuscript uses t-tests (e.g., P<0.05) but does not specify in Methods or Figure legends: number of biological replicates, n per replicate, whether t-tests were paired/unpaired, one‑/two‑tailed, and whether multiple comparison correction was applied. For example, Fig 2 and 3 legends only state “combined data from at least two independents.” Please provide exact replication numbers and statistical details.

The number of biological replicates in local assays (agropatch discs, n) for genomic data Figures is indicated in the corresponding Figure legend, while for protein Figures (western blot and derived charts), n is indicated and in the last page of the S1_raw_images file. Overall, our experiments used 4 to 12 independent biological replicates/case in the case of protein assessment by western blot, and 5 to 11 in the case of Sanger sequencing.

With regard to variant proportions, we have replaced the t-test analyses with more adequate tests, which are now detailed in the Methods section:

-For statistical assessment of co-infecting variant proportions within infiltrated discs (Figures 3 and 4), we used a **two-tailed Wilcoxon signed-rank test** for paired samples. The null hypothesis assumed equal representation of variant A and variant B within co-infected discs, corresponding to a theoretical median pA of 0.5. These proportions were estimated from the relative peak areas of Sanger sequencing chromatograms obtained from independent biological samples.

-For pairwise comparisons involving two independent groups (i.e., chimera 1 in treatment 1+2 versus chimera 1 in treatment 1+3 in (Figure 3), differences were assessed using **two-tailed unpaired the Mann–Whitney U test.**

-Statistical assessment of differences in the proportion of either chimera 3 or chimera 2 in local co-infections under three experimental groups (WT/RT, D2D4/RT, and WT/HT; Figure 4A) was performed using a **one-way analysis of variance ANOVA for independent groups, followed by two-tailed Games–Howell post hoc tests** to account for unequal variances among groups.

**Reviewer #3:**

1- the use of clonal chimeras that differ by one or two nucleotides in HCPro is valuable, as it reduces background genetic noise. Also, the comparation of mutations associated with enhanced silencing suppression and enhanced proteolytic cleavage is biologically meaningful. However, despite these aspects, viral accumulation was evaluated at local agroinfiltrated tissue at 5 dpi and systemic tissues at 29 dpi, assuming that equal Agrobacterium OD produces equal T-DNA delivery and comparable initial expression. From my point of view, this artificial agroinfiltration is not equivalent to equal cellular infection. While this could be appropriate as an experimental starting point, there are studies that emphasize that **the order of viral infection can strongly influence mixed infection outcomes**. In fact, the Fig 5 and results from visualization of local co-infections state a clear cell-to-cell variation in reporter ratios. I think these key aspects must be examined, clarified, and perhaps discussed as potential limitations. Furthermore, the authors mention the “absence of movement” and it should be clarified whether refers to systemic movement, minimal **cell-to-cell** spread relative to saturated infiltration, or a mechanistic block of movement.

We agree with the reviewer on the influence of different infiltration densities, or their order in infiltration, on infection outcomes. This is why we use strictly equal infiltration conditions throughout all our experiments. These facts are expressly mentioned in the main text.

For clarity, we have replaced in the manuscript the expression “absence of movement” with absence of cell-to-cell movement in the agroinfiltrated patches.

With regard to the visual variability GFP- and mRFP-derived fluorescence among epidermal cells, we failed to notice that the variability among cells in the number of mRFP inclusions prevents the use of visualized fluorescence in co-infected epidermal cells by confocal microscopy as a means to assess variant co-infection levels in individual cells. This is because excitation by confocal lasers of the fluorophore in soluble proteins, and consequent emission of light would be more efficient for soluble proteins than for proteins clustered in inclusions. However, as mentioned to reviewer 2, assessment of reporter levels by western blot, relative to single infections (Figure 5C) supports supports chimeras 2R/3 ratios that are consistent with ratios obtained from genomic data in Figure 4A for chimeras 2/3 (please note that chimera 2 and 2R accumulate the same in single infections, as shown in Figure 5B). In this regard, we have replaced the charts shown in Figure 5C with new ones, after adding data from an additional replica experiment.

Second, I think that the inclusion of elevated temperature and Dcl2/Dcl4-compromised plants is relevant, as temperature can alter host-virus interactions, RNA silencing, and protein stability,... However, my impression is that D2D4 plants are more than a “biotic parameter”: The Dcl2/Dcl4-silenced line is a host-genotype, including host-defense perturbation, **not simply a biotic condition**. Having broad effects on antiviral defense and plant physiology, the authors should further explain the rationale behind, and potential implications.

We agree with the reviewer that DCL-silenced transgenic plants are a host-genotype of *N. benthamiana*, but they also constitute also a different biotic environment for the infecting virus. This is an interesting issue, because we must not forget that wild-type (WT) *N. benthamiana,* though natural, is itself an unusual natural host: an *RDR1* mutation impairs its antiviral defenses so much that most plant viruses can infect it, infection titers are usually high, and also transient expression levels from agroinfiltrated T-DNAs are high. Additionally, its large epidermal cells are excellent for in vivo cell biology studies. Because of these properties it is a choice experimental plant, despite it being open to question how representative of natural hosts or crops this plant actually is.

DCL-silenced transgenic plants can be seen as further impaired in their antiviral defenses than the WT plant because of their low synthesis of 21-22 nt sRNAs. However, it is known that this further impairment greatly enhances the virulence of poorly adapted viruses (such as our early chimeras; Sun et al., 2024), but much less so, those of adapted viruses (Katsarou et al., 2018, and our observations), for reasons not well understood.

Another aspect is that replication assays is unclear. Figure 2 states that charts combine data from “at least two independents”, but I cannot find the number of biological replicates, technical replicates, leaves, plants, or independent experimental days. This is a major limitation because the statistical tests depend critically on the true unit of replication. Actually, the statistical methods are not sufficiently described in the Methods.

The **unit of replication** (n) in all our local assays is the infiltrated 21 mm diameter agropatch leaf disc. These discs were processed for total protein and total RNA extraction for analysis. We infiltrated fully expanded leaves of non-flowering plants, either two or three leaves. Neither young upper leaves nor older lower leaves. Infiltrated discs were extracted with a borer. When possible, we infiltrate each side of the same leaf with things we want to compare (for example, chimera 2 vs. chimera 3). The value of n is now indicated in every Figure legend for genetic assessments, and as a table at the end of the S1 raw file, for western blot assessments.

At least two separate experiments were performed in this way for all experiments of the manuscript, except in the cases of Sanger variant ratios of chimeras 1+2 and 1+3 co-infections at WT/RT shown in Figure 3A; in any case, in each experiment there were 5 replicates (however, protein assessment in these two same cases (shown in Figure 2) were the result of two separate experiments (S1 raw file, Figures A and B).

Experimental days for local assays lasted 5 days from infiltration to collection of discs for protein and total RNA extraction. Experimental days from infiltration for systemic assays were 29 days in the cases of chimeras 2 + 3 co-infection, and 3 + 4 co-infection. And only 14 days in the case of chimeras 3 + 5, until sample collection date. These details are now indicated in the Methods Section.

As mentioned to reviewer 2, some statistical tests have been altered, which are now described in the Methods section, and indicated also in the corresponding Figure legends.

The nanopore analysis needs more details. For example, the threshold was set because the control produced 1.6% and 0.67% recombinant-like reads, but S1 Table also reports several low-frequency mutations in the 3-5% range in the control and experimental pools. I would recommend showing per-sample read depth, or raising the threshold unless variants are independently validated by Sanger. Also I would like to see indel treatment, and the logic used to classify reads as chimera 1, 2, 3, 4, or novel mutants.

For the Nanopore analyses, viral reads were identified by aligning all sequencing reads against the WT HCPro reference sequence. Only reads showing confident viral alignments were retained for downstream analyses. In addition, only sequences with a mean quality score (Q score) ≥ 20 were included in downstream analyses.

To further minimize false-positive variant calls arising from Nanopore sequencing errors, only mutations within the HCPro cistron meeting both of the following criteria were considered: (i) a variant frequency ≥3% and (ii) a sequencing depth ≥ 79 reads (Table S1).

Furthermore, all newly detected mutations were independently verified at the single-read level using IGV (Integrative Genomics Viewer) software, enabling read-by-read validation of sequence variation. Because these additional mutations were detected at low frequencies, they are reported only descriptively.

The coverage depths are now indicated in S1 Table and in Figure 7.

Regarding the indel treatment, the reads containing indels were retained only when the overall alignment remained consistent with the reference sequence and did not interfere with the identification of diagnostic mutations used for chimera classification.

For chimera detection, all viral reads were systematically analyzed based on the presence of different mutation combinations at the aa 352 and 454, which were used to define and classify the different chimeric virus types.

**Part III – Minor Issues: Editorial and Data Presentation Modifications**
Please use this section for editorial suggestions as well as relatively minor modifications of existing data that would enhance clarity.

**Reviewer #1: (**No Response)

**Reviewer #2:**

Minor concerns on Fig 2B y‑axis scale: Total titers in WT/HT conditions are extremely low (~0.04) compared to other conditions (0.6–1.0). Please confirm that Western blot detection remains linear at such low levels (close to the detection limit). If not validated, state this as a limitation in the figure legend or Methods.

We mention in the Discussion section this potential limitation on the linearity of detection at such low titers.

Fig 5C fluorescence images: The overlay shows a wide variation in G/R ratio across cells. Please add a quantitative analysis (e.g., measure G/R in 20–30 cells) to support the interpretation that T-DNA copy number varies per cell, rather than relying on visual impression.

As mentioned already, we think that visual confocal assessment of reporter (GFP; mRFP) levels among individual epidermal cells shown in Figure 5D is not possible because differences among cells in the number of inclusions that the mRFP reporter creates could alter the amount of emitted fluorescence when those cells are excited by the lasers. Thus, it makes no sense to use the data to extrapolate relative variant ratios.

However, the western blot data in Figure 5C provides an overall quantitative assessment for reporter levels in the whole leaf patch: while in co-infections both GFP and mRFP levels decreased relative to single infections, the decrease was less marked in the case of GFP (chimera 3), and more in the case of mRFP (chimera 2R). Since chimeras 2 and 2R accumulate equally (Figure 5B), these results from protein reporters correlate well and validate expected ratios from genomic data (Figure 4A).

We mention now these two facts in the Discussion section, and limit our visual confocal claim to saying that in most cells, co-infection occurs. But we also say that in the patch, co-infecting viral ratios are consistent with those that would be expected from the genomic data.

Model of “early fixed ratio” in Discussion: The authors propose that viral translation products quickly sequester limited resources, fixing the ratio of replication complexes early, and that this ratio does not change over time. This model would benefit from direct evidence (e.g., time‑course sampling at 1, 2, 3, 4, 5 dpi to track ratio changes). If no such data exist, clearly state that this is a hypothesis, not a conclusion.

We have now stated that this is a hypothesis

Reference numbering: In‑text citations and the reference list appear inconsistent (e.g., the formula from Maree et al. 2000 is cited but not clearly linked in text). Please check throughout.

The reference Maree et al. 2000 is cited in Results section [in equation (1) and in Table 1]. Using equation (1) we found that chimera 3 had higher fitness than chimera 2 under the three environments assessed, in particular under elevated temperature

**Reviewer #3:**

Some other minor issues and suggestions:
I also think the authors should better separate three conceptual levels: trans-complementation within cells, competition among replication complexes, and systemic selection during movement and colonization. These processes can produce similar final ratios but have different mechanisms.

We mention in the Discussion section that we find no evidence for trans-complementation. Indeed, in local tissues, we hypothesize that co-infecting variants replicate independently, based on the fact that their expected co-infection ratios can be deduced from their titers in single infections, under our experimental conditions of equal agroinfiltration ODs.

I also find valuable that viral levels in WT/HT conditions are affected, but wondering if the very low titers at 29 °C mean that ratio estimates remain robust under low accumulation.

We mention this potential limitation in the Discussion section.

Figure 2: The bar plots should state the y-axis label, sample size, error bar meaning, and exact replicate number.

Those are now indicated in the Figure and its legend.

Figures 3 and 4 show mean ratios but not replicate dispersion. I find difficult to assess whether the statistical conclusions are driven by consistent biological replicates. Also, I think the use of Student t-tests for ratio comparisons is not appropriated, and should look for binomial or mixed-model approaches. In fact, this study compares multiple chimera combinations and conditions, and the rationale for each test is unclear.

We have now included the standard deviations for each pie chart shown in Figures 3 and 4.

The number of biological replicates in local assays (agropatch discs, n) in genomic data Figures is indicated in the corresponding Figure legend, while for protein Figures (western blot and derived charts), n is indicated and in the last page of the S1_raw_images file. Overall, our experiments used 4 to 12 independent biological replicates/case in the case of protein assessment by western blot, and 5 to 11 in the case of Sanger sequencing.

Use of Student t-tests is indeed not the most adequate means of assessing our variant proportions. As mentioned also to reviewer 2, we have replaced this test with the following tests:

-For statistical assessment of co-infecting variant proportions within infiltrated discs (Figures 3 and 4), we used a **two-tailed Wilcoxon signed-rank test** for paired samples. The null hypothesis assumed equal representation of variant A and variant B within co-infected discs, corresponding to a theoretical median pA of 0.5. These proportions were estimated from the relative peak areas of Sanger sequencing chromatograms obtained from independent biological samples.

-For pairwise comparisons involving two independent groups (i.e., chimera 1 in treatment 1+2 versus Chimera 1 in treatment 1+3 in (Figure 3), differences were assessed using **two-tailed unpaired the Mann–Whitney U test.**

-Statistical assessment of differences in the proportion of either Chimera 3 or chimera 2 in local co-infections under three experimental groups (WT/RT, D2D4/RT, and WT/HT; Figure 4A) was performed using a **one-way analysis of variance ANOVA for independent groups, followed by two-tailed Games–Howell post hoc tests** to account for unequal variances among groups.

Figure 5: The authors should either quantify cell-level GFP/mRFP ratio distributions or clearly state that the microscopy supports co-presence and heterogeneity but not precise variant abundance.

We agree with the reviewer. With regard to the visual variability in fluorescence among epidermal cells (Figure 5D), as mentioned to reviewer 2, we had failed to grasp the implication that mRFP accumulates more inclusions in some cells than in others, certainly more than does the GFP, which is mostly soluble. This variability prevents determining the relative presence of the two reporters in a given cell, because excitation by confocal lasers of the fluorophore in soluble proteins and detection of its emission would be far more efficient if proteins are soluble than if they clustered in inclusions. Therefore, we have rewritten comments in the Discussion to take this into account.

However, on the other hand, the western blot analyses of total proteins from whole agropatch discs (which are mostly mesophyll cells; Figure 5C) provides quantitative data that support that co-infection ratios are consistent with those expected from genomic data: relative levels of GFP in single infections by chimera 3 vs. co-infections by chimeras 3 + 2R. Or of mRFP in single infections by chimera 2R vs. co-infection by chimeras 3 + 2R indicate that in co-infections they both decrease, but less in the case of GFP (chimera 3) than in the case of mRFP (chimera 2R). These data from virally-expressed protein reporters correlate and validate the data on genomic ratios (for chimera 2+3, shown in Figure 4A, upper two rows of pie charts).

L26: Use “environmental factors.”

Done

L86: “regardless of the mechanism.”

Done

L142-154: I would recommend stating that the gap is not simply that mixed infections matter.

It is not clear to us what the reviewer meant, and we left the paragraph as it is

L234-235: Use “P < 0.05, Student’s t-test.” Also consider whether this test is appropriate for proportional data.

As mentioned above, we agree that this test is not the most appropriate for proportional data assessment, and we have replaced it with other tests, depending on the characteristics of the experiments, also indicated above.

L242: Data ... are shown.”

Done

L267-270: revise parentheses of the equation.

Corrected

L301-305: are available WT/RT nanopore data? Why?

We did not obtain meaningful reads from that sample. We think that the ds cDNA synthesis failed. Secugen requires a minimum of 10 samples to nanopore sequence, and the cost of repeating it was thus elevated. We do not think nanopore data from this sample is critical.

L329: Consider “could be a candidate temperature-associated mutation requiring functional validation.”

Done

L339: should be Fig. 7?

Done

L390-392: should be Fig 2, 3, and 4A.?

Done

465-467: Was DCL2 and DCL4 knockdown efficiency confirmed in the plants used?

No, but we use these plants routinely in the lab for a number of years, and they are consistent between individual plants. We have not seen differences in their transient expression levels of a GFP reporter under UV lamp, for example.

540: Fig 9?

It was Fiure 8. Corrected

**Journal Requirements:**

1) Please ensure that the CRediT author contributions listed for every co-author are completed accurately and in full

At this stage, the following Authors/Authors **require contributions**: María Fernández-Avilés, Francisco Tenllado, and Tomas Canto. Please ensure that the full contributions of each author are acknowledged in the "Add/Edit/Remove Authors" section of our submission form.
The list of CRediT author contributions may be found here: <https://journals.plos.org/plospathogens/s/authorship#loc-author-contributions>

We have completed the CRediT contributions for all authors.

2) We noticed that you used the phrase 'data not shown' in the manuscript. We do not allow these references, as the PLOS data access policy requires that all data be either published with the manuscript or made available in a publicly accessible database. Please amend the supplementary material to include the referenced data or remove the references.

The expression “data not shown” and its referenced data have been removed.

3) We do not publish any copyright or trademark symbols that usually accompany proprietary names, eg ©,  ®, or TM  (e.g. next to drug or reagent names). Therefore, please remove all instances of trademark/copyright symbols throughout the text, including:
- ® on pages: 16, and 51

- TM on page: 28.

All copyright and trademark symbols have been removed from the manuscript text.

4) Please upload all main figures as separate Figure files in .tif or .eps format. For more information about how to convert and format your figure files please see our guidelines: 
<https://journals.plos.org/plospathogens/s/figures>

All Figures have been uploaded separately from the manuscript main text.

5) Please upload a copy of Figure Fig 1-8 which you refer to in your text on pages 14, 15, 17, 18, 19, and 20. Or, if the figure is no longer to be included as part of the submission please remove all reference to it within the text.

Done.

6) We notice that your supplementary Figures, and Tables are included in the manuscript file. Please remove them and upload them with the file type 'Supporting Information'. Please ensure that each Supporting Information file has a legend listed in the manuscript after the references list.

Supplementary Figures and Tables have been uploaded separately as “Supporting Information”

7) We have amended your Competing Interest statement to comply with journal style. We kindly ask that you double check the statement and let us know if anything is incorrect.

The amended competing interest statement is in order, thank you.

8) Your current Financial Disclosure states, “This work was funded by a grant awarded to TC and FT number PID2022-137691OB-I00, from the Spanish Ministry of Science and Innovation (MCIN/AEI/10.13039/501100011033), and by the European Union Next Generation EU/PRTR. https://www.ciencia.gob.es/. The funder did not play any role in the study design, data collection and analysis, decision to publish, or preparation of the manuscript.”

However, the information provided in the Financial Disclosure statement does not match the funding information submitted in the submission form. Please indicate by return email the full and correct funding information for your study and confirm the order in which funding contributions should appear. Please be sure to indicate whether the funders played any role in the study design, data collection and analysis, decision to publish, or preparation of the manuscript.

We have e-mailed the correct funding information.

9) PLOS now requires that authors provide **the original uncropped and unadjusted images** underlying all blot or gel results reported in a submission’s figures or Supporting Information files. This policy and the journal’s other requirements for blot/gel reporting and figure preparation are described in detail at <https://journals>.plos.org/plosone/s/figures#loc-blot-and-gel-reporting-requirements and <https://journals>.plos.org/plosone/s/figures#loc-preparing-figures-from-image-files.

When you submit your revised manuscript, please ensure that your figures adhere fully to these guidelines and provide the original underlying images for all blot or gel data reported in your submission. See the following link for instructions on providing the original image data: <https://journals>.plos.org/plosone/s/figures#loc-original-images-for-blots-and-gels.

We have also uploaded as Supporting Information all the raw blots that were used in the western blot Figures shown in the manuscript, and also any additional western blots that were used for the making of charts in the manuscript Figures (through densitometry analysis), in an S1_raw_images pdf file. For each set of raw blots, we have included an assembled figure (whether it is the Figure shown in the manuscript or not) that allows the reader to identify in the raw blots each lane and protein band.

In your cover letter, please note whether your blot/gel image data are in Supporting Information or posted at a public data repository, provide the **repository URL if relevant**, and provide specific details as to which raw blot/gel images, if any, are not available. Email us at customercare@plos.org if you have any questions.

It is mentioned in the cover letter that an S1_raw_images file has been uploaded as Supporting Information
