# Supplementary material for: Competition among co-infecting potyviral chimeras with adaptive, single-nucleotide differences in HCPro: Properties, and influence of environment factors on outcomes
Source: PLoS Pathog. 2026 Aug 18;22(8):e1014529. doi: 10.1371/journal.ppat.1014529 (PMC13502915; doi:10.1371/journal.ppat.1014529)
Supplement: Supplementary file 5 [file ppat.1014529.s008.docx]

**Part I – Summary**

Reviewer #1: This manuscript investigates the competitive dynamics of potyviral chimeras carrying different combination of adaptive mutations in the HCPro, under both local and systemic co-infection conditions. The authors systematically examine whether co-infecting variants influence each other's accumulation, assess the impact of abiotic (higher temperature) and biotic (DCL2/4-silenced host) factors, and track further adaptive evolution via novel mutations. The work addresses fundamental questions in RNA virus quasi-species evolution, within-host competition, and environmental modulation of viral fitness, using well-controlled experimental assays. The results of this study will help us better understand RNA virus evolution

We thank the reviewer for the positive assessment of the manuscript

Reviewer #2: The revised manuscript is recommended for publication.

We thank the reviewer for the positive assessment of the manuscript

Reviewer #3: The authors have adequately addressed most of my comments from the previous version. There is a notable improvement in the clarity and overall quality of the work. The wording has been moderated and the conclusions tempered, mainly with the overinterpretation of the confocal images. However, there are still two issues that remains without satisfying reply.

We thank the reviewer for the positive assessment of the manuscript. We have addressed the reviewer´s two remaining issues (see below)

**Part II – Major Issues: Key Experiments Required for Acceptance**

Reviewer #1: No comments.

Reviewer #2: (No Response)

Reviewer #3: The concern is about the key experimental assumption of equal OD600. To me, having an equal OD600 does not establish equal cellular co-entry, and should be validate if that perform equal T-DNA copy number, or equal timing of intracellular expression. Somehow, it needs a direct control to test this assumption. I reckon this validation can be biologically complicated, but **this limitation should be** **explicitly discussed in the manuscript**, and the relevant claims should be rephrased. For example, "**under our tested local agroinfiltration conditions….**".

Our studies rest on the assumption that overall, equal agroinfiltration ODs cause equal amounts of T-DNAs to enter and become expressed in the cells of agroinfiltrated leaf discs. Based on our data (nucleic acid and protein), we are confident that this is a sound premise, but we agree with the reviewer that this assumption must be clearly indicated in the manuscript. We have therefore altered the main text accordingly.

**Part III – Minor Issues: Editorial and Data Presentation Modifications**
Please use this section for editorial suggestions as well as relatively minor modifications of existing data that would enhance clarity.

Reviewer #1: The statement regarding cross-protection in **lines 76–78** is not entirely accurate. The authors write: "This is because infection by one virus excludes infection by a very similar virus." However, this characterization oversimplifies the phenomenon. In cross-protection, the **attenuated (protecting) virus must establish a systemic infection first;** only after a sufficient time interval does the plant develop resistance that prevents superinfection by a second, closely related virulent strain. If both viruses are inoculated simultaneously or too close in time, co-infection can occur rather than exclusion. The authors should revise this sentence to reflect the temporal requirement for cross-protection and clarify that exclusion is not an immediate or automatic consequence of co-inoculation.

We thank the reviewer for this important clarification. Our original statement oversimplified the concept of cross-protection. We have revised the statement in lines 76-78 to emphasize cross-protection requirements.

The authors conclude that no evidence of synergism was observed in co-infections (lines 174–175, Fig. 2). In several cases, the observed titers in mixed infections are lower than those in single infections of the more fit variant (e.g., chimera 3 alone accumulates higher than that of chimera 3+4 co-infection; chimera 3 alone is higher than that of chimera 2+3 co-infection). This reduction, while not amounting to strong antagonism, may indicate a mild competitive or resource-limiting effect when two variants share the same cellular environment. This also supported by the confocal microscopy analysis, most of the fluorescences is not overlayed. The authors should consider discussing whether these slight decreases in titers reflect competition for limited host resources, replication sites, or other cellular factors, rather than simply stating "no evidence of synergism." A more nuanced interpretation—acknowledging a trend toward mild inhibition in some combinations—would strengthen the discussion.

We agree that viral accumulation in mixed infections is lower than that observed for the corresponding single infection of the dominant variant. We interpret that in mixed infections, total viral titers are shared between two independently replicating variants, each contributing to the overall viral accumulation according to its relative proportion. Consequently, the accumulation of the more fit variant in a mixed infection is expected to be lower than when it infects alone, because it represents a fraction of the total viral population.

Our data support the idea that replication complexes are established early after infection and subsequently replicate largely independently. We found no evidence of trans-complementation between variants, no detectable influence of one HCPro on the replication of the other, and only extremely low frequencies of homologous recombination, indicating very limited interactions between co-infecting variants. We have revised the Discussion to make sure this interpretation is present

Reviewer #2: (No Response)

Reviewer #3: The minor issue is the note that "**The manuscript should sharpen the knowledge gap rather than simply stating that mixed infections matter**". This aspect carries conceptual significance, whereas the novelty of this work lies specifically in the competition of nearly isogenic variants carrying distinct adaptive changes in a multifunctional protein, not in mixed infection per se. Within-host competition should be framed and extended.

We agree with the reviewer that the novelty of our study lies in the study of how nearly isogenic viral variants carrying single, adaptive mutations in the multifunctional protein HCPro, co-exist locally, compete systemically, and adapt further. Rather than studying cases of mixed infections. We have revised the Introduction to better mention this specificity and novelty.

**Journal Requirements:**

1)Thank you for stating "The sequence of the HCPro cistron of chimera 3 (single- or double-nucleotide differences of the HCPro cistrons in the other chimeras 1, 2, 2R, 4, and 5 used in this work can be found in Fig 1), as well as Sanger sequences used in this work are available through the following link: https://saco.csic.es/s/F2JgNi5XXqdoGRN." However, we were unable to access the data using the information provided. Please review and update the Data Availability Statement to ensure that the underlying data are publicly accessible and that all accession numbers, links, or repository details are correct and functional.

We have checked the web address, and it works. The problem appears to be the dot at the end of the line, which is not part of the address. We have deleted the dot from the submission
