# Supplementary material for: Competition among co-infecting potyviral chimeras with adaptive, single-nucleotide differences in HCPro: Properties, and influence of environment factors on outcomes
Source: PLoS Pathog. 2026 Aug 18;22(8):e1014529. doi: 10.1371/journal.ppat.1014529 (PMC13502915; doi:10.1371/journal.ppat.1014529)
Supplement: Peer review history

# Peer review: Decision Letter 0

**DOI:** 10.1371/journal.ppat.1014529.r001

**Authors:** Shou-Wei Ding, Xian-Bing Wang

16 Jun 2026

Competition among co-infecting potyviral chimeras with adaptive, single-nucleotide differences in HCPro: properties, and influence of environment factors on outcomes

PLOS Pathogens

Dear Dr. Canto,

Thank you for submitting your manuscript to PLOS Pathogens. After careful consideration, we feel that it has merit but does not fully meet PLOS Pathogens's publication criteria as it currently stands. Therefore, we invite you to submit a revised version of the manuscript that addresses the points raised during the review process.

Please submit your revised manuscript by Aug 15 2026 11:59PM. If you will need more time than this to complete your revisions, please reply to this message or contact the journal office at plospathogens@plos.org. When you're ready to submit your revision, log on to https://www.editorialmanager.com/ppathogens/ and select the 'Submissions Needing Revision' folder to locate your manuscript file.

Please include the following items when submitting your revised manuscript:

* A letter that responds to each point raised by the editor and reviewer(s). You should upload this letter as a separate file labeled 'Response to Reviewers'. This file does not need to include responses to any formatting updates and technical items listed in the 'Journal Requirements' section below.

* A marked-up copy of your manuscript that highlights changes made to the original version. You should upload this as a separate file labeled 'Revised Manuscript with Track Changes'.

* An unmarked version of your revised paper without tracked changes. You should upload this as a separate file labeled 'Manuscript'.

We look forward to receiving your revised manuscript.

Kind regards,

Xian-Bing Wang

Academic Editor

PLOS Pathogens

Shou-Wei Ding

Section Editor

Editor-in-Chief

PLOS Pathogens

orcid.org/0000-0003-2946-9497

Editor-in-Chief

PLOS Pathogens

orcid.org/0000-0002-7699-2064

**Additional Editor Comments (if provided):**

**Journal Requirements:**

If the reviewer comments include a recommendation to cite specific previously published works, please review and evaluate these publications to determine whether they are relevant and should be cited. There is no requirement to cite these works unless the editor has indicated otherwise.

1) Please ensure that the CRediT author contributions listed for every co-author are completed accurately and in full.

At this stage, the following Authors/Authors require contributions: María Fernández-Avilés, Francisco Tenllado, and Tomas Canto. Please ensure that the full contributions of each author are acknowledged in the "Add/Edit/Remove Authors" section of our submission form.

The list of CRediT author contributions may be found here: https://journals.plos.org/plospathogens/s/authorship#loc-author-contributions

2) We noticed that you used the phrase 'data not shown' in the manuscript. We do not allow these references, as the PLOS data access policy requires that all data be either published with the manuscript or made available in a publicly accessible database. Please amend the supplementary material to include the referenced data or remove the references.

3) We do not publish any copyright or trademark symbols that usually accompany proprietary names, eg ©,  ®, or TM  (e.g. next to drug or reagent names). Therefore please remove all instances of trademark/copyright symbols throughout the text, including:

- ® on pages: 16, and 51

- TM on page: 28.

4) Please upload all main figures as separate Figure files in .tif or .eps format. For more information about how to convert and format your figure files please see our guidelines:

https://journals.plos.org/plospathogens/s/figures

5) Please upload a copy of Figure Fig 1-8 which you refer to in your text on pages 14, 15, 17, 18, 19, and 20. Or, if the figure is no longer to be included as part of the submission please remove all reference to it within the text.

6) We notice that your supplementary Figures, and Tables are included in the manuscript file. Please remove them and upload them with the file type 'Supporting Information'. Please ensure that each Supporting Information file has a legend listed in the manuscript after the references list.

7) We have amended your Competing Interest statement to comply with journal style. We kindly ask that you double check the statement and let us know if anything is incorrect.

8) Your current Financial Disclosure states, “This work was funded by a grant awarded to TC and FT number PID2022-137691OB-I00, from the Spanish Ministry of Science and Innovation (MCIN/AEI/10.13039/501100011033), and by the European Union Next Generation EU/PRTR. https://www.ciencia.gob.es/ .The funder did not play any role in the study design, data collection and analysis, decision to publish, or preparation of the manuscript.” However, the information provided in the Financial Disclosure statement does not match the funding information submitted in the submission form. Please indicate by return email the full and correct funding information for your study and confirm the order in which funding contributions should appear. Please be sure to indicate whether the funders played any role in the study design, data collection and analysis, decision to publish, or preparation of the manuscript.

9) PLOS now requires that authors provide the original uncropped and unadjusted images underlying all blot or gel results reported in a submission’s figures or Supporting Information files. This policy and the journal’s other requirements for blot/gel reporting and figure preparation are described in detail at https://journals.plos.org/plosone/s/figures#loc-blot-and-gel-reporting-requirements and https://journals.plos.org/plosone/s/figures#loc-preparing-figures-from-image-files.

When you submit your revised manuscript, please ensure that your figures adhere fully to these guidelines and provide the original underlying images for all blot or gel data reported in your submission. See the following link for instructions on providing the original image data: https://journals.plos.org/plosone/s/figures#loc-original-images-for-blots-and-gels.

In your cover letter, please note whether your blot/gel image data are in Supporting Information or posted at a public data repository, provide the repository URL if relevant, and provide specific details as to which raw blot/gel images, if any, are not available. Email us at customercare@plos.org if you have any questions.

**Reviewers' Comments:**

Reviewer's Responses to Questions

**Part I - Summary**

Please use this section to discuss strengths/weaknesses of study, novelty/significance, general execution and scholarship.

Reviewer #1: This study analyzes the effects of single and co-infections of two natural variants, each harboring a single-amino acid mutation, on viral titers. It also provides insights into the possible biological significance of these mutations in response to biotic and/or abiotic stresses. While the authors tackle a complex subject and employ two different methods to assess mutant abundance, the evidence provided remains insufficient. Furthermore, the analysis is restricted exclusively to the HCPro sequence, leaving it unclear whether unexamined mutations exist elsewhere in the viral genome. Consequently, the current data do not fully substantiate the authors' conclusions.

Reviewer #2: This study systematically investigates the competition dynamics, environmental modulation, and further adaptive evolution of potyviral chimeras carrying single or double beneficial mutations in HCPro. The topic is theoretically significant, directly addressing classical questions about RNA virus quasispecies evolution, competitive exclusion, and co-infection dynamics. The work provides new experimental evidence for how plant RNA viruses fine-tune a multifunctional protein to adapt to environmental changes. However, several aspects require clarification, stronger evidential support, or reinterpretation.

Reviewer #3: This manuscript describes how isogenic potyviral chimeras, differing by one or two adaptive nucleotide substitutions in the HCPro, perform during local and systemic co-infection in Nicotiana benthamiana plants. I think that the rationale of this work is sound because HCPro is a multifunctional protein and whether beneficial mutations can act in trans on co-infecting genomes, or compete, and whether environmental conditions can alter the outcome is timely and very interesting. Moreover, it combines viral adaptation, within-host competition, environmental modulation, and the functional pleiotropy of HCPro.

**********

**Part II – Major Issues: Key Experiments Required for Acceptance**

Please use this section to detail the key new experiments or modifications of existing experiments that should be absolutely required to validate study conclusions.

Generally, there should be no more than 3 such required experiments or major modifications for a "Major Revision" recommendation. If more than 3 experiments are necessary to validate the study conclusions, then you are encouraged to recommend "Reject".

Reviewer #1: First, as the authors acknowledge, RNA viruses exhibit a high mutation rate. Although the sequences of the HCPro region were verified across different treatments, this targeted sequencing cannot rule out the possibility that the observed phenotypic differences are driven by spontaneous mutations elsewhere in the viral genome. Additional expeirments need to be carried out to verify this.

Second, using Western blot analysis to compare the accumulation levels of mutants tagged with either GFP or RFP is methodologically flawed. Because anti-GFP and anti-RFP antibodies inherently possess distinct binding affinities and detection sensitivities, the relative signal intensities of the protein bands cannot be directly correlated with the actual in planta accumulation levels of the respective viral mutants.

Reviewer #2: Major concerns

1. Potential circularity in the core conclusion that “variants do not influence each other.”

The authors use Equations 2 and 3 to argue that variants replicate independently: expected co-infection titers calculated from single-infection titers and observed co-infection ratios match the observed co-infection titers. This reasoning is partially circular because the “observed co-infection ratio” used in Equation 2 is itself derived from sequencing data, but the core test should rely on Equation 3 (expected ratio from single titers) compared to actual sequencing ratios. The discrepancy in D2D4/RT conditions (Table 3: expected ratio 0.50/0.50 vs. observed ~0.40/0.60) actually demonstrates that the environment can break the “independence” assumption – a critical finding.

The authors should:

Clearly distinguish between “expected ratio” (from single titers) and “observed ratio” (from sequencing).

Discuss why D2D4 conditions lead to deviation from the independent-replication model. Possibilities include altered relative accumulation rates of HCPro variants in a silencing-deficient background, as the authors briefly mention (lines 410–414).

2. Insufficient functional characterization of novel mutations, especially 329Gly→Glu.

The negative result: Fig 8 shows that the 329Gly→Glu mutation (chimera 5) does not alter local titers, silencing suppression activity, or transient accumulation compared to chimera 3. This is an important negative finding, but the authors simply state they “could not show any effect.” The mutation became prevalent in one plant (WT/RT plant 2, Fig 7).

The authors should:

Discuss whether the mutation might affect other HCPro functions (e.g., DCL interaction, subcellular localization, aphid transmission) that were not tested.

If no tested function is altered, propose alternative explanations: e.g., the mutation confers fitness advantage only under specific within-host conditions not recapitulated in standard assays, or it acts at the RNA level (secondary structure, replication efficiency) rather than protein function. The current discussion avoids this tension.

3. Missing methodological details.

Fitness difference calculation (Table 1): The formula used is ln(OR_Bt5/OR_At5) - ln(OR_Bt0/OR_At0). The authors state initial ratios (t0) are 0.5/0.5 because equal OD600 were used. However, Fig 5C shows that individual cells can have very different ratios, likely due to uneven T-DNA copy number per cell. Was the 0.5/0.5 ratio independently validated (e.g., by co-infiltration of two fluorescent markers)? If not, acknowledge this as a limitation in the Discussion.

Statistical reporting: The manuscript uses t-tests (e.g., P<0.05) but does not specify in Methods or Figure legends: number of biological replicates, n per replicate, whether t-tests were paired/unpaired, one‑/two‑tailed, and whether multiple comparison correction was applied. For example, Fig 2 and 3 legends only state “combined data from at least two independents.” Please provide exact replication numbers and statistical details.

Reviewer #3: However, there are some concerns and comments that should be addressed to gain clarity.

First, the use of clonal chimeras that differ by one or two nucleotides in HCPro is valuable, as it reduces background genetic noise. Also the comparation of mutations associated with enhanced silencing suppression and enhanced proteolytic cleavage is biologically meaningful. However, despite these aspects, viral accumulation was evaluated at local agroinfiltrated tissue at 5 dpi and systemic tissues at 29 dpi, assuming that equal Agrobacterium OD produces equal T-DNA delivery and comparable initial expression. From my point of view, this artificial agroinfiltration is not equivalent to equal cellular infection. While this could be appropriate as an experimental starting point, there are studies that emphasize that the order of viral infection can strongly influence mixed infection outcomes. In fact, the Fig 5 and results from visualization of local co-infections state a clear cell-to-cell variation in reporter ratios. I think these key aspects must be examined, clarified, and perhaps discussed as potential limitations. Furthermore, the authors mention the “absence of movement” and it should be clarified whether refers to systemic movement, minimal cell-to-cell spread relative to saturated infiltration, or a mechanistic block of movement.

Second, I think that the inclusion of elevated temperature and Dcl2/Dcl4-compromised plants is relevant, as temperature can alter host-virus interactions, RNA silencing, and protein stability,... However, my impression is that D2D4 plants are more than a “biotic parameter”: The Dcl2/Dcl4-silenced line is a host-genotype, including host-defense perturbation, not simply a biotic condition. Having broad effects on antiviral defense and plant physiology, the authors should further explain the rationale behind, and potential implications.

Another aspect is that replication assays is unclear. Figure 2 states that charts combine data from “at least two independents”, but I cannot find the number of biological replicates, technical replicates, leaves, plants, or independent experimental days. This is a major limitation because the statistical tests depend critically on the true unit of replication. Actually, the statistical methods are not sufficiently described in the Methods.

The nanopore analysis needs more details. For example, the threshold was set because the control produced 1.6% and 0.67% recombinant-like reads, but S1 Table also reports several low-frequency mutations in the 3-5% range in the control and experimental pools. I would recommend showing per-sample read depth, or raising the threshold unless variants are independently validated by Sanger. Also I would like to see indel treatment, and the logic used to classify reads as chimera 1, 2, 3, 4, or novel mutants.

**********

**Part III – Minor Issues: Editorial and Data Presentation Modifications**

Please use this section for editorial suggestions as well as relatively minor modifications of existing data that would enhance clarity.

Reviewer #1: (No Response)

Reviewer #2: Minor concerns

Fig 2B y‑axis scale: Total titers in WT/HT conditions are extremely low (~0.04) compared to other conditions (0.6–1.0). Please confirm that Western blot detection remains linear at such low levels (close to the detection limit). If not validated, state this as a limitation in the figure legend or Methods.

Fig 5C fluorescence images: The overlay shows a wide variation in G/R ratio across cells. Please add a quantitative analysis (e.g., measure G/R in 20–30 cells) to support the interpretation that T-DNA copy number varies per cell, rather than relying on visual impression.

Model of “early fixed ratio” in Discussion: The authors propose that viral translation products quickly sequester limited resources, fixing the ratio of replication complexes early, and that this ratio does not change over time. This model would benefit from direct evidence (e.g., time‑course sampling at 1, 2, 3, 4, 5 dpi to track ratio changes). If no such data exist, clearly state that this is a hypothesis, not a conclusion.

Reference numbering: In‑text citations and the reference list appear inconsistent (e.g., the formula from Maree et al. 2000 is cited but not clearly linked in text). Please check throughout.

Reviewer #3: Some other minor issues and suggestions:

I also think the authors should better separate three conceptual levels: trans-complementation within cells, competition among replication complexes, and systemic selection during movement and colonization. These processes can produce similar final ratios but have different mechanisms.

I also find valuable that viral levels in WT/HT conditions are affected, but wondering if the very low titers at 29 °C mean that ratio estimates remain robust under low accumulation.

Figure 2: The bar plots should state the y-axis label, sample size, error bar meaning, and exact replicate number.

Figures 3 and 4 show mean ratios but not replicate dispersion. I find difficult to assess whether the statistical conclusions are driven by consistent biological replicates. Also, I think the use of Student t-tests for ratio comparisons is not appropriated, and should look for binomial or mixed-model approaches. In fact, this study compares multiple chimera combinations and conditions, and the rationale for each test is unclear.

Figure 5: The authors should either quantify cell-level GFP/mRFP ratio distributions or clearly state that the microscopy supports co-presence and heterogeneity but not precise variant abundance.

L26: Use “environmental factors.”

L86: “regardless of the mechanism.”

L142-154: I would recommend stating that the gap is not simply that mixed infections matter.

L234-235: Use “P < 0.05, Student’s t-test.” Also consider whether this test is appropriate for proportional data.

L242: Data ... are shown.”

L267-270: revise parentheses of the equation.

L301-305: are available WT/RT nanopore data? Why?

L329: Consider “could be a candidate temperature-associated mutation requiring functional validation.”

L339: should be Fig. 7?

L390-392: should be Fig 2, 3, and 4A.?

465-467: Was DCL2 and DCL4 knockdown efficiency confirmed in the plants used?

540: Fig 9?

**********

PLOS authors have the option to publish the peer review history of their article (what does this mean?). If published, this will include your full peer review and any attached files.

If you choose “no”, your identity will remain anonymous but your review may still be made public.

**Do you want your identity to be public for this peer review?**  For information about this choice, including consent withdrawal, please see our Privacy Policy.

Reviewer #1: No

Reviewer #2: No

Reviewer #3: No

[NOTE: If reviewer comments were submitted as an attachment file, they will be attached to this email and accessible via the submission site. Please log into your account, locate the manuscript record, and check for the action link "View Attachments". If this link does not appear, there are no attachment files.]

**Figure resubmission:**

**Reproducibility:**

?>

# Author comment: Author response to Decision Letter 1

**DOI:** 10.1371/journal.ppat.1014529.r002

1 Jul 2026

Attachment Submitted filename: Response to Reviewers.docx

# Peer review: Decision Letter 1

**DOI:** 10.1371/journal.ppat.1014529.r003

**Authors:** Shou-Wei Ding, Xian-Bing Wang

24 Jul 2026

PPATHOGENS-D-26-01210R1

Competition among co-infecting potyviral chimeras with adaptive, single-nucleotide differences in HCPro: properties, and influence of environment factors on outcomes

PLOS Pathogens

Dear Dr. Canto,

Thank you for submitting your manuscript to PLOS Pathogens. After careful consideration, we feel that it has merit but does not fully meet PLOS Pathogens's publication criteria as it currently stands. Therefore, we invite you to submit a revised version of the manuscript that addresses the points raised during the review process.

Please submit your revised manuscript by Sep 22 2026 11:59PM. If you will need more time than this to complete your revisions, please reply to this message or contact the journal office at plospathogens@plos.org. When you're ready to submit your revision, log on to https://www.editorialmanager.com/ppathogens/ and select the 'Submissions Needing Revision' folder to locate your manuscript file.

Please include the following items when submitting your revised manuscript:

* A letter that responds to each point raised by the editor and reviewer(s). You should upload this letter as a separate file labeled 'Response to Reviewers'. This file does not need to include responses to any formatting updates and technical items listed in the 'Journal Requirements' section below.

* A marked-up copy of your manuscript that highlights changes made to the original version. You should upload this as a separate file labeled 'Revised Manuscript with Track Changes'.

* An unmarked version of your revised paper without tracked changes. You should upload this as a separate file labeled 'Manuscript'.

If you would like to make changes to your financial disclosure, competing interests statement, or data availability statement, please make these updates within the submission form at the time of resubmission. Guidelines for resubmitting your figure files are available below the reviewer comments at the end of this letter.

As the corresponding author, your ORCID iD is verified in the submission system and will appear in the published article. PLOS supports the use of ORCID, and we encourage all coauthors to register for an ORCID iD and use it as well. Please encourage your coauthors to verify their ORCID iD within the submission system before final acceptance, as unverified ORCID iDs will not appear in the published article. Only the individual author can complete the verification step; PLOS staff cannot verify ORCID iDs on behalf of authors.

We look forward to receiving your revised manuscript.

Kind regards,

Xian-Bing Wang

Academic Editor

PLOS Pathogens

Shou-Wei Ding

Section Editor

PLOS Pathogens

Sumita Bhaduri-McIntosh

Editor-in-Chief

PLOS Pathogens

orcid.org/0000-0003-2946-9497

Michael Malim

Editor-in-Chief

PLOS Pathogens

orcid.org/0000-0002-7699-2064

**Additional Editor Comments (if provided):**

**Journal Requirements:**

If the reviewer comments include a recommendation to cite specific previously published works, please review and evaluate these publications to determine whether they are relevant and should be cited. There is no requirement to cite these works unless the editor has indicated otherwise.

1)Thank you for stating "The sequence of the HCPro cistron of chimera 3 (single- or double-nucleotide differences of the HCPro cistrons in the other chimeras 1, 2, 2R, 4, and 5 used in this work can be found in Fig 1), as well as Sanger sequences used in this work are available through the following link: https://saco.csic.es/s/F2JgNi5XXqdoGRN." However, we were unable to access the data using the information provided. Please review and update the Data Availability Statement to ensure that the underlying data are publicly accessible and that all accession numbers, links, or repository details are correct and functional.

**Reviewers' Comments:**

Reviewer's Responses to Questions

**Part I - Summary**

Please use this section to discuss strengths/weaknesses of study, novelty/significance, general execution and scholarship.

Reviewer #1: This manuscript investigates the competitive dynamics of potyviral chimeras carrying different combination of adaptive mutations in the HCPro, under both local and systemic co-infection conditions. The authors systematically examine whether co-infecting variants influence each other's accumulation, assess the impact of abiotic (higher temperature) and biotic (DCL2/4-silenced host) factors, and track further adaptive evolution via novel mutations. The work addresses fundamental questions in RNA virus quasispecies evolution, within-host competition, and environmental modulation of viral fitness, using a well-controlled experimental assays. The results of this study will help us better understand RNA virus evolution.

Reviewer #2: The revised manuscript is recommended for publication.

Reviewer #3: The authors have adequately addressed most of my comments from the previous version. There is a notable improvement in the clarity and overall quality of the work. The wording has been moderated and the conclusions tempered, mainly with the overinterpretation of the confocal images. However, there are still two issues that remains without satisfying reply.

**********

**Part II – Major Issues: Key Experiments Required for Acceptance**

Please use this section to detail the key new experiments or modifications of existing experiments that should be absolutely required to validate study conclusions.

Generally, there should be no more than 3 such required experiments or major modifications for a "Major Revision" recommendation. If more than 3 experiments are necessary to validate the study conclusions, then you are encouraged to recommend "Reject".

Reviewer #1: No comments.

Reviewer #2: (No Response)

Reviewer #3: The concern is about the key experimental assumption of equal OD600. To me, having an equal OD600 does not establish equal cellular co-entry, and should be validate if that perform equal T-DNA copy number, or equal timing of intracellular expression. Somehow, it needs a direct control to test this assumption. I reckon this validation can be biologically complicated, but this limitation should be explicitly discussed in the manuscript, and the relevant claims should be rephrased. For example, "under our tested local agroinfiltration conditions….".

**********

**Part III – Minor Issues: Editorial and Data Presentation Modifications**

Please use this section for editorial suggestions as well as relatively minor modifications of existing data that would enhance clarity.

Reviewer #1: The statement regarding cross-protection in lines 76–78 is not entirely accurate. The authors write: "This is because infection by one virus excludes infection by a very similar virus." However, this characterization oversimplifies the phenomenon. In cross-protection, the attenuated (protecting) virus must establish a systemic infection first; only after a sufficient time interval does the plant develop resistance that prevents superinfection by a second, closely related virulent strain. If both viruses are inoculated simultaneously or too close in time, co-infection can occur rather than exclusion. The authors should revise this sentence to reflect the temporal requirement for cross-protection and clarify that exclusion is not an immediate or automatic consequence of co-inoculation.

The authors conclude that no evidence of synergism was observed in co-infections (lines 174–175, Fig. 2). In several cases, the observed titers in mixed infections are lower than those in single infections of the more fit variant (e.g., chimera 3 alone accumulates higher than that of chimera 3+4 co-infection; chimera 3 alone is higher than that of chimera 2+3 co-infection). This reduction, while not amounting to strong antagonism, may indicate a mild competitive or resource-limiting effect when two variants share the same cellular environment. This also supported by the confocal microscopy analysis, most of the fluorescences is not overlayed. The authors should consider discussing whether these slight decreases in titer reflect competition for limited host resources, replication sites, or other cellular factors, rather than simply stating "no evidence of synergism." A more nuanced interpretation—acknowledging a trend toward mild inhibition in some combinations—would strengthen the discussion.

Reviewer #2: (No Response)

Reviewer #3: The minor issue is the note that "The manuscript should sharpen the knowledge gap rather than simply stating that mixed infections matter". This aspect carries conceptual significance, whereas the novelty of this work lies specifically in the competition of nearly isogenic variants carrying distinct adaptive changes in a multifunctional protein, not in mixed infection per se. Within-host competition should be framed and extended.

**********

PLOS authors have the option to publish the peer review history of their article (what does this mean?). If published, this will include your full peer review and any attached files.

If you choose “no”, your identity will remain anonymous but your review may still be made public.

**Do you want your identity to be public for this peer review?**  For information about this choice, including consent withdrawal, please see our Privacy Policy.

Reviewer #1: No

Reviewer #2: No

Reviewer #3: No

[NOTE: If reviewer comments were submitted as an attachment file, they will be attached to this email and accessible via the submission site. Please log into your account, locate the manuscript record, and check for the action link "View Attachments". If this link does not appear, there are no attachment files.]

**Figure resubmission:**

**Reproducibility:**

To enhance the reproducibility of your results, we recommend that authors of applicable studies deposit laboratory protocols in protocols.io, where a protocol can be assigned its own identifier (DOI) such that it can be cited independently in the future. Additionally, PLOS ONE offers an option to publish peer-reviewed clinical study protocols. Read more information on sharing protocols at https://plos.org/protocols?utm_medium=editorial-email&utm_source=authorletters&utm_campaign=protocols

# Author comment: Author response to Decision Letter 2

**DOI:** 10.1371/journal.ppat.1014529.r004

5 Aug 2026

Attachment Submitted filename: Response to reviewers letter.docx

# Editor report: Decision Letter 2

**DOI:** 10.1371/journal.ppat.1014529.r005

**Authors:** Shou-Wei Ding, Xian-Bing Wang

6 Aug 2026

Dear Dr. Canto,

We are pleased to inform you that your manuscript 'Competition among co-infecting potyviral chimeras with adaptive, single-nucleotide differences in HCPro: properties, and influence of environment factors on outcomes' has been provisionally accepted for publication in PLOS Pathogens.

Before your manuscript can be formally accepted you will need to complete some formatting changes, which you will receive in a follow up email. A member of our team will be in touch with a set of requests.

Please note that your manuscript will not be scheduled for publication until you have made the required changes, so a swift response is appreciated.

IMPORTANT: The editorial review process is now complete. PLOS will only permit corrections to spelling, formatting or significant scientific errors from this point onwards. Requests for major changes, or any which affect the scientific understanding of your work, will cause delays to the publication date of your manuscript.

Should you, your institution's press office or the journal office choose to press release your paper, you will automatically be opted out of early publication. We ask that you notify us now if you or your institution is planning to press release the article. All press must be co-ordinated with PLOS.

Thank you again for supporting Open Access publishing; we are looking forward to publishing your work in PLOS Pathogens.

Best regards,

Xian-Bing Wang

Academic Editor

PLOS Pathogens

Shou-Wei Ding

Section Editor

PLOS Pathogens

Sumita Bhaduri-McIntosh

Editor-in-Chief

PLOS Pathogens

orcid.org/0000-0003-2946-9497

Michael Malim

Editor-in-Chief

PLOS Pathogens

orcid.org/0000-0002-7699-2064

***********************************************************

Reviewer Comments (if any, and for reference):

# Editor report: Acceptance letter

**DOI:** 10.1371/journal.ppat.1014529.r006

**Authors:** Shou-Wei Ding, Xian-Bing Wang

Dear Dr. Canto,

We are delighted to inform you that your manuscript, "Competition among co-infecting potyviral chimeras with adaptive, single-nucleotide differences in HCPro: properties, and influence of environment factors on outcomes," has been formally accepted for publication in PLOS Pathogens.

We have now passed your article onto the PLOS Production Department who will complete the rest of the pre-publication process. All authors will receive a confirmation email upon publication.

The corresponding author will soon be receiving a typeset proof for review, to ensure errors have not been introduced during production. Please review the PDF proof of your manuscript carefully, as this is the last chance to correct any scientific or type-setting errors. Please note that major changes, or those which affect the scientific understanding of the work, will likely cause delays to the publication date of your manuscript. Note: Proofs for Front Matter articles (Pearls, Reviews, Opinions, etc...) are generated on a different schedule and may not be made available as quickly.

Soon after your final files are uploaded, the early version of your manuscript, if you opted to have an early version of your article, will be published online. The date of the early version will be your article's publication date. The final article will be published to the same URL, and all versions of the paper will be accessible to readers.

For Research Articles, you will receive an invoice from PLOS for your publication fee after your manuscript has reached the completed accept phase. If you receive an email requesting payment before acceptance or for any other service, this may be a phishing scheme. Learn how to identify phishing emails and protect your accounts at https://explore.plos.org/phishing.

Thank you again for supporting open-access publishing; we are looking forward to publishing your work in PLOS Pathogens.

Best regards,

Sumita Bhaduri-McIntosh

Editor-in-Chief

PLOS Pathogens

orcid.org/0000-0003-2946-9497

Michael Malim

Editor-in-Chief

PLOS Pathogens

orcid.org/0000-0002-7699-2064
